# Novel Binding Partners and Differentially Regulated Phosphorylation Sites Clarify Eps8 as a Multi-Functional Adaptor

**DOI:** 10.1371/journal.pone.0061513

**Published:** 2013-04-23

**Authors:** Debbie L. Cunningham, Andrew J. Creese, Giulio Auciello, Steve M. M. Sweet, Tulin Tatar, Joshua Z. Rappoport, Melissa M. Grant, John K. Heath

**Affiliations:** 1 Cancer Research UK Growth Factor Signalling Group, School of Biosciences, College of Life and Environmental Sciences, University of Birmingham, Birmingham, United Kingdom; 2 School of Dentistry, College of Medical and Dental Sciences, University of Birmingham, Birmingham, United Kingdom; 3 School of Biosciences, College of Life and Environmental Sciences, University of Birmingham, Birmingham, United Kingdom; Bioinformatics Institute, Singapore

## Abstract

Eps8 is involved in both cell signalling and receptor trafficking. It is a known phosphorylation substrate for two proteins involved in the fibroblast growth factor receptor (FGFR) signalling pathway: the receptor itself and Src. Here we report a differential proteomic analysis of Eps8 aimed to identify specific FGFR and Src family kinase dependent phosphosites and co-associated phosphodependent binding partners. This study reveals a total of 22 Eps8 pTyr and pSer/Thr phosphorylation sites, including those that are dependent on Src family and FGFR kinase activity. Peptide affinity purification of proteins that bind to a selection of the pTyr phosphosites has identified a range of novel Eps8 binding partners including members of the intracellular vesicle trafficking machinery (clathrin and AP-2), proteins which have been shown to regulate activated receptor trafficking (NBR1 and Vav2), and proteins involved in receptor signalling (IRS4 and Shp2). Collectively this study significantly extends the understanding of Eps8 post-translational modification by regulated phosphorylation, identifies novel Eps8 binding partners implicated in receptor trafficking and signalling, and confirms the functions of Eps8 at the nexus of receptor signalling and vesicular trafficking.

## Introduction

Eps8 is involved in modulating cell signalling and receptor trafficking, via its range of protein interactions. When bound in a complex with Abi1and Sos1, Eps8 participates in signal transduction from Ras to Rac, leading to actin remodelling [1]. The SH3 domain of Eps8 binds Abi1 [1], [2] and, essential to its role in Rac activation, Sos1 binds the C-terminal effector region [3]. Coexpression of this Eps8-Abi1-Sos1 tri-complex has been correlated with advanced stage ovarian cancer, shown to be attributed to increased Rac-induced cell migration [4]. Interaction with the RabGAP, RN-Tre, via its SH3 domain, disrupts this tri-complex enabling Eps8 to participate in receptor trafficking via de-activation of Rab5 [5]. In addition, Eps8 is involved in actin capping and bundling via its interactions with IRSp53 and monomeric actin [6], [7].

Eps8 was originally identified as a novel phosphorylation substrate for the epidermal growth factor receptor (EGFR) and is also phosphorylated upon activation of other tyrosine kinases including fibroblast growth factor receptor (FGFR), platelet-derived growth factor (PDGF) and erbB-2 [8]. It has since been identified as a phosphorylation substrate for Src [9] and elevated expression of Eps8 has been observed in v-Src transformed cells [9], [10] and a variety of human cancers [11], [12], [13]. Phosphorylation is an important post-translational modification in the regulation of protein-protein interactions constituting cellular signal transduction, and aberrant regulation of phosphorylation can lead to malignancy. Indeed, constitutive phosphorylation of Eps8 has been found in a range of tumour cell lines [14].

Previously, we used quantitative proteomics to identify candidate mediators of FGFR signalling which are targets for Src family kinase (SFK)–mediated phosphorylation and functionally implicated in trafficking of activated FGFRs [15]. Eps8 was one such protein identified in this survey.

Collectively these features identify Eps8 as a potential target for transmitting FGFR and Src mediated signalling events to downstream effectors which warranted a detailed investigation of both FGFR and SFK mediated phosphorylation of Eps8 and analysis of phospho-dependent Eps8 binding partners to identify further candidate effectors and provide some insight into the possible pathways that these phosphorylation events influence. Using quantitative mass spectrometry techniques [16], [17], [18] coupled with chemical inhibition of FGFR and SFK kinase activity we have carried out phosphopeptide mapping of Eps8 in order to identify FGFR and SFK-regulated phosphorylation sites. In addition, differentially recruited phosphodependent protein partners have been identified using quantitative peptide pull down (PPD) assays. This technique has revealed many novel Eps8 binding partners including insulin-receptor substrate 4 (IRS4). Previous proteomic studies have implicated IRS4 in FGFR signalling [19], [20]. Here we have identified IRS4 as a novel binding partner for an Eps8 peptide containing phosphorylated Tyr252. Furthermore, we show that the interaction between Eps8 and IRS4 and their colocalisation within cells is increased following FGFR activation which coincides with tyrosine phosphorylation of both Eps8 and IRS4.

These results significantly expand the range of proteins implicated to interact with Eps8, illustrating further its role as a multi-functional adaptor molecule mediating FGFR and Src kinase signalling.

## Materials and Methods

### Cell Culture

Human embryonic kidney epithelial 293T cells and mouse NIH 3T3s were cultured at 37°C, 5% CO_2_ in DMEM containing 2 mM L-Glutamine (Lonza), supplemented with 0.1 mg/ml streptomycin, 0.2 U/ml penicillin (Sigma), and 10% v/v fetal calf serum (Labtech International). For SILAC labelling, 293T cells were cultured in SILAC DMEM (Thermo Fisher Scientific) supplemented with either 0.1 mg/ml “light” isotopically normal L-Lysine and L-Arginine (R0K0) (Sigma), “medium” ^13^C_6_ L-Lysine and 4,4,5,5-D4 L-Lysine (R6K4), or “heavy” ^13^C_6_^15^N_4_ L-Arginine and ^13^C_6_^15^N_2_ L-Lysine (R10K8) (Goss Scientific), 0.5 mg/ml proline (Sigma), 0.1 mg/ml streptomycin, 0.2 U/ml penicillin, and 10% v/v dialysed fetal bovine serum (Labtech International).

### Cloning and Transfection

The human open reading frames for Eps8 and IRS4 were supplied in Gateway (Invitrogen™) pDONR vectors from Open Biosystems. The insert encoding Eps8 was cloned into the Gateway compatible mammalian expression vector, Myc-PRK5 (gift from Laura Machesky) using Gateway cloning. The insert encoding IRS4 was cloned into the Gateway mammalian expression vector, pDEST53 (GFP-tag) using Gateway cloning. Eps8-mCherry was a gift from Giorgio Scita (IFOM University of Milan, Milan, Italy). HEK 293T cells were transfected using Genejuice (Novagen) and NIH 3T3 cells were transfected using Lipofectamine2000 (Invitrogen) according to manufacturer’s instructions. Cells were allowed to overexpress transfected protein for 48 h.

### Cell Treatment and Cell Lysis

Following overnight serum starvation in media containing 0.1% serum, cells were either pre-treated with SU6656, dasatinib or SU5402 for 30 min, followed by addition of 20 ng/ml FGF2, or treated as above in the absence of chemical inhibitor. For experiments where cells were treated with sodium pervanadate, 2 mM sodium pervanadate was added to the media for 20 min prior to FGF2 stimulation. Cell lysis and measurement of total protein concentrations were performed as described previously [15].

### Immunoprecipitation and Western Blotting

Antibodies were purchased from Roche (Myc 9E10), Santa Cruz (Eps8 sc-1841; Clathrin HC sc-12734; Vav2 sc-20803; Shp2 sc-424; IRS4 sc-100854; AP-2 sc-10761; PHB sc-28259; PHB2 sc-133094; ERK sc-94; p-ERK sc-7383), NEB (Src 2110, p-Src 2101, STAT3 9139), and Abcam (NBR1 ab55474). For IPs, antibodies were conjugated to Protein G Dynabeads (Invitrogen), as per manufacturer’s instructions. For SILAC anti-Myc IPs, anti-Myc antibody was further cross-linked to the beads: the conjugated Dynabeads were washed twice in a 10-fold excess of 0.2 M triethanolamine pH8.2. Beads were then resuspended in a 10-fold excess of freshly prepared 20 mM dimethyl pimelidate dihydrochloride (DMP) (Sigma) and mixed for 30 mins at room temperature followed by washing in a ten-fold excess of 50 mM Tris/HCl for 15 mins at room temperature and further washes (x3) in a 10-fold excess of PBS/0.1% Tween 20 (PBS-T). Cross-linked beads were resuspended in PBS, prior to addition of whole cell lysate (WCL). WCLs were mixed at 4°C with anti-Myc beads for 30 min, prior to washing. For SILAC experiments, WCLs from the heavy, medium, and light cell populations were immunoprecipitated separately (10 mg WCL) and beads were mixed following five washes in a 20-fold excess of lysis buffer. Following addition of reduced sample buffer, samples were boiled for 5 min, run on 4–12% Bis-Tris gels (Invitrogen) and Coomassie stained. Western blotting was performed as previously described [15].

### Peptide Pull Downs

Phosphorylated and non-phosphorylated peptide pairs were synthesised by Alta Bioscience, Birmingham, UK. Each peptide was synthesised with an N-terminal desthiobiotin. Peptides were bound to MyOne Streptavidin Dynabeads (Invitrogen) (2.5 µg/50 µl beads) by incubating at room temperature for 1 hour. Peptide-bound beads were washed for 15 min×5 in a 20-fold excess of PBS-T. Heavy SILAC labelled (R10K8) WCL (10 mg) was incubated with 50 µl beads bound to the phosphorylated peptide, medium SILAC labelled (R6K4) WCL (10 mg) was incubated with 50 µl beads bound to the non-phosphorylated peptide, and light SILAC labelled (R0K0) WCL (10 mg) was incubated with 50 µl beads without peptide, overnight at 4°C. PPDs were washed at least 5 times in a 20-fold excess of PBS-T. Beads from each peptide pair and a non-peptide control were combined. Following addition of reduced sample buffer, protein samples were run on 4–12% Bis-Tris gels (Invitrogen) and Coomassie stained.

### Trypsin Digestion and Phosphopeptide Enrichment of Samples

Trypsin digestion was carried out as previously described [15], [21]. From the excised band corresponding to Eps8, phosphopeptides were enriched using TiO_2_ tips (GLSciences), according to the manufacturer’s instructions. All resulting peptide mixtures were analysed by liquid chromatography tandem mass spectrometry (LC-MS/MS).

### Mass Spectrometry

On-line liquid chromatography was performed by use of an Dionex Ultimate 3000 LC system (Thermo Fisher Scientific). Peptides were loaded onto an Acclaim PepMap 100 C18 resolving column (15 cm length;75 µm internal diameter; LC Packings, USA) and separated over a 40 minute gradient from 3.2% to 44% acetonitrile (Baker, Holland). Peptides eluted directly (350 nL/min) via a Triversa nanospray source (Advion Biosciences, NY, USA) into a LTQ Orbitrap Velos mass spectrometer (Thermo Fisher Scientific). The mass spectrometer alternated between a full FT-MS scan (m/z 380-1600) and subsequent CID MS/MS scans of the twenty most abundant ions. Survey scans were acquired in the Orbitrap cell with a resolution of 60,000 at m/z 400. Precursor ions were isolated and subjected to CID in the linear ion trap. Isolation width was 2 Th. Only multiply-charged precursor ions were selected for MS/MS. CID was performed with helium gas at a normalized collision energy of 35%. Precursor ions were activated for 10 ms. Data acquisition was controlled by Xcalibur 2.1 software.

### Identification and Quantification of Peptide and Proteins

Mass spectra were processed using the MaxQuant software (version 1.0.13.13) [22], [23]. Data were searched, using MASCOT version 2.2 (Matrix Science), against a concatenated database consisting of the human IPI database (version 3.72) supplemented with common contaminants (including keratins, trypsin, BSA) and the reversed-sequence version of the same database. The human database contained 173,046 protein entries (86,523 of which were reversed-sequence versions). The search parameters were: minimum peptide length 6, peptide tolerance 7 ppm, mass tolerance 0.5 Da, cleavage enzyme trypsin/P, and a total of 2 missed cleavages were allowed. Carbamidomethyl (C) was set as a fixed modification and oxidation (M), acetylation (Protein N-term), Phospho (ST), and Phospho (Y) were set as variable modifications. The appropriate SILAC labels were selected and the maximum labelled amino acids was set to 3.

All experiments were filtered to have a peptide and protein false-discovery rate (FDR) below 1%. Within the MaxQuant output, phosphorylation sites were considered to be localised correctly if the localisation score (PTM score) was at least 0.85 (85%). Additional information is listed in Method S1.

### Confocal Microscopy and Quantification Analysis

Cells were fixed in 4% paraformaldehyde (PFA; Electron Microscopy Sciences) in PBS for 10 min prior to analysis. Confocal laser microscopy was performed with an inverted microscope (Zeiss LSM 710) using a 40×1.3NA oil-immersion objective and a Transmission-Photomultiplier LSM T-PMT. Data analysis was performed using NIS-Elements Imaging Software version 3.2 (Nikon). The experiment was repeated 3 times and an image that represented the phenotype of most of the cells was selected.

## Results and Discussion

### Effects of SU5402 and Dasatinib on Eps8 Phosphorylation

**Figure 1:**
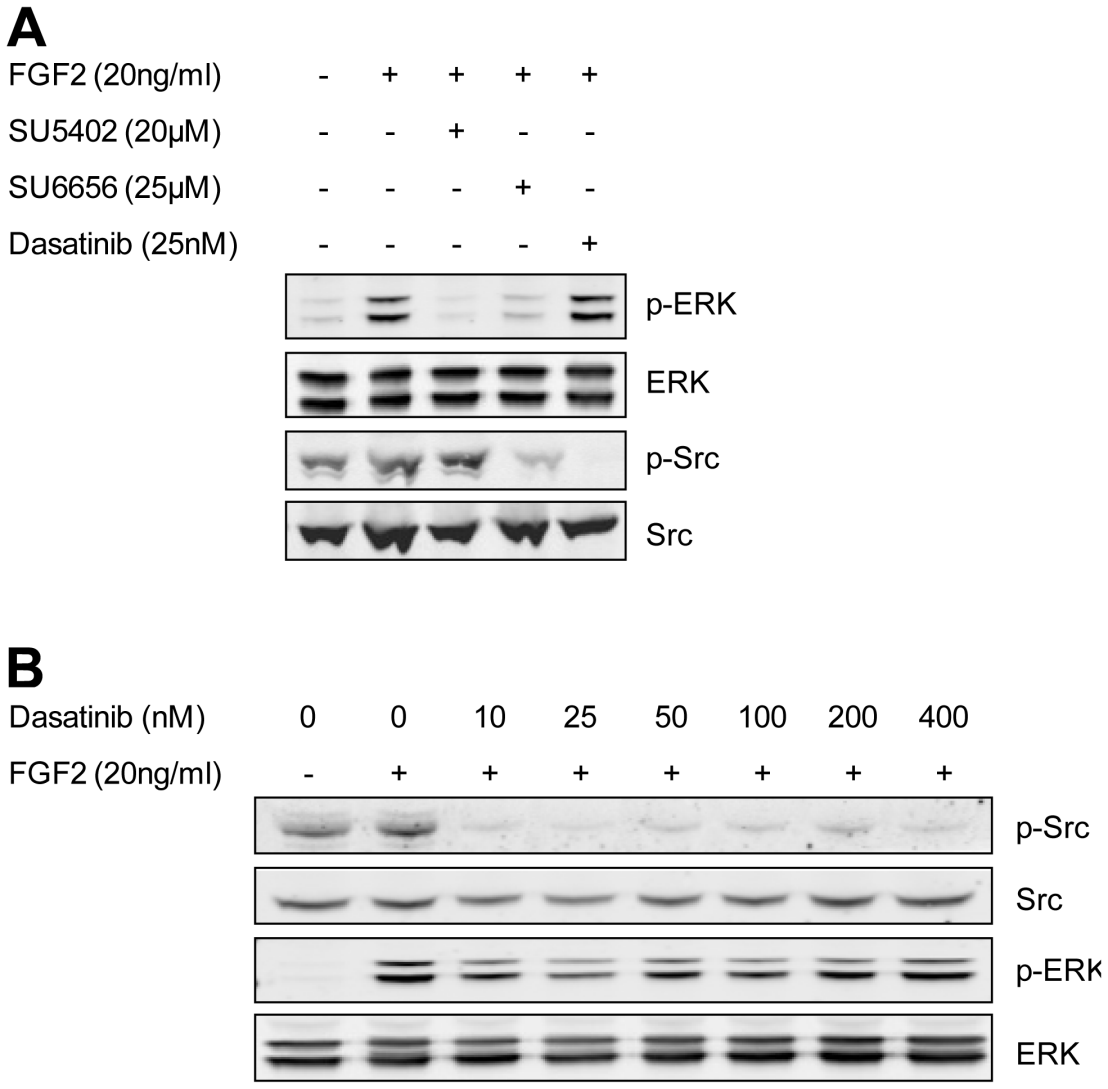
Chemical inhibition of Src kinase and FGFR kinase activity. A) HEK 293T cells were treated with SU5402, SU6656, or dasatinib 30 min prior to addition of FGF2 for 15 min. Cells were lysed and analysed by western blotting. B) HEK 293T cells were treated with increasing concentrations of dasatinib for 30 min prior to addition of FGF2 for 15 min. Cells were lysed and analysed by western blotting.

Eps8 is a phosphorylation substrate for FGFR [8] and Src [9]. To identify the residues upon which these phosphorylation events take place we have used a targeted mass spectrometric approach. A quantitative SILAC technique coupled with chemical inhibition of FGFR or SFK activity has been used to examine any differential regulation in the phosphorylation of Eps8. Dasatinib is an SFK/ABL kinase inhibitor approved for use in patients with chronic myelogenous leukemia [24]. Here, dasatinib has been used to inhibit SFK activity in preference to SU6656 [25], which was our previous inhibitor of choice prior to dasatinib becoming commercially available [15]. At concentrations needed to inhibit Src kinase activity in HEK 293T cells, SU6656 also shows some inhibition of FGFR activation, as measured by levels of phosphorylated ERK (Figure 1A). Dasatinib does not show inhibition of FGFR induced ERK activity, even at high concentrations (Figure 1B). SU5402, has been used to inhibit the tyrosine kinase activity of FGFR1 [26] and does not have any effect on phospho-Src levels (Figure 1A). Using these compounds has allowed us to differentiate between SFK-mediated and FGFR-mediated phosphorylation events on Eps8 by comparing levels of phosphorylation in the presence of dasatinib and SU5402.

We transfected three populations of HEK 293T cells, grown in either ‘Light’ R0K0, ‘Medium’ R6K4, or ‘Heavy’ R10K8 SILAC media, with Myc-Eps8. Cells were either left untreated (R0K0) or pre-treated with SU5402 (R6K4) or dasatinib (R10K8) for 30 min before stimulation with FGF2 for a further 15 min. Prior to mass spectrometric analysis, phosphopeptide enrichment was carried out on immunoprecipitated Myc-Eps8.

**Figure 2:**
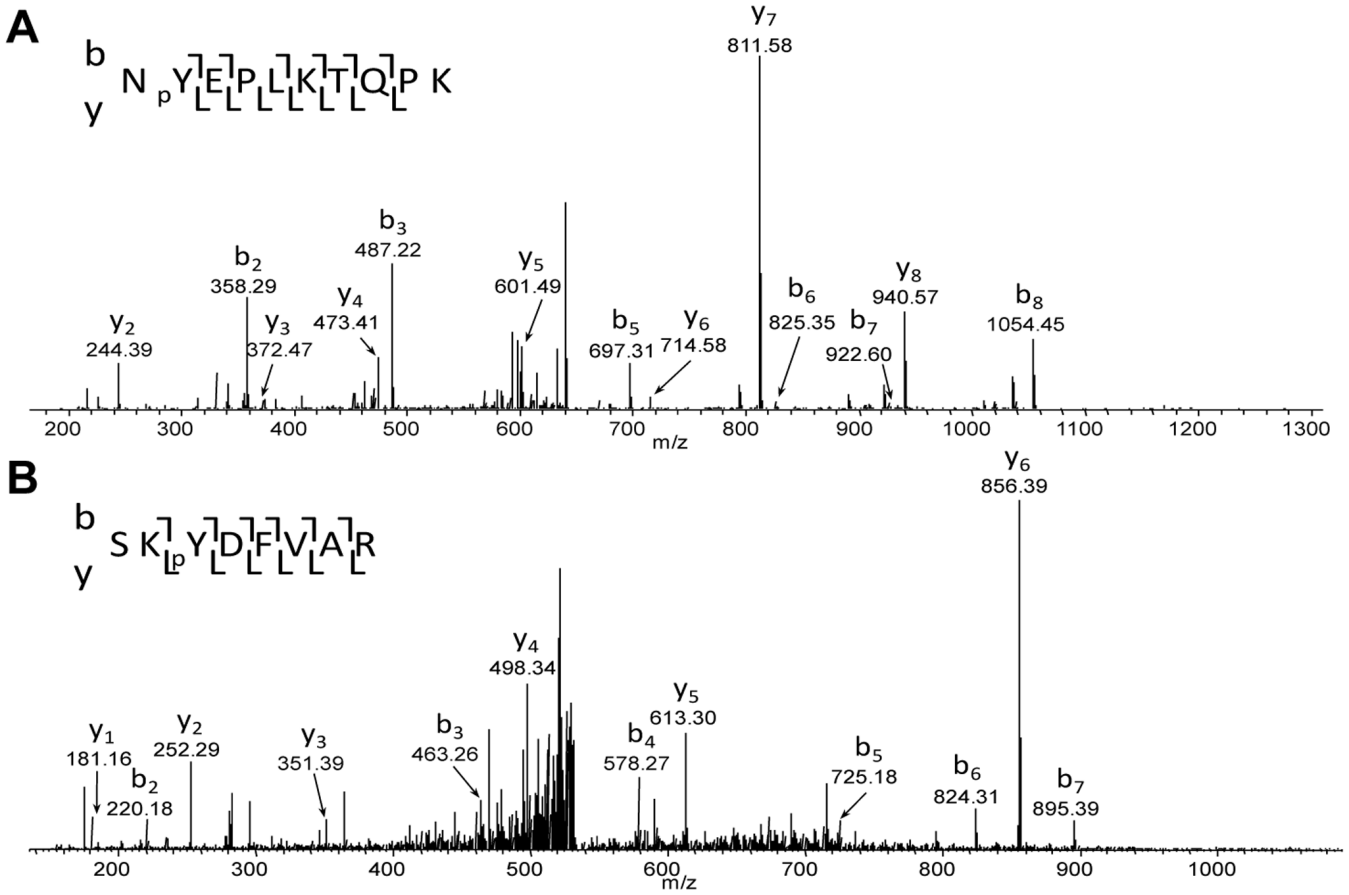
Representative mass spectra for identification and site localisation of tyrosine phosphorylation on Eps8. A) Eps8 is phosphorylated on residue 525. B) Eps8 is phosphorylated on residue 540. pY indicates phosphotyrosine.

**Figure 3:**
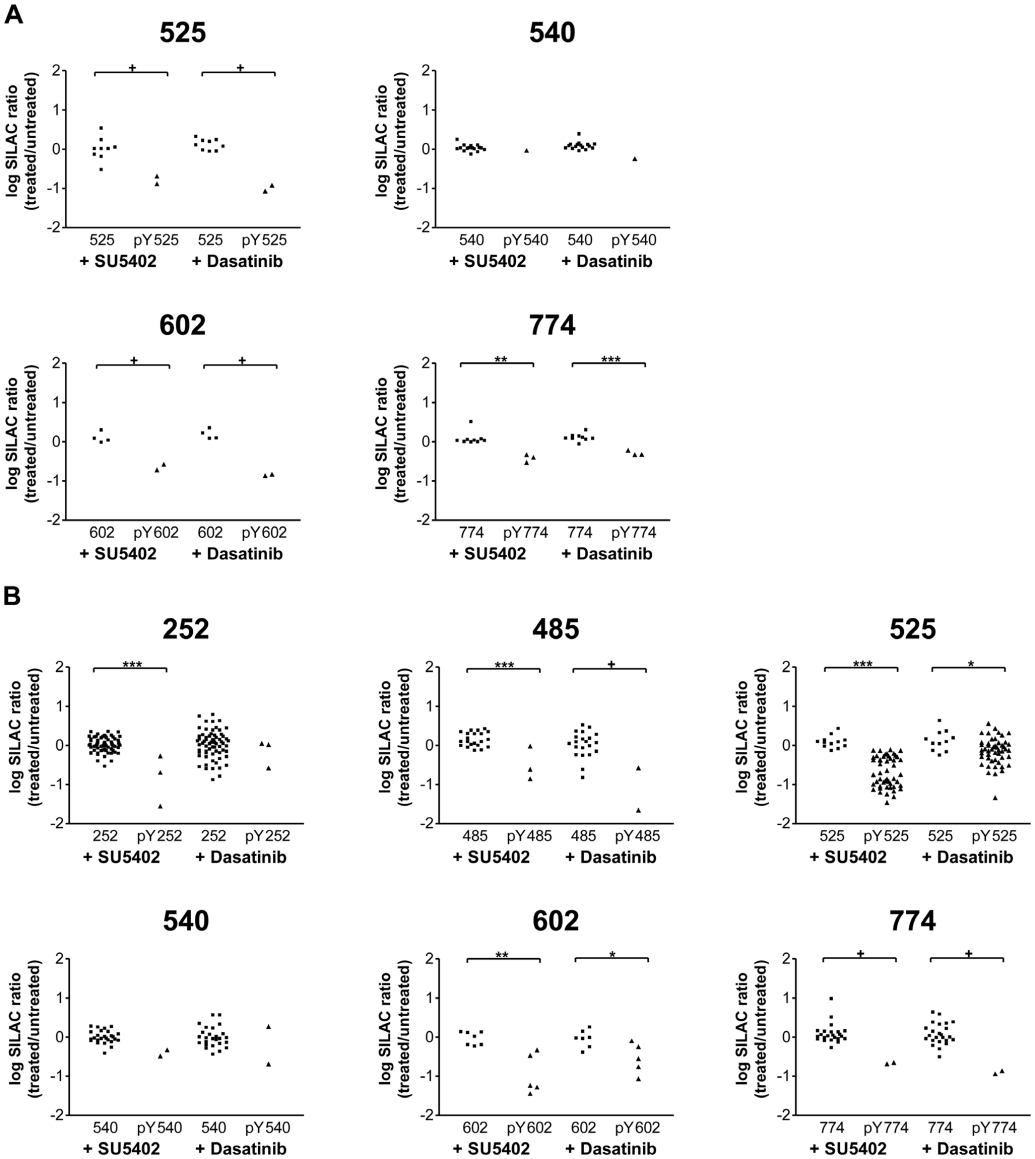
Differential regulation of tyrosine phosphorylation on Eps8. Heavy, medium and light SILAC labelled HEK 293T cells were treated with either 25 nM dasatinib, 20 µM SU5402, or no inhibitor, prior to FGF2 stimulation (20 ng/ml; 15 min). Myc-Eps8 was immunoprecipitated and the resulting sample was run on an SDS-PAGE gel and, following in-gel trypsin digestion and phophopeptide enrichment, analysed by mass spectrometry. Each graph represents specific residues on Eps8 as indicated. Each data point represents a single peptide identification. *P* values were calculated by an unpaired t-test (0.01–0.05 =  *; 0.001–0.01 =  **; <0.001 =  ***). +, the median of the ratios is<the cut-off value of 0.57 and is deemed significantly changed (see Method S1). A) Experiment was carried out in the absence of sodium pervanadate. B) Experiment was carried out in the presence of 2 mM sodium pervanvadate.

A total of 22 distinct sites of phosphorylation (18 serines and 4 tyrosines) on Eps8 were identified (Table S1). Representative mass spectra for two of the identified phosphopeptides are shown in Figure 2 (see Figure S3 for additional spectra). The log-ratios for each identification of phosphorylated peptide and non-phosphorylated counterpart peptide are plotted in Figure 3 (tyrosine residues) and Figure S1 (serine residues) and provide a visual comparison of the relative ratios and number of peptide identifications between non-treated and inhibitor treated samples. Statistical methods for determining differential phosphorylation are discussed by de la Fuente van Bentem *et al.* [27]. Our preferred statistical method for deciding differential phosphorylation between samples is to use a t-test to directly compare non-phosphorylated and phosphorylated counterpart peptides. However, the t-test has only been applied when there are three or more replicates for each peptide. Another method to determine differential phosphorylation is to select a cut-off for significant statistics which is based on p-values determined from a test sample (Method S1). This has been applied when only two identifications have been made. Of the 4 tyrosine residues identified, 3 phosphotyrosine containing peptides (pY525, pY602, pY774) had reduced SILAC ratios compared to their unphosphorylated counterpart in the presence of both SU5402 and dasatinib, indicating that the tyrosine phosphorylation on these particular sites within Eps8 are sensitive to both FGFR and SFK activity (Figure 3A).

Eps8 contains 20 tyrosine residues, and according to the PhosphositePlus database [28], 9 of them have been found in their phosphorylated form in the human protein. It may be that under our experimental conditions in which the cells were stimulated with FGF2, only the 4 tyrosines that we have identified are phosphorylated. However, it is also possible that the levels of some phosphopeptides remain too low for mass spectrometric detection. Thus, in an attempt to increase the number of phosphorylated tyrosine residues identified, prior to FGF2 stimulation, cells were treated with sodium pervanadate to inhibit tyrosine phosphatase activity and maximise the levels of tyrosine phosphorylated peptides. Under these conditions, an additional 3 phosphotyrosine containing peptides were identified, and the majority of the previously identified phosphopeptides were present in greater abundance (Figure 3B). Several additional serine residues were also identified (Figure S1). Of the tyrosine residues identified, 5 phosphotyrosine peptides (pY252, pY485, pY525, pY540, pY602, pY774) had a reduced SILAC ratio in the presence of SU5402 indicating that these particular sites within Eps8 are regulated by FGFR kinase activity (Figure 3B). Four (pY485, pY525, pY602, pY774) also had a reduced SILAC ratios in the presence of dasatinib, indicating that these sites are regulated by SFK activity (Figure 3B). One phosphopeptide (pY454) was identified but was of too low abundance for ratio calculation. Data obtained in the absence and presence of sodium pervandate were in agreement in terms of the differential regulation of phosphorylation on these sites. In the absence of tyrosine phosphatase activity, the number of phosphotyrosine peptide identification events for site pY525 was hugely increased, suggesting that this site is readily phosphorylated but has a high turnover rate. Phosphorylation and dephosphorylation of proteins at distinct sites can act as a molecular switch regulating the association and disassociation of interacting proteins. It may be that the Y525 is an important regulatory site that is required to be turned over at a high frequency rate in order to allow Eps8 function.

Our experiments have identified 16 of the 20 tyrosines present within the human form of Eps8, the remainder being in regions of the protein that were not detected. The total sequence coverage of Eps8 is 68%. In the presence of FGF2, 7 of these tyrosine residues are phosphorylated and for 6 of these a SILAC ratio could be calculated. Residue pY540 shows no change in phosphorylation due to the presence of the inhibitors, residue pY252 shows a decrease in phosphorylation only in the presence of the FGFR inhibitor, and the phosphorylation on residues pY485, pY525, pY602, and pY774 are decreased in the presence of both the FGFR and SFK inhibitor.

### Phosphotyrosine Specific Interactions

**Figure 4:**
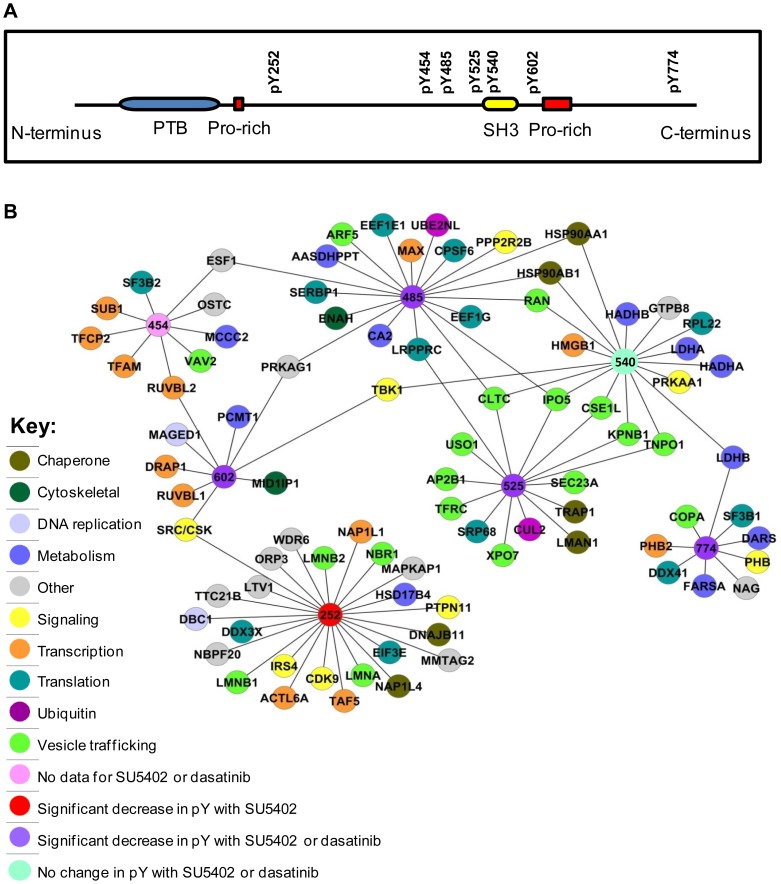
Protein-peptide interaction network for proteins binding specifically to phosphotyrosine-containing Eps8 peptides. A) Schematic diagram showing locations of the pY residues within the domain structure of Eps8. B) Using SILAC we carried out quantitative peptide pull-down assays from FGF2 stimulated (20 ng/ml; 15 min) HEK 293T cells to compare protein-peptide interactions for phosphotyrosine versus non-phosphotyrosine containing Eps8 peptides. Proteins interacting preferentially to phosphotyrosine peptides have been plotted in an interaction network.

In the presence of FGF2, we have identified specific sites of tyrosine phosphorylation on Eps8. We have demonstrated differential phosphorylation events on a number of these sites that are dependent on activity of FGFR or SFKs. The distribution of these sites on Eps8 is shown graphically in Figure 4A. Our aim was to then further characterise these sites by identifying phospho-dependent protein binding partners. To identify such potential pTyr-dependent interacting partners for these residues in Eps8, which may be involved in cellular processes downstream of FGFR, we used a quantitative proteomic peptide pulldown (PPD) approach [18]. Peptides containing the desired tyrosine residues were synthesised in their phosphorylated and non-phosphorylated forms. Using SILAC, we incubated heavy labelled (R10K8) HEK 293T cell lysates with phosphorylated peptide, medium labelled (R6K4) lysates with non-phosphorylated peptide, and light lysates (R0K0) were used as a no peptide control. All cells were treated with FGF2 for 15 min prior to lysis. The huge advantage of using SILAC over other techniques is that the specific binders can still be identified in the presence of many non-specific proteins. Typically a pull-down experiment will isolate not only specific interactors but also background proteins that are binding to the bead matrix. Proteins with SILAC ratios close to 1∶1 can be discarded and only those proteins that are significantly enriched in one of the populations are regarded as ‘hits’. To qualify as a pY-dependent binder in these PPD experiments, proteins had to have a H_pY_/M_non-pY_ and H_pY_/L_control_ ratio at least 2 standard deviations higher than the median (95% confidence). H/M and H/L ratios for all proteins identified in each PPD experiment are plotted in Figure S2. There are instances when peptides are identified only in the heavy state and, therefore, not assigned a ratio. In these cases, the mass spectra were manually checked to identify pY-peptide specific interactors. Eighty-five distinct proteins with a range of cellular functions were identified for the 7 pY-peptides (Figure 4B; Table S2).

We have identified a number of SH2 or PTB domain-containing proteins from our PPD assays which are likely to be direct interacting partners for our phosphotyrosine peptides. These proteins include known phosphotyrosine-binding proteins Shp2 (PTPN11), Vav2 and Insulin receptor substrate 4 (IRS4). In addition, four heavy labelled peptides identified as common to both Src and CSK were enriched in the pY-PPD for residues 252 and 602. From this data it is not possible to discriminate between these two proteins as they have high sequence homology, however, Eps8 is known to bind to Src [9]. STAT3, another SH2 domain containing protein, was identified in the pY525 PPD, with a significantly increased H_pY_/M_non-pY_ ratio (>95% confidence) and an increased H_pY_/L_control_ (>93% confidence). As both ratios were not >95% confidence, STAT3 was not included in Figure 4B and Table S2.

IRS4, Shp2 and WDR6 were identified as a potential novel binding partners for the pY252 peptide. IRS4 acts as an interface between receptor tyrosine kinases, such as IGF1R [29] and FGFR1 [19], and SH2-containing intracellular signalling molecules. It contains an IRS PTB domain, through which it can bind to phosphorylated proteins. IRS4 is known to interact with both Shp2 [30] and WDR6 [31], [32], [33], and all these proteins have been implicated in FGFR signalling [19], [20], [34].

Eps8 has been shown to regulate receptor endocytosis via its interaction with RN-Tre [5]. When bound to Eps8, RN-Tre, a RabGAP, acts on Rab5 to inhibit EGFR internalisation [5]. We have found several potential Eps8 binding proteins that also play a role in endocytosis. Clathrin heavy chain was enriched preferentially with pY485, pY525 and pY540 peptides, and a component of the adaptor protein complex 2 (AP-2), AP-2 complex subunit beta-1 (AP2B1), with the pY525 peptide. AP-2 is involved in clathrin-dependent endocytosis in which cargo proteins become incorporated into clathrin-coated vesicles (CCVs) which fuse with the early endosome. Recently Eps8 has been shown to be recruited to clathrin-coated structures at the plasma membrane [35] and, furthermore, we have found that FGFR activation promotes clathrin-mediated endocytosis through Eps8 and Src [36]. Vav2, found enriched for pY454, regulates EGFR receptor endocytosis and degradation [37] and NBR1, found enriched for pY252, regulates the degradation of receptor tyrosine kinases [38]. These proteins are potential novel Eps8 interactors that may act downstream of FGFR.

Proteins involved in vesicular trafficking to and from the Golgi apparatus include Arf5, a protein involved in vesicle budding from the Golgi, identified in the pY485 PPD, coatamer subunit alpha (COPA) in the pY774 PPD and both Sec23A and general vesicular transport factor p115 (USO1) in the pY525 PPD.

A cluster of proteins involved in nucleocytoplasmic transport were found bound to pY485, pY525, and pY540: the small GTPase Ran, Importin-5 (IPO5), importin subunit beta 1 (KPNB1), Exportin-2 (CSE1L), Exportin-7 (XPO7), and Transportin-1 (TNPO1). A number of these proteins are known to bind, either in isolation, or together with an adapter protein, to nuclear localisation signals (NLSs) in cargo proteins. As an NLS is a positively charged sequence, it is possible that these proteins can bind preferentially to peptides containing basic residues. Peptide 540 is a potential candidate for this, having 4 lysine residues in close proximity. However, each of these proteins is enriched specifically for the pY form of the peptide, arguing against lysine-dependent binding. Eps8 contains a putative NLS between residues 299–309 [8], but has not been reported to be present in the nucleus.

Out of the many proteins identified that have functions in protein trafficking, whether it be from the plasma membrane, to and from the Golgi, or to and from the nucleus, a significant number of them are associated with the pY525 peptide. Interestingly this is the site that we find to have a high turnover rate, suggesting a functional role in a dynamic cellular process.

MAPKAP1 (Sin1) was recruited to the pY252 peptide. MAPKAP1 interacts with mammalian target of rapamycin (mTOR) and is found in the mTOR complex 2 (mTORC2) [39], [40], [41]. Interestingly, Eps8 has been shown to regulate the expression of FAK via the mTor/STAT3 pathway [11]. Eps8 overexpression leading to increased activity of FAK via this pathway has been shown to promote disease progression in colon cancer [11]. MAPKAP1 may be a physical link to this pathway. As previously mentioned, STAT3 was also identified in the pY525 PPD. Additionally, STAT3 has been linked to FGFR in tumour cells where it can interact with amplified receptor [42].

Other potentially interesting putative Eps8 binding partners that we have identified that could link Eps8 to FGFR signalling include LRPPRC, which has previously been co-purified with the FGFR complex [43], and PHB which is required for Ras-mediated Raf-MEK-ERK activation [44].

Several RNA-binding proteins have been found in our PPDs. It has been reported previously that RNA-binding proteins can bind preferentially to the negative charge on the phosphorylated peptides, thus appearing as specific binders when they are actually contaminants [18].

### Novel Eps8 Binding Partners

Without further validation proteins identified in peptide pull-down assays can only be described as potential interactors. It may be that the interactions are not biologically relevant, or when the full length protein is in its folded conformation the interactions detected between peptide and protein are not sterically possible. In addition contaminants arising from interactions with the bead matrix may be present. Common IP contaminants, described as the ‘bead proteome’ are listed and scored in the ‘protein frequency library’ (used to identify the frequency with which proteins appear in a subset of IPs using a particular type of beads) [45], [46]. This library can help in discriminating between those proteins that are true interactors and those that may be bead contaminants. The majority of proteins identified both here, and with a high frequency (68–92%) in experiments compiled in the protein frequency library [46] are RNA-binding proteins: 60S ribosomal protein (RPL22), SERBP1, ATP-dependent RNA helicase DDX3X, splicing factor 3B subunits 1 and 2 (SF3B1, SF3B2), DEAD box protein 41 (DDX41). Hence further validation is required to identify ‘true’ protein-protein interactions.

**Figure 5:**
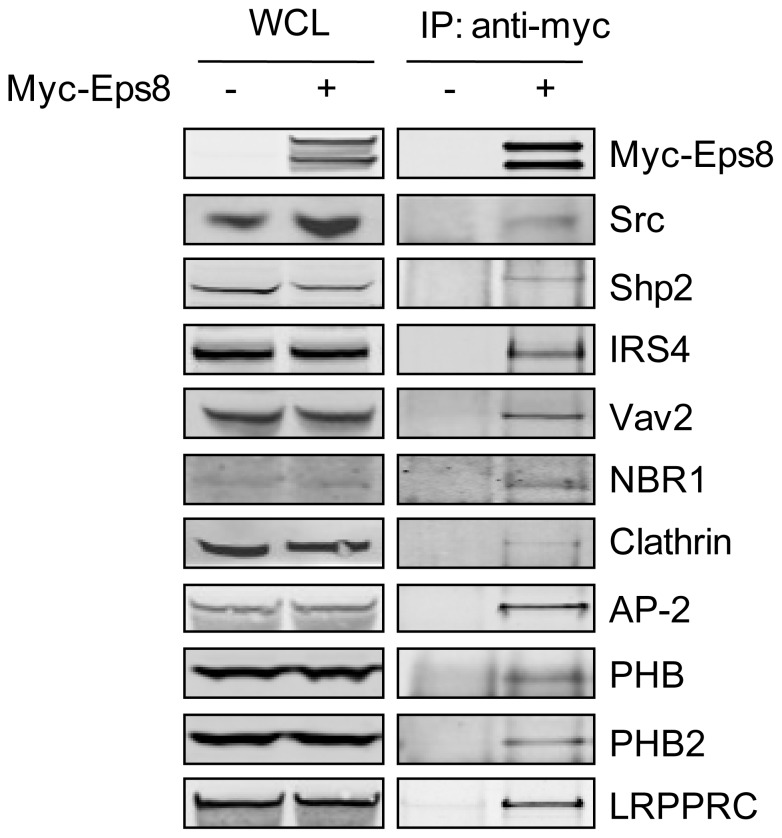
Full-length Eps8 interacts with a range of proteins identified in the peptide pull down assays. HEK 293T cells were either transfected with Myc-Eps8 or left untransfected. Cells were stimulated with 20 ng/ml FGF2 for 15 min and immunoprecipitated using an anti-Myc antibody. Western blot analysis was carried out on whole cell lysate and immunoprecipitation samples using antibodies against the indicated proteins.

In an attempt to further validate potential protein-protein interactions we used co-immunoprecipitation to pull down Myc-tagged Eps8 protein from FGF2 stimulated HEK 293T cells and probed for proteins of interest (Figure 5). The interactions between Eps8 and AP-2 and clathrin, proteins important in clathrin-mediated endocytosis, have been confirmed. This further supports evidence that Eps8 plays an important role in endocytic trafficking. In addition, we confirm the novel interactions between Eps8 and Shp2, Vav2, NBR1, LRPPRC, PHB, PHB2 and IRS4. It must be noted that, although co-immunoprecipitation experiments such as these can confirm protein-protein interactions it is acknowledged that they detect both direct and indirect interactions.

### The Novel Interaction between Eps8 and IRS4 is FGF2 Dependent

**Figure 6:**
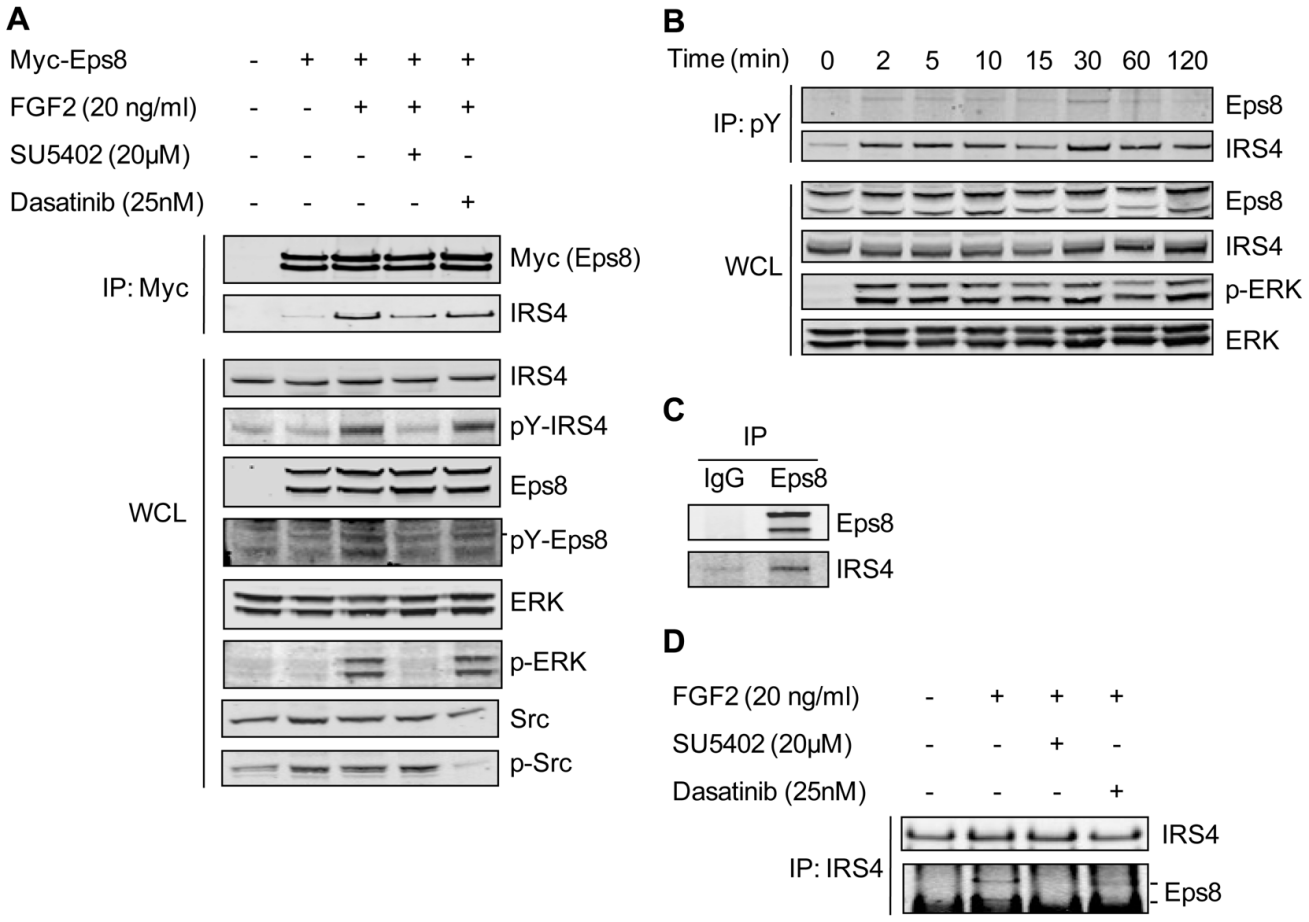
Eps8 and IRS4 interact in an FGF2 dependent manner that correlates with an increase in their tyrosine phosphorylation. A) HEK293T cells transfected with Myc-Eps8 were stimulated with 20 ng/ml FGF2 for 15 min either following 30 min pretreatment with SU5402 or dasatinib or in the absence of inhibitors. Anti-Myc immunoprecipitation and whole cell lysate samples were analysed by western blotting. B) HEK293T cells were stimulated with 20 ng/ml FGF2 for different lengths of time and tyrosine phosphorylated proteins were immunoprecipitated. Anti-pY immunoprecipitation and whole cell lysate samples were analysed by western blotting. C) HEK 293T cells were stimulated with 20 ng/ml FGF2 for 15 min and immnoprecipitations carried out using antibodies to either Eps8 or rabbit IgG. Resulting IP samples were analysed by western blotting. d) Following 30 min treatment with SU5402 or dasatinib and stimulation with 20 ng/ml FGF2 for a further 15 min, endogenous IRS4 was immunoprecipitated from HEK293T cells. Resulting IP samples were analysed by western blotting.

**Figure 7:**
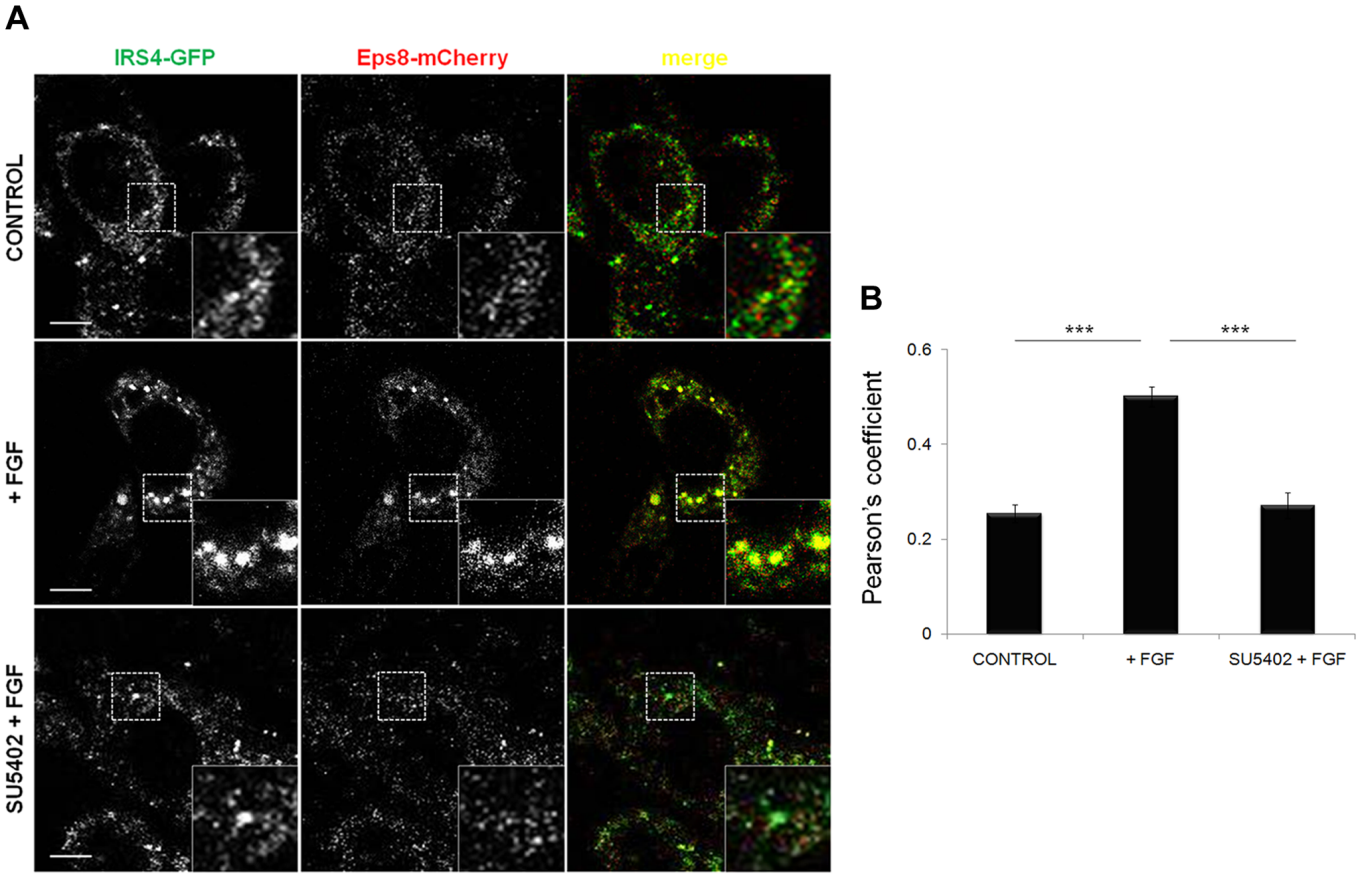
Eps8 and IRS4 colocalise within cells in an FGF2 dependent manner. NIH 3T3 cells were co-transfected with IRS4-GFP and Eps8-mCherry. Cells stimulated with FGF2 (20 ng/ml) in the presence and absence of SU5402 (25 µM) were compared to unstimulated cells (control). A) Confocal microscopy was used to visualise the localisation of IRS4 and Eps8. B) The colocalisation (Pearson’s coefficient) between IRS4 and Eps8 is significantly increased in the presence of FGF2 and absence of SU5402 (Pearson’s coefficient, mean±SEM, n = 42 cells. Scale bars = 5 µm. ***, P<0.001).

FGF2 activation of FGFR1 and FGF7 activation of FGFR2 results in phosphorylation of IRS4 on residue 921 [19], [20]. Phosphorylation of IRS4 promotes the formation of a complex with Shc, which may link IRS4 directly to activated receptor, and also allows recruitment of Grb2, PLCγ and PI3K thus promoting downstream signalling [19]. In our PPDs IRS4 was identified as a potential novel binding partner for the Eps8 peptide containing phosphorylated Tyr252, a residue shown to be sensitive to the addition of the FGFR kinase inhibitor, SU5402 and not the SFK inhibitor, dasatinib (Figures 3 and 4). Furthermore, the Eps8-IRS4 protein-protein interaction was confirmed by subsequent co-immunoprecipitation experiments with Myc-Eps8 in FGF2 stimulated cells (Figure 5). Next, as eluded to in the peptide pull down assays, we investigated whether the Eps8-IRS4 interaction within cells is dependent upon FGF2 activation and, therefore, sensitive to the addition of SU5402 but not dasatinib. Co-immunoprecipitation experiments were performed in the presence and absence of FGF2, SU5402 and dasatinib (Figure 6). The association between Myc-Eps8 and IRS4 increases in the presence of FGF2 (Figure 6A). Pre-treatment with SU5402 causes an inhibition of this interaction, indicating this increased association is due to activation of the FGF receptor. This FGF2 dependent increase in the association is not affected by the presence of dasatinib and, therefore, not dependent on SFK phosphorylation. An increase in phosphorylation of both Eps8 and IRS4 is seen upon FGF2 activation as previously reported [8], [19]. Both Eps8 and IRS4 are tyrosine phosphorylated in response to FGF2 in HEK 293T cells (Figure 6A) and remain so throughout the duration of receptor activation (Figure 6B). The interaction between Eps8 and IRS4 has been confirmed using endogenous levels of both proteins (Figure 6C). Co-immunoprecipitation of endogenous levels of Eps8 and IRS4 only in the presence of FGF2 and FGF2 with dasatinib, both conditions where FGF receptor is active, confirm that this interaction is FGF dependent (Figure 6D) as seen with overexpressed Eps8. Cell imaging data using fluorescently tagged proteins confirm these results. Following FGF2 stimulation, Eps8 colocalises with IRS4 in NIH 3T3 cells within the cytoplasm (Figure 7). This colocalisation is decreased following the addition of SU5402 indicating that it is dependent upon activated FGFR. These data, together with the peptide pull down data suggests that the interaction between Eps8 and IRS4 may occur on residue 252 of Eps8 when it is a phosphorylated state and whose phosphorylation is dependent upon FGFR activation.

### Conclusions

Here we have used quantitative proteomics to study the phosphorylation of Eps8 and phosphotyrosine dependent binding of proteins to it. Clusters of proteins with distinct cellular functions have been identified, including a large number involved in trafficking, either from the cell membrane, the Golgi or from cytoplasm to nucleus and vice versa. Most of the proteins identify are potential novel interactors, and further studies are needed to validate some of these interactions. The validated interactions between Eps8 and clathrin and AP-2, provide further evidence to support the role of Eps8 in receptor mediated endocytosis. Also, the interaction between Eps8 and IRS4, together with the knowledge that IRS4 is involved in downstream signalling from the FGF receptor, is an interesting lead into the role of Eps8 in FGFR signalling. In conclusion, Eps8 is a multi-functional adaptor protein which may have the capabilities of integrating receptor trafficking, cellular signalling, and protein degradation.

## Supporting Information

Figure S1 **Differential regulation of serine phosphorylation on Eps8.** Heavy, medium and light SILAC labelled HEK 293T cells were treated with either dasatinib, SU5402, or no inhibitor, prior to FGF2 stimulation. Myc-Eps8 was immunoprecipitated and the resulting sample was run on an SDS-PAGE gel and, following in-gel trypsin digestion and phophopeptide enrichment, analysed by mass spectrometry. Each graph represents specific residues on Eps8 as indicated. Each data point represents a single peptide identification. *P* values were calculated by an unpaired t-test (0.01–0.05 =  *; 0.001–0.01 =  **; <0.001 =  ***). +, the median of the ratios is outside the significance cut-off values (<0.57 or >1.75) and is deemed significantly changed (see) A) Experiment was carried out in the absence of sodium pervanadate. B) Experiment was carried out in the presence of 2 mM sodium pervanvadate.
(PDF)

Figure S2 **Relative SILAC ratios for proteins identified in peptide pull down assays.** Biotinylated Eps8 peptides containing the desired tyrosine residues (the residue number is indicated at the top of each plot) were synthesised in their phosphorylated and non-phosphorylated forms. Three peptide pull down assays for each residue were performed. Using SILAC, we incubated heavy labelled (R10K8) HEK 293T cell lysates with phosphorylated peptide, medium labelled (R6K4) lysates with non-phosphorylated peptide, and light lysates (R0K0) were used as a no peptide control. All cells were treated with FGF2 for 15 min prior to lysis. Following washing, the beads from each assay were combined, the resulting sample run on an SDS-PAGE gel and, following in-gel trypsin digestion, analysed by mass spectrometry. Each graph represents specific residues on Eps8 as indicated. Each data point represents a single protein identification and is shown as a function of its H_pY_/M_non-pY_ and H_pY_/L_control_ ratios. Those proteins with both ratios at least 2 standard deviations higher than the median (95% confidence) are shown in red and are considered pY-specific binders (these protein are named in Figure 4).
(PDF)

Figure S3 **Additional Mass Spectra of Phosphorylated Peptides Identified.**
(PDF)

Table S1 **Phosphorylated Peptides and non-Phosphorylated Counterpart Peptides Identified in Triple SILAC (+/− SU5402/dasatinib) Experiments.**
(XLS)

Table S2 **Proteins Identified as pY-specific binders in SILAC peptide pull down (PPD) experiments.**
(XLS)

Method S1 **Supplementary details regarding Peptide and Protein Identification and Quantification.**
(DOC)

## References

[1] Scita G, Nordstrom J, Carbone R, Tenca P, Giardina G, et al (1999) EPS8 and E3B1 transduce signals from Ras to Rac. Nature 401: 290–293. 10499589 10.1038/45822

[2] Biesova Z, Piccoli C, Wong WT (1997) Isolation and characterization of e3B1, an eps8 binding protein that regulates cell growth. Oncogene 14: 233–241. 9010225 10.1038/sj.onc.1200822

[3] Scita G, Tenca P, Areces LB, Tocchetti A, Frittoli E, et al (2001) An effector region in Eps8 is responsible for the activation of the Rac-specific GEF activity of Sos-1 and for the proper localization of the Rac-based actin-polymerizing machine. J Cell Biol 154: 1031–1044. 11524436 10.1083/jcb.200103146 PMC2196181

[4] Chen H, Wu X, Pan ZK, Huang S (2010) Integrity of SOS1/EPS8/ABI1 tri-complex determines ovarian cancer metastasis. Cancer Res 70: 9979–9990. 21118970 10.1158/0008-5472.CAN-10-2394 PMC3059077

[5] Lanzetti L, Rybin V, Malabarba MG, Christoforidis S, Scita G, et al (2000) The Eps8 protein coordinates EGF receptor signalling through Rac and trafficking through Rab5. Nature 408: 374–377. 11099046 10.1038/35042605

[6] Disanza A, Carlier MF, Stradal TE, Didry D, Frittoli E, et al (2004) Eps8 controls actin-based motility by capping the barbed ends of actin filaments. Nat Cell Biol 6: 1180–1188. 15558031 10.1038/ncb1199

[7] Disanza A, Mantoani S, Hertzog M, Gerboth S, Frittoli E, et al (2006) Regulation of cell shape by Cdc42 is mediated by the synergic actin-bundling activity of the Eps8-IRSp53 complex. Nat Cell Biol 8: 1337–1347. 17115031 10.1038/ncb1502

[8] Fazioli F, Minichiello L, Matoska V, Castagnino P, Miki T, et al (1993) Eps8, a substrate for the epidermal growth factor receptor kinase, enhances EGF-dependent mitogenic signals. EMBO J 12: 3799–3808. 8404850 10.1002/j.1460-2075.1993.tb06058.x PMC413663

[9] Maa MC, Lai JR, Lin RW, Leu TH (1999) Enhancement of tyrosyl phosphorylation and protein expression of eps8 by v-Src. Biochim Biophys Acta 1450: 341–351. 10395945 10.1016/s0167-4889(99)00069-5

[10] Gallo R, Provenzano C, Carbone R, Di Fiore PP, Castellani L, et al (1997) Regulation of the tyrosine kinase substrate Eps8 expression by growth factors, v-Src and terminal differentiation. Oncogene 15: 1929–1936. 9365239 10.1038/sj.onc.1201344

[11] Maa MC, Lee JC, Chen YJ, Lee YC, Wang ST, et al (2007) Eps8 facilitates cellular growth and motility of colon cancer cells by increasing the expression and activity of focal adhesion kinase. J Biol Chem 282: 19399–19409. 17496330 10.1074/jbc.M610280200

[12] Wang H, Patel V, Miyazaki H, Gutkind JS, Yeudall WA (2009) Role for EPS8 in squamous carcinogenesis. Carcinogenesis 30: 165–174. 19008210 10.1093/carcin/bgn252

[13] Yap LF, Jenei V, Robinson CM, Moutasim K, Benn TM, et al (2009) Upregulation of Eps8 in oral squamous cell carcinoma promotes cell migration and invasion through integrin-dependent Rac1 activation. Oncogene 28: 2524–2534. 19448673 10.1038/onc.2009.105

[14] Matoskova B, Wong WT, Salcini AE, Pelicci PG, Di Fiore PP (1995) Constitutive phosphorylation of eps8 in tumor cell lines: relevance to malignant transformation. Mol Cell Biol 15: 3805–3812. 7791787 10.1128/mcb.15.7.3805 PMC230619

[15] Cunningham DL, Sweet SM, Cooper HJ, Heath JK (2010) Differential phosphoproteomics of fibroblast growth factor signaling: identification of Src family kinase-mediated phosphorylation events. J Proteome Res 9: 2317–2328. 20225815 10.1021/pr9010475 PMC2950672

[16] Blagoev B, Kratchmarova I, Ong SE, Nielsen M, Foster LJ, et al (2003) A proteomics strategy to elucidate functional protein-protein interactions applied to EGF signaling. Nat Biotechnol 21: 315–318. 12577067 10.1038/nbt790

[17] Amanchy R, Kalume DE, Pandey A (2005) Stable isotope labeling with amino acids in cell culture (SILAC) for studying dynamics of protein abundance and posttranslational modifications. Sci STKE 2005: pl2. 15657263 10.1126/stke.2672005pl2

[18] Schulze WX, Mann M (2004) A novel proteomic screen for peptide-protein interactions. J Biol Chem 279: 10756–10764. 14679214 10.1074/jbc.M309909200

[19] Hinsby AM, Olsen JV, Mann M (2004) Tyrosine phosphoproteomics of fibroblast growth factor signaling: a role for insulin receptor substrate-4. J Biol Chem 279: 46438–46447. 15316024 10.1074/jbc.M404537200

[20] Luo Y, Yang C, Jin C, Xie R, Wang F, et al (2009) Novel phosphotyrosine targets of FGFR2IIIb signaling. Cell Signal 21: 1370–1378. 19410646 10.1016/j.cellsig.2009.04.004 PMC2782441

[21] Shevchenko A, Tomas H, Havlis J, Olsen JV, Mann M (2006) In-gel digestion for mass spectrometric characterization of proteins and proteomes. Nat Protoc 1: 2856–2860. 17406544 10.1038/nprot.2006.468

[22] Cox J, Mann M (2008) MaxQuant enables high peptide identification rates, individualized p.p.b.-range mass accuracies and proteome-wide protein quantification. Nat Biotechnol 26: 1367–1372. 19029910 10.1038/nbt.1511

[23] Cox J, Matic I, Hilger M, Nagaraj N, Selbach M, et al (2009) A practical guide to the MaxQuant computational platform for SILAC-based quantitative proteomics. Nat Protoc 4: 698–705. 19373234 10.1038/nprot.2009.36

[24] Kantarjian H, Jabbour E, Grimley J, Kirkpatrick P (2006) Dasatinib. Nat Rev Drug Discov 5: 717–718. 17001803 10.1038/nrd2135

[25] Blake RA, Broome MA, Liu X, Wu J, Gishizky M, et al (2000) SU6656, a selective src family kinase inhibitor, used to probe growth factor signaling. Mol Cell Biol 20: 9018–9027. 11074000 10.1128/mcb.20.23.9018-9027.2000 PMC86555

[26] Mohammadi M, McMahon G, Sun L, Tang C, Hirth P, et al (1997) Structures of the tyrosine kinase domain of fibroblast growth factor receptor in complex with inhibitors. Science 276: 955–960. 9139660 10.1126/science.276.5314.955

[27] de la Fuente van Bentem S, Mentzen WI, de la Fuente A, Hirt H (2008) Towards functional phosphoproteomics by mapping differential phosphorylation events in signaling networks. Proteomics 8: 4453–4465. 18972525 10.1002/pmic.200800175

[28] Hornbeck PV, Chabra I, Kornhauser JM, Skrzypek E, Zhang B (2004) PhosphoSite: A bioinformatics resource dedicated to physiological protein phosphorylation. Proteomics 4: 1551–1561. 15174125 10.1002/pmic.200300772

[29] Qu BH, Karas M, Koval A, LeRoith D (1999) Insulin receptor substrate-4 enhances insulin-like growth factor-I-induced cell proliferation. J Biol Chem 274: 31179–31184. 10531310 10.1074/jbc.274.44.31179

[30] Escribano O, Fernandez-Moreno MD, Zueco JA, Menor C, Fueyo J, et al (2003) Insulin receptor substrate-4 signaling in quiescent rat hepatocytes and in regenerating rat liver. Hepatology 37: 1461–1469. 12774026 10.1053/jhep.2003.50245

[31] Chiba T, Yao J, Higami Y, Shimokawa I, Hosokawa M, et al (2007) Identification of differentially expressed genes in senescence-accelerated mouse testes by suppression subtractive hybridization analysis. Mamm Genome 18: 105–112. 17334656 10.1007/s00335-006-0119-2

[32] Chiba T, Inoue D, Mizuno A, Komatsu T, Fujita S, et al (2009) Identification and characterization of an insulin receptor substrate 4-interacting protein in rat brain: implications for longevity. Neurobiol Aging 30: 474–482. 17720279 10.1016/j.neurobiolaging.2007.07.008

[33] Sano H, Liu SC, Lane WS, Piletz JE, Lienhard GE (2002) Insulin receptor substrate 4 associates with the protein IRAS. J Biol Chem 277: 19439–19447. 11912194 10.1074/jbc.M111838200

[34] Saxton TM, Henkemeyer M, Gasca S, Shen R, Rossi DJ, et al (1997) Abnormal mesoderm patterning in mouse embryos mutant for the SH2 tyrosine phosphatase Shp-2. EMBO J 16: 2352–2364. 9171349 10.1093/emboj/16.9.2352 PMC1169836

[35] Taylor MJ, Perrais D, Merrifield CJ (2011) A high precision survey of the molecular dynamics of mammalian clathrin-mediated endocytosis. PLoS Biol 9: e1000604. 21445324 10.1371/journal.pbio.1000604 PMC3062526

[36] Auciello G, Cunningham DL, Tatar T, Heath JK and Rappoport JZ (2012) Regulation of fibroblast growth factor receptor signalling and trafficking by Src and Eps8. J Cell Sci doi: 10.1242/jcs.116228. 10.1242/jcs.116228 PMC3613183 23203811

[37] Thalappilly S, Soubeyran P, Iovanna JL, Dusetti NJ (2010) VAV2 regulates epidermal growth factor receptor endocytosis and degradation. Oncogene 29: 2528–2539. 20140013 10.1038/onc.2010.1

[38] Mardakheh FK, Auciello G, Dafforn TR, Rappoport JZ, Heath JK (2010) Nbr1 is a novel inhibitor of ligand-mediated receptor tyrosine kinase degradation. Mol Cell Biol 30: 5672–5685. 20937771 10.1128/MCB.00878-10 PMC3004269

[39] Pearce LR, Huang X, Boudeau J, Pawlowski R, Wullschleger S, et al (2007) Identification of Protor as a novel Rictor-binding component of mTOR complex-2. Biochem J 405: 513–522. 17461779 10.1042/BJ20070540 PMC2267312

[40] Jacinto E, Facchinetti V, Liu D, Soto N, Wei S, et al (2006) SIN1/MIP1 maintains rictor-mTOR complex integrity and regulates Akt phosphorylation and substrate specificity. Cell 127: 125–137. 16962653 10.1016/j.cell.2006.08.033

[41] Yang Q, Inoki K, Ikenoue T, Guan KL (2006) Identification of Sin1 as an essential TORC2 component required for complex formation and kinase activity. Genes Dev 20: 2820–2832. 17043309 10.1101/gad.1461206 PMC1619946

[42] Dudka AA, Sweet SM, Heath JK (2010) Signal transducers and activators of transcription-3 binding to the fibroblast growth factor receptor is activated by receptor amplification. Cancer Res 70: 3391–3401. 20388777 10.1158/0008-5472.CAN-09-3033 PMC2887080

[43] DiSorbo D, Shi EG, McKeehan WL (1988) Purification form human hepatoma cells of a 130-kDa membrane glycoprotein with properties of the heparin-binding growth factor receptor. Biochem Biophys Res Commun 157: 1007–1014. 2462864 10.1016/s0006-291x(88)80974-4

[44] Rajalingam K, Wunder C, Brinkmann V, Churin Y, Hekman M, et al (2005) Prohibitin is required for Ras-induced Raf-MEK-ERK activation and epithelial cell migration. Nat Cell Biol 7: 837–843. 16041367 10.1038/ncb1283

[45] Trinkle-Mulcahy L, Boulon S, Lam YW, Urcia R, Boisvert FM, et al (2008) Identifying specific protein interaction partners using quantitative mass spectrometry and bead proteomes. J Cell Biol 183: 223–239. 18936248 10.1083/jcb.200805092 PMC2568020

[46] Boulon S, Ahmad Y, Trinkle-Mulcahy L, Verheggen C, Cobley A, et al (2010) Establishment of a protein frequency library and its application in the reliable identification of specific protein interaction partners. Mol Cell Proteomics 9: 861–879. 20023298 10.1074/mcp.M900517-MCP200 PMC2871420

