# Supplementary figures and images for: Novel Binding Partners and Differentially Regulated Phosphorylation Sites Clarify Eps8 as a Multi-Functional Adaptor
Source: PLoS One. 2013 Apr 23;8(4):e61513. doi: 10.1371/journal.pone.0061513 (PMC3634024; doi:10.1371/journal.pone.0061513)

**A**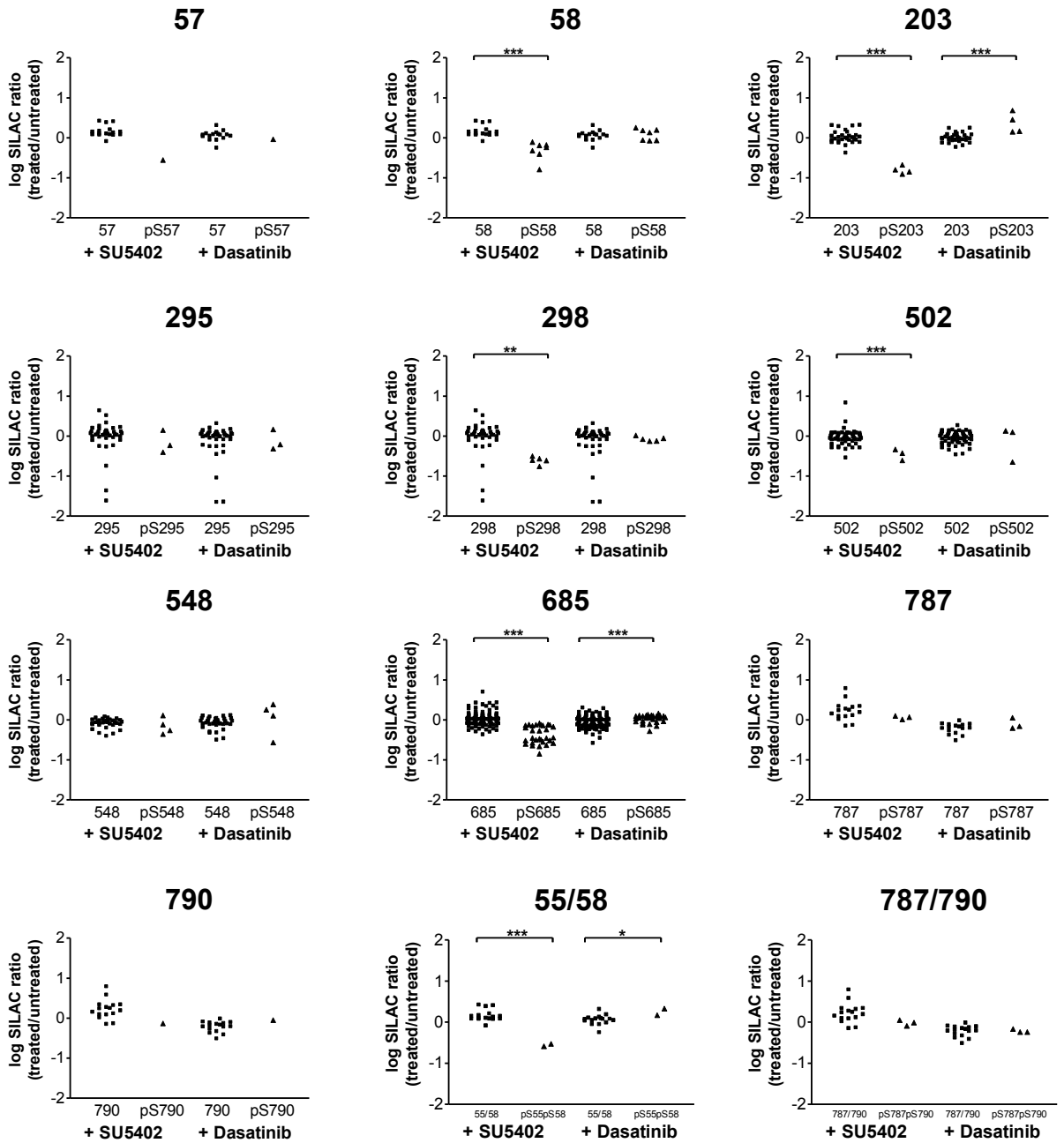

B

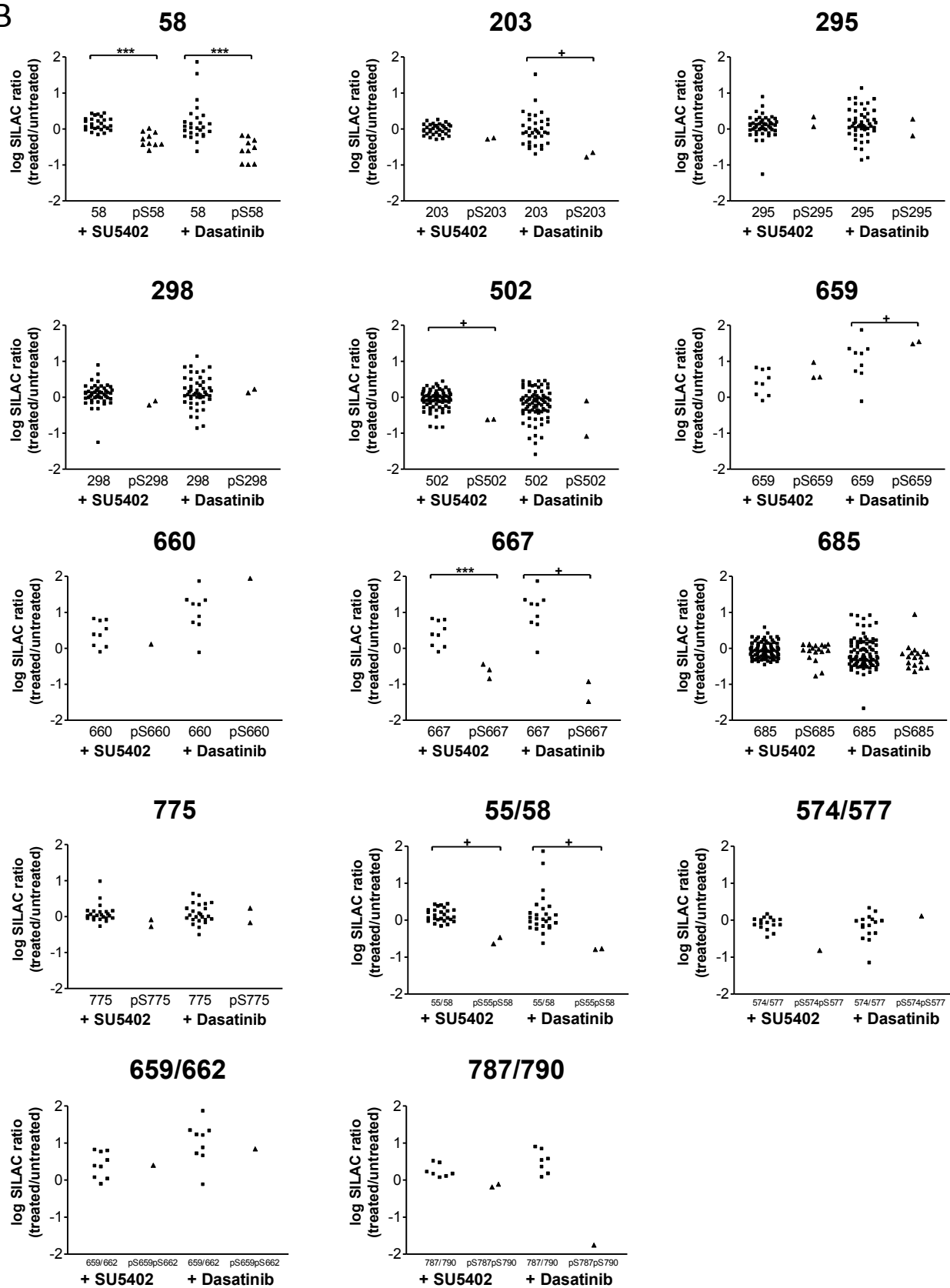

Supplement: Figure S1 — Differential regulation of serine phosphorylation on Eps8. Heavy, medium and light SILAC labelled HEK 293T cells were treated with either dasatinib, SU5402, or no inhibitor, prior to FGF2 stimulation. Myc-Eps8 was immunoprecipitated and the resulting sample was run on an SDS-PAGE gel and, following in-gel trypsin digestion and phophopeptide enrichment, analysed by mass spectrometry. Each graph represents specific residues on Eps8 as indicated. Each data point represents a single peptide identification. P values were calculated by an unpaired t-test (0.01–0.05 = *; 0.001–0.01 = **; <0.001 = ***). +, the median of the ratios is outside the significance cut-off values (<0.57 or >1.75) and is deemed significantly changed (see) A) Experiment was carried out in the absence of sodium pervanadate. B) Experiment was carried out in the presence of 2 mM sodium pervanvadate. (PDF) [file pone.0061513.s001.pdf]

252

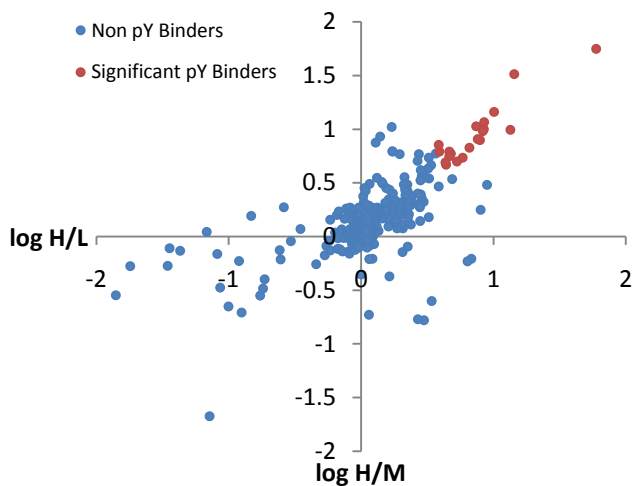

454

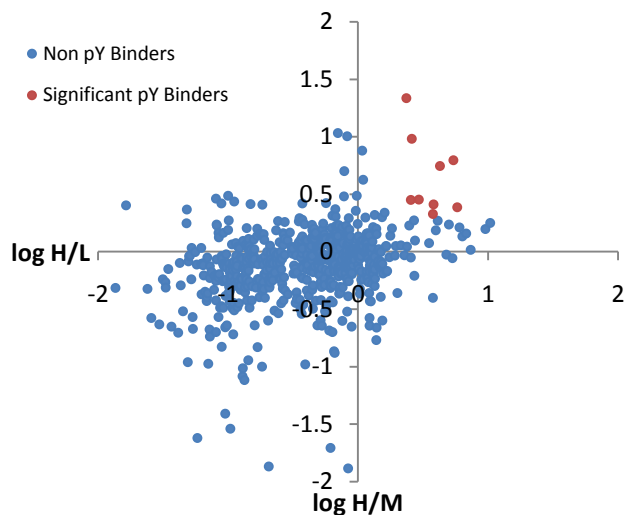

485

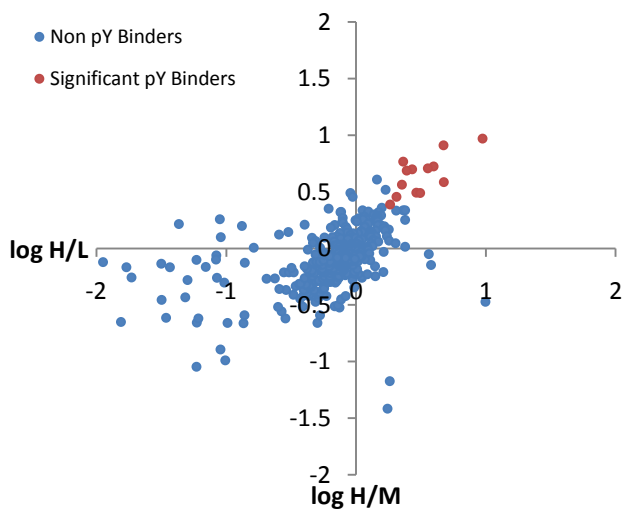

525

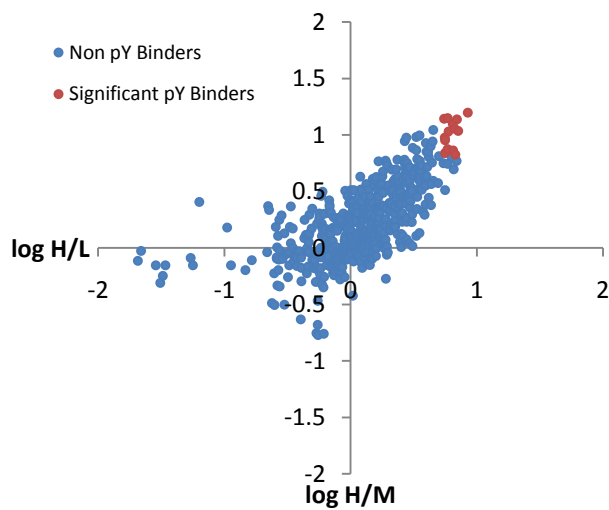

540

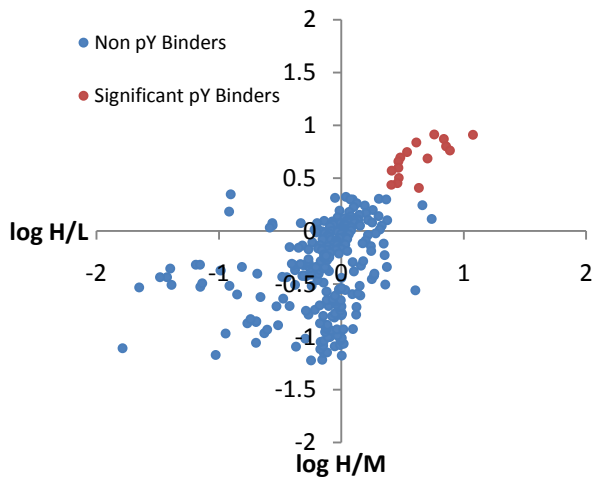

602

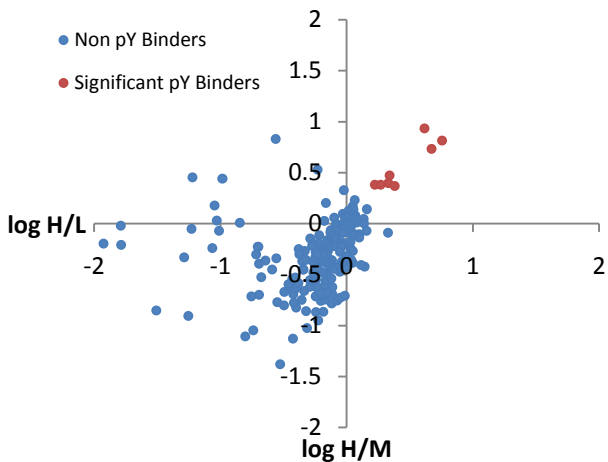

774

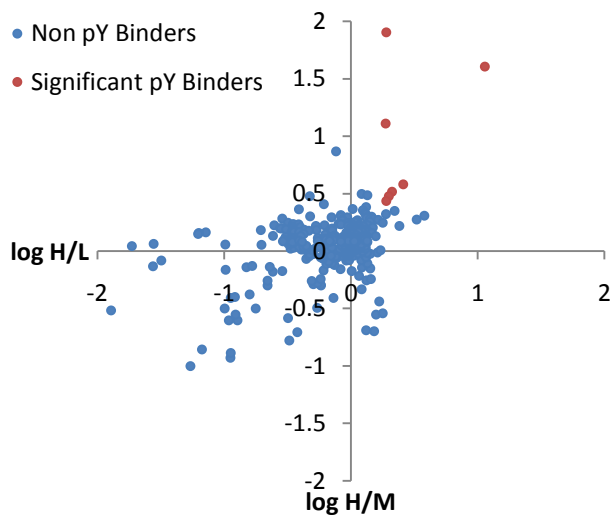

Supplement: Figure S2 — Relative SILAC ratios for proteins identified in peptide pull down assays. Biotinylated Eps8 peptides containing the desired tyrosine residues (the residue number is indicated at the top of each plot) were synthesised in their phosphorylated and non-phosphorylated forms. Three peptide pull down assays for each residue were performed. Using SILAC, we incubated heavy labelled (R10K8) HEK 293T cell lysates with phosphorylated peptide, medium labelled (R6K4) lysates with non-phosphorylated peptide, and light lysates (R0K0) were used as a no peptide control. All cells were treated with FGF2 for 15 min prior to lysis. Following washing, the beads from each assay were combined, the resulting sample run on an SDS-PAGE gel and, following in-gel trypsin digestion, analysed by mass spectrometry. Each graph represents specific residues on Eps8 as indicated. Each data point represents a single protein identification and is shown as a function of its HpY/Mnon-pY and HpY/Lcontrol ratios. Those proteins with both ratios at least 2 standard deviations higher than the median (95% confidence) are shown in red and are considered pY-specific binders (these protein are named in Figure 4). (PDF) [file pone.0061513.s002.pdf]
