# Supplementary material for: Novel Binding Partners and Differentially Regulated Phosphorylation Sites Clarify Eps8 as a Multi-Functional Adaptor
Source: PLoS One. 2013 Apr 23;8(4):e61513. doi: 10.1371/journal.pone.0061513 (PMC3634024; doi:10.1371/journal.pone.0061513)
Supplement: Figure S3 — Additional Mass Spectra of Phosphorylated Peptides Identified. (PDF) [file pone.0061513.s003.pdf]

MATRIX SCIENCE Mascot Search Results

Peptide View

MS/MS Fragmentation of **DSVSSVSDISQYR**
Found in **IPI00290337**, Tax_Id=9606 Gene_Symbol=EPS8 Epidermal growth factor receptor kinase substrate 8

Match to Query 21508: 1521.640994 from(761.827773,2+)
Title: RawFile: CZ_070510_DC_DC4.raw FinneganScanNumber: 3273 _iso_
Data file C:\Users\cunningd\Documents\Mass Spec\Triple SILAC Experiments\070510 Eps8 FGF2 vs Eps8 FGF2 SU5402 vs Eps8 FGF2 Dasatinib + 170510 160610rpts enrich and FT\combined\allSpectra.CID.iso_0.msm

Click mouse within plot area to zoom in by factor of two about that point
Or, Plot from 200 to 1500 Da Full range

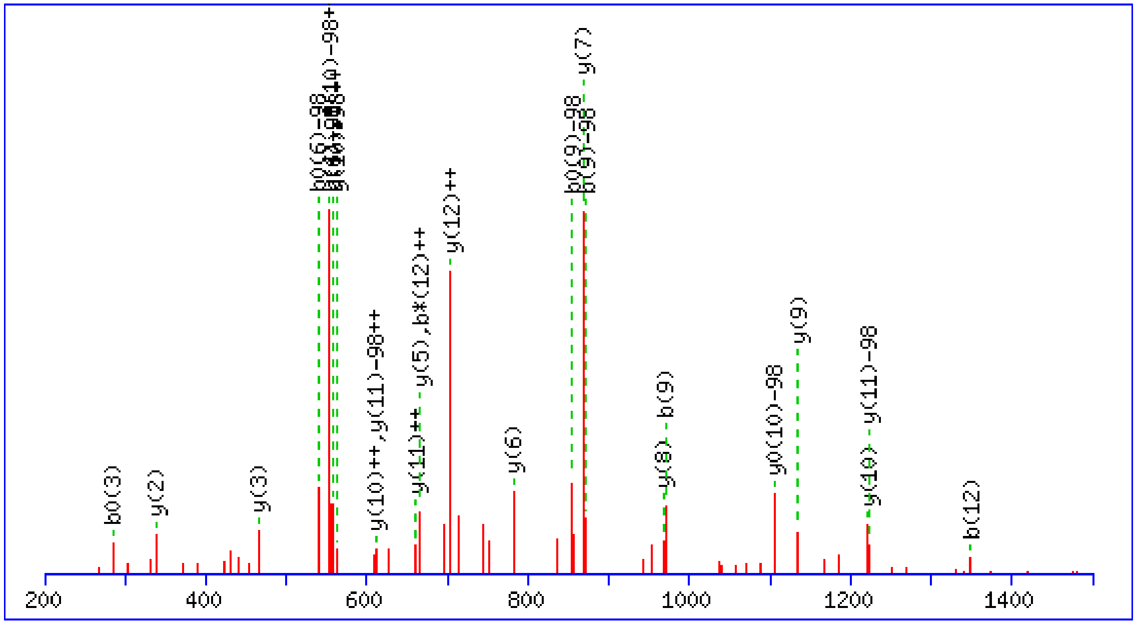

Monoisotopic mass of neutral peptide Mr(calc): 1521.6348
Fixed modifications: Carbamidomethyl (C)
Variable modifications:
S5 : Phospho (ST), with neutral losses 0.0000(shown in table), 97.9769
Ions Score: 63 **Expect:** 0.00031
Matches (Bold Red): 26/178 fragment ions using 30 most intense peaks

#	b	b++	b*	b*++	b0	b0++	Seq.	y	y++	y*	y*++	y0	y0++	#
1	116.0342	58.5207			98.0237	49.5155	D							13
2	203.0662	102.0368			185.0557	93.0315	S	1407.6152	704.3112	1390.5886	695.7979	1389.6046	695.3059	12
3	302.1347	151.5710			284.1241	142.5657	V	1320.5831	660.7952	1303.5566	652.2819	1302.5726	651.7899	11
4	389.1667	195.0870			371.1561	186.0817	S	1221.5147	611.2610	1204.4882	602.7477	1203.5042	602.2557	10
5	556.1650	278.5862			538.1545	269.5809	S	1134.4827	567.7450	1117.4561	559.2317	1116.4721	558.7397	9
6	655.2335	328.1204			637.2229	319.1151	V	967.4843	484.2458	950.4578	475.7325	949.4738	475.2405	8
7	742.2655	371.6364			724.2549	362.6311	S	868.4159	434.7116	851.3894	426.1983	850.4054	425.7063	7
8	857.2924	429.1499			839.2819	420.1446	D	781.3839	391.1956	764.3573	382.6823	763.3733	382.1903	6
9	970.3765	485.6919			952.3659	476.6866	I	666.3570	333.6821	649.3304	325.1688	648.3464	324.6768	5
10	1057.4085	529.2079			1039.3980	520.2026	S	553.2729	277.1401	536.2463	268.6268	535.2623	268.1348	4
11	1185.4671	593.2372	1168.4405	584.7239	1167.4565	584.2319	Q	466.2409	233.6241	449.2143	225.1108			3
12	1348.5304	674.7689	1331.5039	666.2556	1330.5199	665.7636	Y	338.1823	169.5948	321.1557	161.0815			2
13							R	175.1190	88.0631	158.0924	79.5498			1

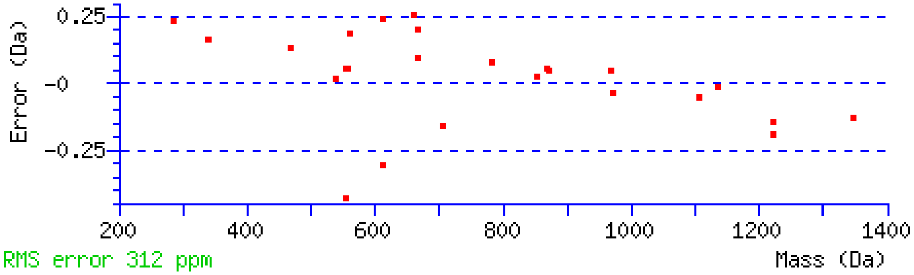

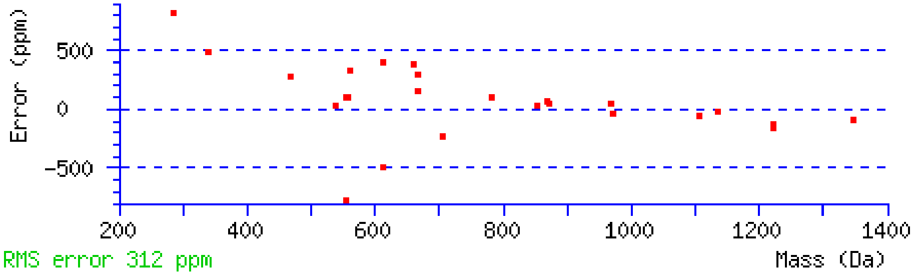

NCBI **BLAST** search of DSVSSVSDISQYR
(Parameters: blastp, nr protein database, expect=20000, no filter, PAM30)
Other BLAST web gateways

All matches to this query

Score	Mr(calc):	Delta	Sequence
62.6	1521.6348	0.0062	DSVSSVSDISQYR
53.1	1521.6348	0.0062	DSVSSVSDISQYR
37.3	1521.6348	0.0062	DSVSSVSDISQYR

37.0	1521.6348	0.0062	DSVSSVSDISQYR
15.7	1521.6348	0.0062	DSVSSVSDISQYR
13.0	1521.6348	0.0062	DSVSSVSDISQYR
8.6	1521.6421	-0.0011	GLSGAGTDSRGRGR
8.6	1521.6421	-0.0011	GLSGAGTDSRGRGR
7.9	1521.6460	-0.0050	EPTNSVSDPNGKR
7.6	1521.6476	-0.0066	XARQMELSSMK

Mascot: http://www.matrixscience.com/

Mascot Search Results

Peptide View

MS/MS Fragmentation of **VYSQITVQK**
Found in **IPI00290337**, Tax_Id=9606 Gene_Symbol=EPS8 Epidermal growth factor receptor kinase substrate 8

Match to Query 13407: 1144.558330 from(573.286441,2+)
Title: RawFile: CZ_070510_DC_EPS8_tio2.raw FinneganScanNumber: 1721 _iso_
Data file C:\Users\cunningd\Documents\Mass Spec\Triple SILAC Experiments\070510 Eps8 FGF2 vs Eps8 FGF2 SU5402 vs Eps8 FGF2 Dasatinib + 170510 160610rpts enrich and FT\combined\allSpectra.CID.iso_0.msm

Click mouse within plot area to zoom in by factor of two about that point

Or, Plot from 200 to 1100 Da Full range

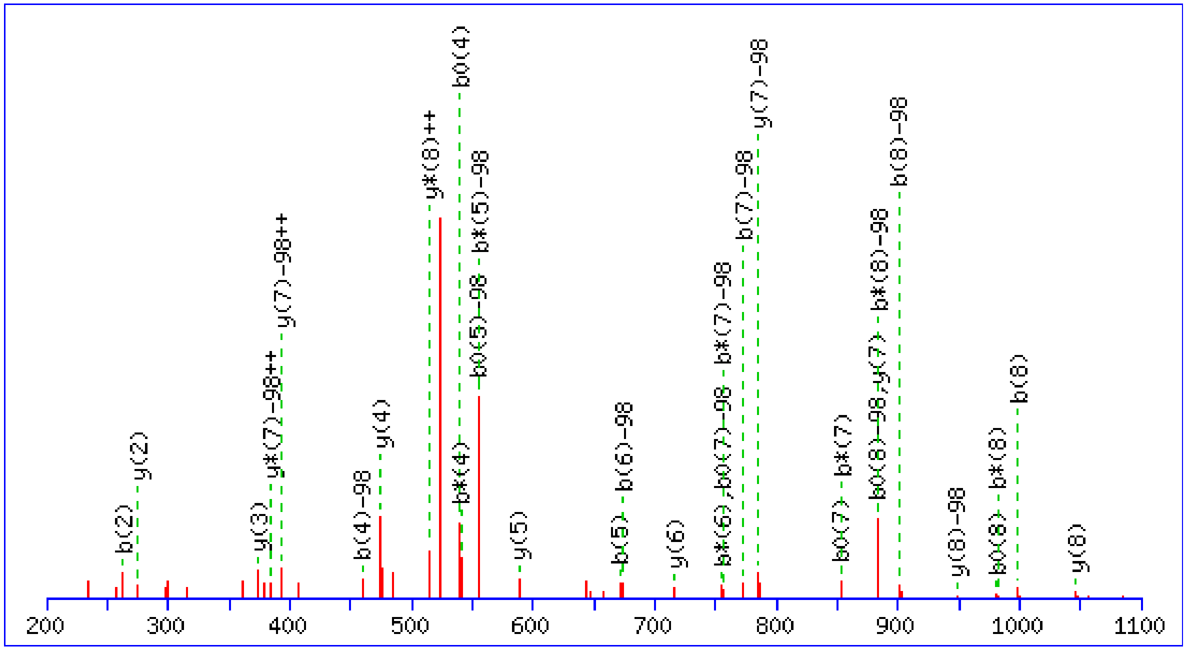

Monoisotopic mass of neutral peptide Mr(calc): 1144.5529
Fixed modifications: Carbamidomethyl (C)
Variable modifications:
S3 : Phospho (ST), with neutral losses 97.9769(shown in table), 0.0000
Ions Score: 39 **Expect:** 0.069

Matches (Bold Red): 32/126 fragment ions using 47 most intense peaks

#	b	b^{++}	b^{*}	b^{*++}	b^{0}	b^{0++}	Seq.	y	y^{++}	y^{*}	y^{*++}	y^{0}	y^{0++}	#
1	100.0757	50.5415					V							9
2	**263.1390**	132.0731					Y	**948.5149**	474.7611	931.4884	466.2478	930.5043	465.7558	8
3	332.1605	166.5839			314.1499	157.5786	S	**785.4516**	**393.2294**	768.4250	**384.7162**	767.4410	384.2241	7
4	**460.2191**	230.6132	443.1925	222.0999	442.2085	221.6079	Q	**716.4301**	358.7187	699.4036	350.2054	698.4196	349.7134	6
5	573.3031	287.1552	**556.2766**	278.6419	**555.2926**	278.1499	I	**588.3715**	294.6894	571.3450	286.1761	570.3610	285.6841	5
6	**674.3508**	337.6790	657.3242	329.1658	656.3402	328.6738	T	**475.2875**	238.1474	458.2609	229.6341	457.2769	229.1421	4
7	**773.4192**	387.2132	**756.3927**	378.7000	**755.4086**	378.2080	V	**374.2398**	187.6235	357.2132	179.1103			3
8	**901.4778**	451.2425	**884.4512**	442.7293	**883.4672**	442.2372	Q	**275.1714**	138.0893	258.1448	129.5761			2
9							K	147.1128	74.0600	130.0863	65.5468			1

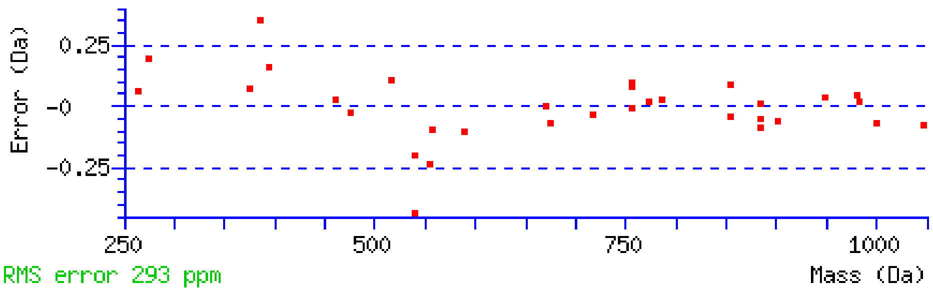

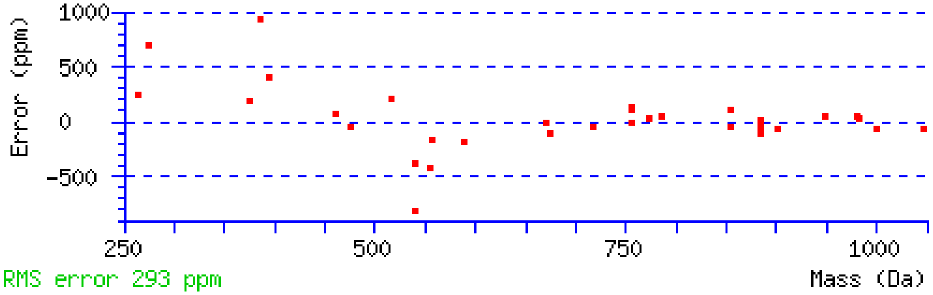

NCBI **BLAST** search of VYSQITVQK
(Parameters: blastp, nr protein database, expect=20000, no filter, PAM30)
Other BLAST web gateways

All matches to this query

Score	Mr(calc):	Delta	Sequence
39.0	1144.5529	0.0054	VYSQITVQK
21.2	1144.5529	0.0054	VYSQITVQK
19.2	1144.5525	0.0058	PEMTTTAKDK
16.5	1144.5529	0.0054	VYSQITVQK

12.6	1144.5587	-0.0003	YQFTMNTIK
9.8	1144.5529	0.0054	AYNTLIKDK
8.9	1144.5529	0.0054	PFSSGKTITK
8.9	1144.5529	0.0054	PFSSGKTITK
8.6	1144.5547	0.0037	VMIDGHDSKK
8.3	1144.5584	-0.0001	RLEVSAEQK

Mascot: http://www.matrixscience.com/

Mascot Search Results

Peptide View

MS/MS Fragmentation of **AAEAFSELSKR**
Found in **IPI00290337**, Tax_Id=9606 Gene_Symbol=EPS8 Epidermal growth factor receptor kinase substrate 8

Match to Query 17746: 1287.594444 from(644.804498,2+)
Title: RawFile: CZ_140510_DC_EPS8_tio2_100517084708.raw FinneganScanNumber: 1586 _iso_
Data file C:\Users\cunningd\Documents\Mass Spec\Triple SILAC Experiments\070510 Eps8 FGF2 vs Eps8 FGF2 SU5402 vs Eps8 FGF2 Dasatinib + 170510 160610rpts enrich and FT\combined\allSpectra.CID.iso_0.msm

Click mouse within plot area to zoom in by factor of two about that point
Or, Plot from 200 to 1300 Da Full range

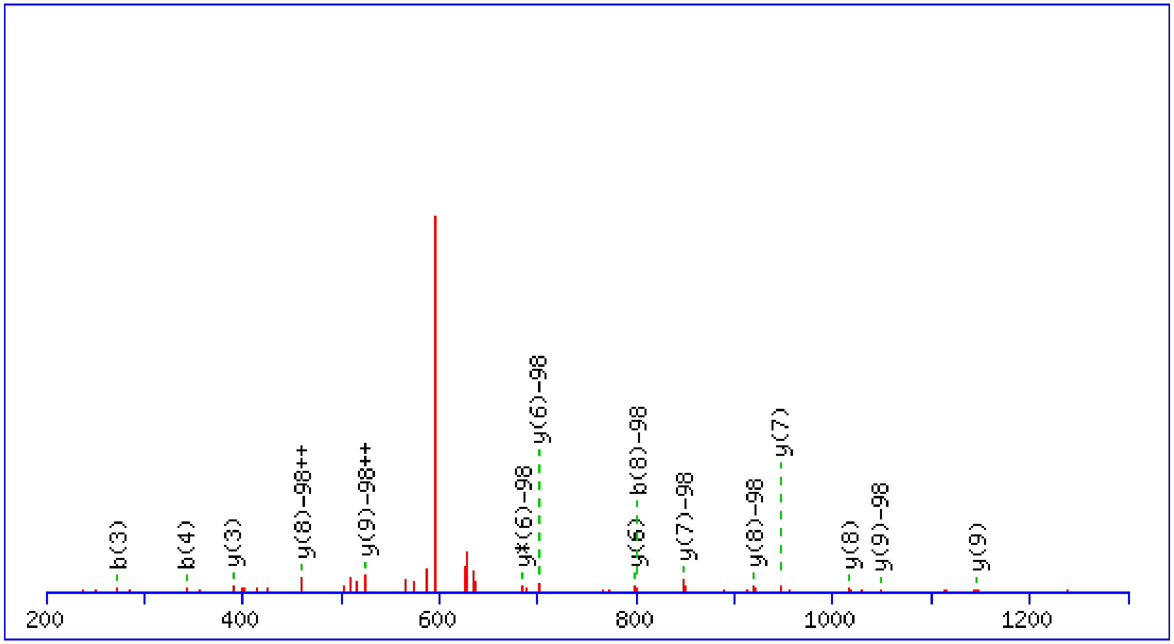

Monoisotopic mass of neutral peptide Mr(calc): 1287.5860
Fixed modifications: Carbamidomethyl (C)
Variable modifications:
S6 : Phospho (ST), with neutral losses 97.9769(shown in table), 0.0000
Ions Score: 35 **Expect:** 0.17

Matches (Bold Red): 15/146 fragment ions using 25 most intense peaks

#	b	b^{++}	b^{*}	b^{*++}	b^{0}	b^{0++}	Seq.	y	y^{++}	y^{*}	y^{*++}	y^{0}	y^{0++}	#
1	72.0444	36.5258					A							11
2	143.0815	72.0444					A	1119.5793	560.2933	1102.5527	551.7800	1101.5687	551.2880	10
3	**272.1241**	136.5657			254.1135	127.5604	E	**1048.5422**	**524.7747**	1031.5156	516.2615	1030.5316	515.7694	9
4	**343.1612**	172.0842			325.1506	163.0790	A	**919.4996**	**460.2534**	902.4730	451.7402	901.4890	451.2481	8
5	490.2296	245.6185			472.2191	236.6132	F	**848.4625**	424.7349	831.4359	416.2216	830.4519	415.7296	7
6	559.2511	280.1292			541.2405	271.1239	S	**701.3941**	351.2007	**684.3675**	342.6874	683.3835	342.1954	6
7	688.2937	344.6505			670.2831	335.6452	E	632.3726	316.6899	615.3461	308.1767	614.3620	307.6847	5
8	**801.3777**	401.1925			783.3672	392.1872	L	503.3300	252.1686	486.3035	243.6554	485.3194	243.1634	4
9	888.4098	444.7085			870.3992	435.7032	S	**390.2459**	195.6266	373.2194	187.1133	372.2354	186.6213	3
10	1016.5047	508.7560	999.4782	500.2427	998.4942	499.7507	K	303.2139	152.1106	286.1874	143.5973			2
11							R	175.1190	88.0631	158.0924	79.5498			1

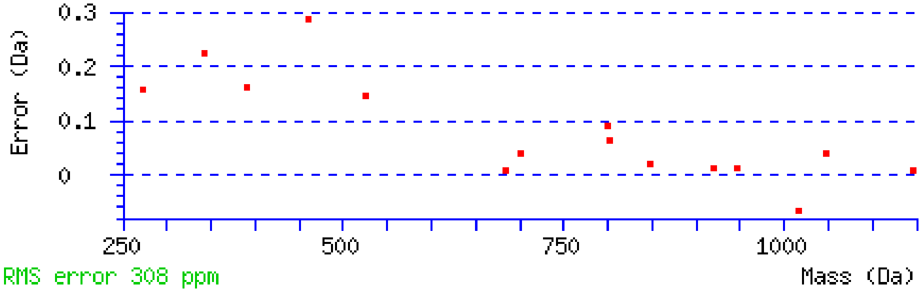

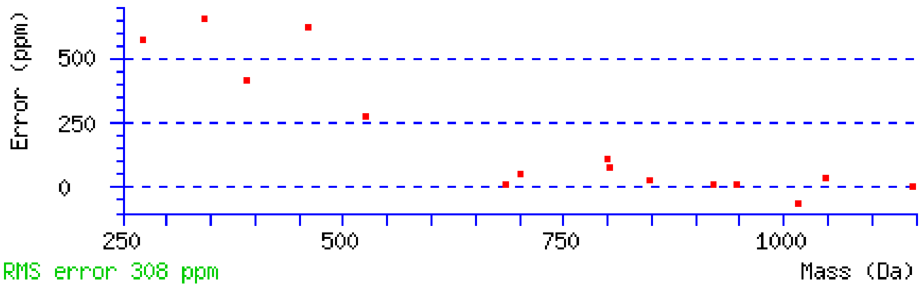

NCBI **BLAST** search of AAEAFSELSKR
(Parameters: blastp, nr protein database, expect=20000, no filter, PAM30)
Other BLAST web gateways

All matches to this query

Score	Mr(calc):	Delta	Sequence
35.2	1287.5860	0.0085	AAEAFSELSKR
24.1	1287.5860	0.0085	AAEAFSELSKR
12.4	1287.5960	-0.0016	DLTRGHVSASR

11.7	1287.6009	-0.0065	RDASEEELKK
11.7	1287.6009	-0.0065	RDASEEELKK
9.8	1287.6030	-0.0085	RWMPTEAPER
9.6	1287.5877	0.0068	HSKEKDAESR
9.6	1287.5922	0.0023	MTQTLKYASR
9.5	1287.5860	0.0084	DQVYDSVKVR
8.8	1287.5872	0.0073	EDFPWSGKVK

Mascot: http://www.matrixscience.com/

Mascot Search Results

Peptide View

MS/MS Fragmentation of **HIDRNYEPLK**
Found in **IPI00290337**, Tax_Id=9606 Gene_Symbol=EPS8 Epidermal growth factor receptor kinase substrate 8

Match to Query 19285: 1363.633908 from(682.824230,2+)
Title: RawFile: CZ_070510_DC_DC6.raw FinneganScanNumber: 1102 _iso_
Data file C:\Users\cunningd\Documents\Mass Spec\Triple SILAC Experiments\070510 Eps8 FGF2 vs Eps8 FGF2 SU5402 vs Eps8 FGF2 Dasatinib + 170510 160610rpts enrich and FT\combined\allSpectra.CID.iso_0.msm

Click mouse within plot area to zoom in by factor of two about that point
Or, Plot from 200 to 1400 Da Full range

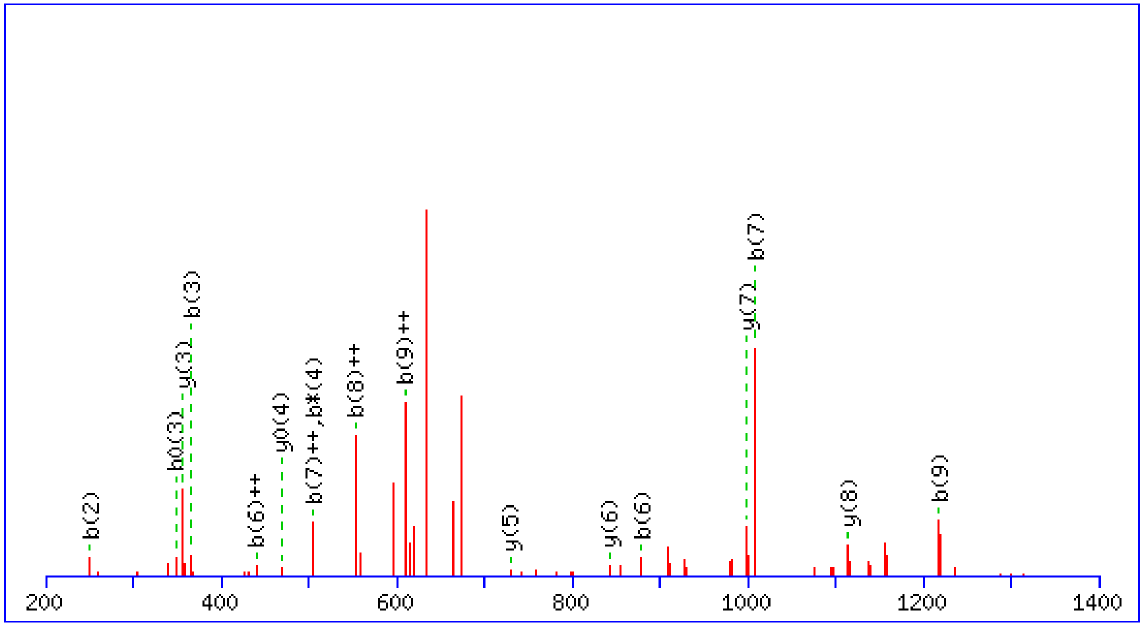

Monoisotopic mass of neutral peptide Mr(calc): 1363.6285
Fixed modifications: Carbamidomethyl (C)
Variable modifications:
Y6 : Phospho (Y)
Ions Score: 27 **Expect:** 1.2
Matches (Bold Red): 17/92 fragment ions using 32 most intense peaks

#	b	b^{++}	b^{*}	b^{*++}	b^{0}	b^{0++}	Seq.	y	y^{++}	y^{*}	y^{*++}	y^{0}	y^{0++}	#
1	138.0662	69.5367					H							10
2	**251.1503**	126.0788					I	1227.5769	614.2921	1210.5504	605.7788	1209.5664	605.2868	9
3	**366.1772**	183.5922			**348.1666**	174.5870	D	**1114.4929**	557.7501	1097.4663	549.2368	1096.4823	548.7448	8
4	522.2783	261.6428	**505.2518**	253.1295	504.2677	252.6375	R	**999.4659**	500.2366	982.4394	491.7233	981.4554	491.2313	7
5	636.3212	318.6643	619.2947	310.1510	618.3107	309.6590	N	**843.3648**	422.1860	826.3383	413.6728	825.3542	413.1808	6
6	**879.3509**	**440.1791**	862.3243	431.6658	861.3403	431.1738	Y	**729.3219**	365.1646	712.2953	356.6513	711.3113	356.1593	5
7	**1008.3935**	**504.7004**	991.3669	496.1871	990.3829	495.6951	E	486.2922	243.6498	469.2657	235.1365	**468.2817**	234.6445	4
8	1105.4462	**553.2268**	1088.4197	544.7135	1087.4357	544.2215	P	**357.2496**	179.1285	340.2231	170.6152			3
9	**1218.5303**	**609.7688**	1201.5038	601.2555	1200.5197	600.7635	L	260.1969	130.6021	243.1703	122.0888			2
10							K	147.1128	74.0600	130.0863	65.5468			1

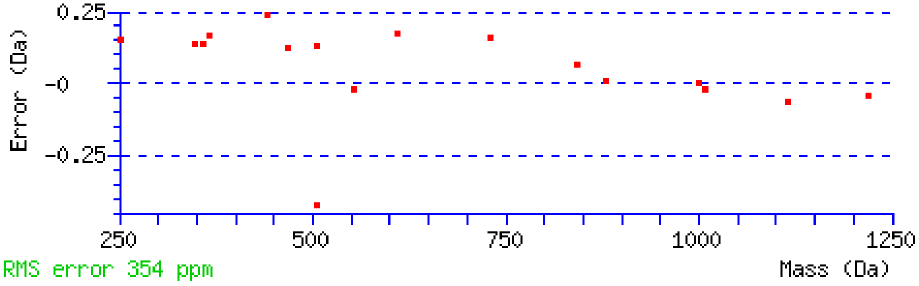

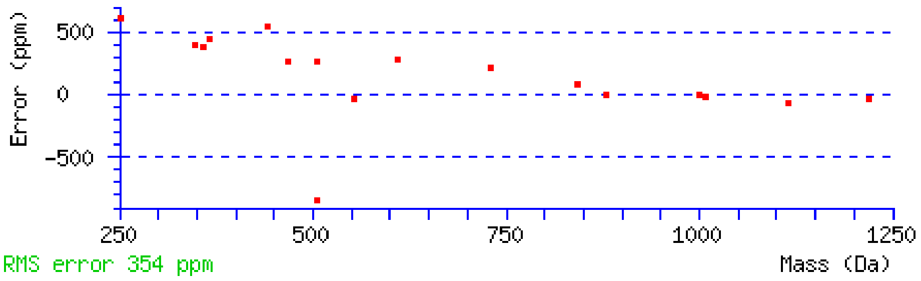

NCBI **BLAST** search of HIDRNYEPLK
(Parameters: blastp, nr protein database, expect=20000, no filter, PAM30)
Other BLAST web gateways

All matches to this query

Score	Mr(calc):	Delta	Sequence
27.4	1363.6285	0.0054	HIDRNYEPLK
11.1	1363.6385	-0.0046	PGSDTIKPDVQK
10.4	1363.6421	-0.0082	QEVEFTITDMK
9.4	1363.6378	-0.0039	QEVKDTVDGQR
9.0	1363.6420	-0.0080	NKGAGRSQEASR
9.0	1363.6290	0.0050	QPSSKGRPPIK

7.8	1363.6341	-0.0002	YSGSKSPGPSRR
7.8	1363.6341	-0.0002	YSGSKSPGPSRR
7.7	1363.6268	0.0071	PEETKRLVSK
7.0	1363.6359	-0.0020	MSRYYVQLPK

Mascot: http://www.matrixscience.com/

{MATRIX SCIENCE} Mascot Search Results

Peptide View

MS/MS Fragmentation of **QNSSSSDSGGSIVR**
Found in **IPI00290337**, Tax_Id=9606 Gene_Symbol=EPS8 Epidermal growth factor receptor kinase substrate 8

Match to Query 20752: 1459.601114 from(730.807833,2+)
Title: RawFile: CZ_140510_DC_EPS8_tio2_100517084708.raw FinneganScanNumber: 849 _iso_
Data file C:\Users\cunningd\Documents\Mass Spec\Triple SILAC Experiments\070510 Eps8 FGF2 vs Eps8 FGF2 SU5402 vs Eps8 FGF2 Dasatinib + 170510 160610rpts enrich and FT\combined\allSpectra.CID.iso_0.msm

Click mouse within plot area to zoom in by factor of two about that point
Or, Plot from 200 to 1500 Da Full range

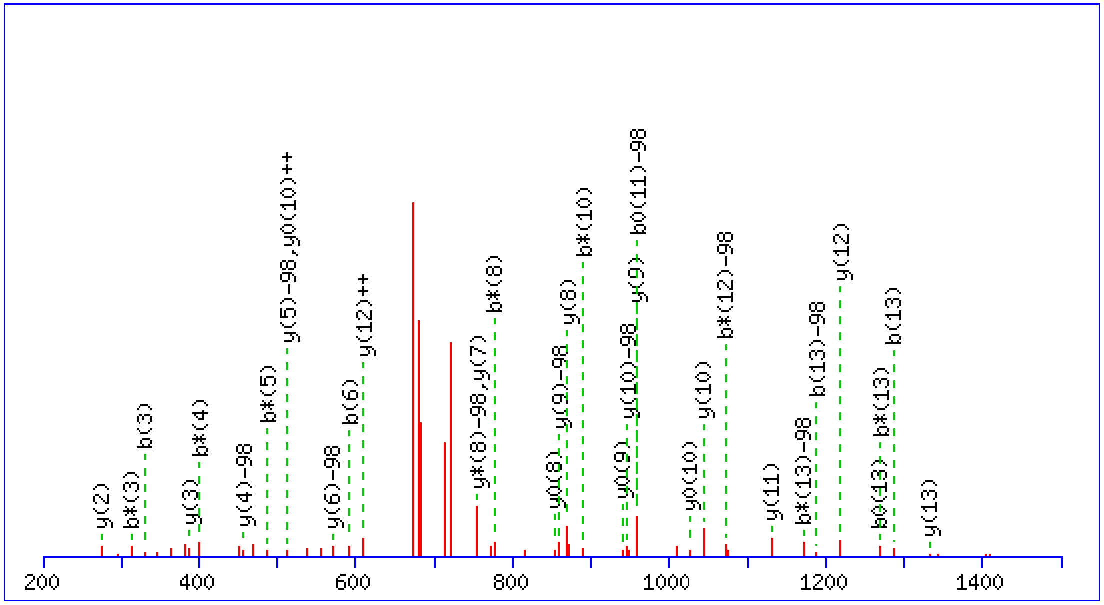

Monoisotopic mass of neutral peptide Mr(calc): 1459.5940
Fixed modifications: Carbamidomethyl (C)
Variable modifications:
S11 : Phospho (ST), with neutral losses 0.0000(shown in table), 97.9769
Ions Score: 41 **Expect:** 0.031
Matches (Bold Red): 34/224 fragment ions using 62 most intense peaks

#	b	b^{++}	b^{*}	b^{*++}	b^{0}	b^{0++}	Seq.	y	y^{++}	y^{*}	y^{*++}	y^{0}	y^{0++}	#
1	129.0659	65.0366	112.0393	56.5233			Q							14
2	243.1088	122.0580	226.0822	113.5448			N	**1332.5427**	666.7750	1315.5162	658.2617	1314.5322	657.7697	13
3	**330.1408**	165.5740	**313.1143**	157.0608	312.1302	156.5688	S	**1218.4998**	**609.7535**	1201.4732	601.2403	1200.4892	600.7483	12
4	417.1728	209.0901	**400.1463**	200.5768	399.1623	200.0848	S	**1131.4678**	566.2375	1114.4412	557.7242	1113.4572	557.2322	11
5	504.2049	252.6061	**487.1783**	244.0928	486.1943	243.6008	S	**1044.4357**	522.7215	1027.4092	514.2082	**1026.4252**	**513.7162**	10
6	**591.2369**	296.1221	574.2103	287.6088	573.2263	287.1168	S	**957.4037**	479.2055	940.3772	470.6922	**939.3931**	470.2002	9
7	706.2638	353.6356	689.2373	345.1223	688.2533	344.6303	D	**870.3717**	435.6895	853.3451	427.1762	**852.3611**	426.6842	8
8	793.2959	397.1516	**776.2693**	388.6383	775.2853	388.1463	S	**755.3447**	378.1760	738.3182	369.6627	737.3342	369.1707	7
9	850.3173	425.6623	833.2908	417.1490	832.3068	416.6570	G	668.3127	334.6600	651.2862	326.1467	650.3021	325.6547	6
10	907.3388	454.1730	**890.3122**	445.6598	889.3282	445.1678	G	611.2912	306.1493	594.2647	297.6360	593.2807	297.1440	5
11	1074.3371	537.6722	1057.3106	529.1589	1056.3266	528.6669	S	554.2698	277.6385	537.2432	269.1253	536.2592	268.6332	4
12	1187.4212	594.2142	1170.3947	585.7010	1169.4106	585.2090	I	**387.2714**	194.1394	370.2449	185.6261			3
13	**1286.4896**	643.7484	**1269.4631**	635.2352	**1268.4791**	634.7432	V	**274.1874**	137.5973	257.1608	129.0840			2
14							R	175.1190	88.0631	158.0924	79.5498			1

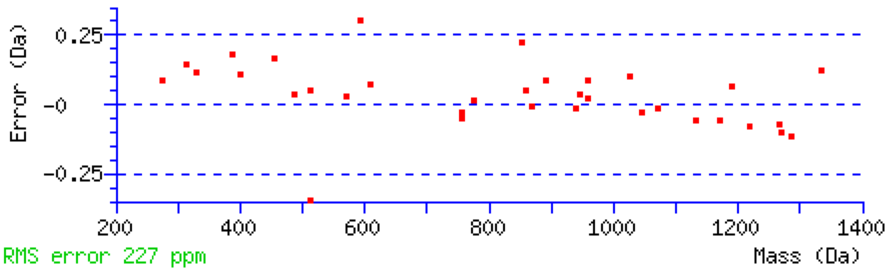

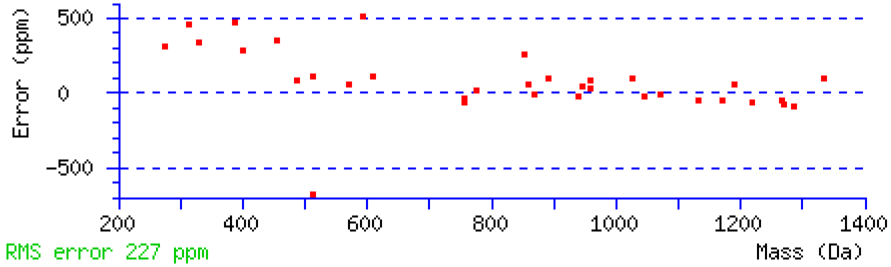

NCBI **BLAST** search of QNSSSSDSGGSIVR
(Parameters: blastp, nr protein database, expect=20000, no filter, PAM30)
Other BLAST web gateways

All matches to this query

Score	Mr(calc):	Delta	Sequence
41.2	1459.5940	0.0071	QNSSSSDSGGSIVR
41.2	1459.5940	0.0071	QNSSSSDSGGSIVR
13.8	1459.5940	0.0071	QNSSSSDSGGSIVR
13.3	1459.5940	0.0071	VISGGSDSSSSNQR

13.3	1459.5940	0.0071	VISGGSDSSSSNQR
13.3	1459.5940	0.0071	VISGGSDSSSSNQR
13.3	1459.5940	0.0071	VISGGSDSSSSNQR
9.9	1459.5928	0.0083	GTKSSGSSSKGSSK
8.5	1459.5940	0.0071	QNSSSSDSGGSIVR
8.0	1459.6064	-0.0053	GGKAVSCEDPSNR

Mascot: http://www.matrixscience.com/

Mascot Search Results

Peptide View

MS/MS Fragmentation of **QNSSSSDSGGSIVR**
Found in **IPI00290337**, Tax_Id=9606 Gene_Symbol=EPS8 Epidermal growth factor receptor kinase substrate 8

Match to Query 20871: 1469.606782 from(735.810667,2+)
Title: RawFile: CZ_070510_DC_DC5.raw FinneganScanNumber: 1475 _iso_
Data file C:\Users\cunningd\Documents\Mass Spec\Triple SILAC Experiments\070510 Eps8 FGF2 vs Eps8 FGF2 SU5402 vs Eps8 FGF2 Dasatinib + 170510 160610rpts enrich and FT\combined\allSpectra.CID.iso_0.msm

Click mouse within plot area to zoom in by factor of two about that point
Or, Plot from 200 to 1500 Da Full range

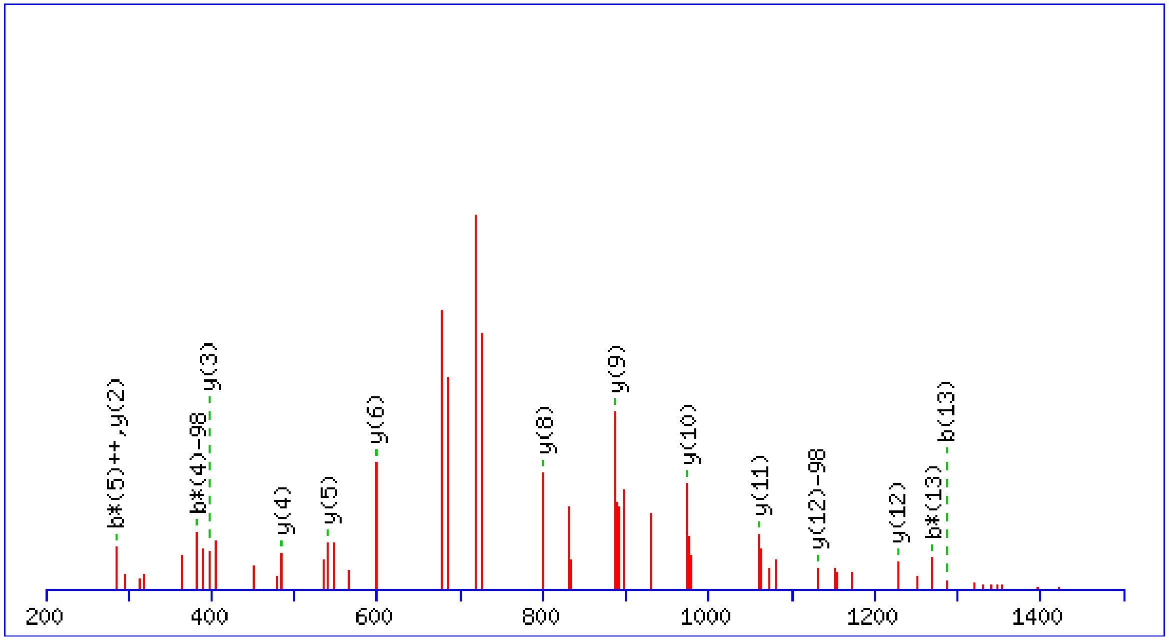

Monoisotopic mass of neutral peptide Mr(calc): 1469.6023
Fixed modifications: Carbamidomethyl (C)
Variable modifications:
S3 : Phospho (ST), with neutral losses 97.9769(shown in table), 0.0000
R14 : Label:13C(6)15N(4) (R)

Ions Score: 50 **Expect:** 0.0042
Matches (Bold Red): 15/224 fragment ions using 41 most intense peaks

#	b	b^{++}	b*	b^{*++}	b^{0}	b^{0++}	Seq.	y	y^{++}	y*	y^{*++}	y^{0}	y^{0++}	#
1	129.0659	65.0366	112.0393	56.5233			Q							14
2	243.1088	122.0580	226.0822	113.5448			N	1244.5741	622.7907	1227.5475	614.2774	1226.5635	613.7854	13
3	312.1302	156.5688	295.1037	148.0555	294.1197	147.5635	S	**1130.5312**	565.7692	1113.5046	557.2559	1112.5206	556.7639	12
4	399.1623	200.0848	**382.1357**	191.5715	381.1517	191.0795	S	**1061.5097**	531.2585	1044.4832	522.7452	1043.4991	522.2532	11
5	486.1943	243.6008	469.1677	235.0875	468.1837	234.5955	S	**974.4777**	487.7425	957.4511	479.2292	956.4671	478.7372	10
6	573.2263	287.1168	556.1998	278.6035	555.2158	278.1115	S	**887.4457**	444.2265	870.4191	435.7132	869.4351	435.2212	9
7	688.2533	344.6303	671.2267	336.1170	670.2427	335.6250	D	**800.4136**	400.7105	783.3871	392.1972	782.4031	391.7052	8
8	775.2853	388.1463	758.2587	379.6330	757.2747	379.1410	S	685.3867	343.1970	668.3601	334.6837	667.3761	334.1917	7
9	832.3068	416.6570	815.2802	408.1437	814.2962	407.6517	G	**598.3547**	299.6810	581.3281	291.1677	580.3441	290.6757	6
10	889.3282	445.1677	872.3017	436.6545	871.3177	436.1625	G	**541.3332**	271.1702	524.3066	262.6570	523.3226	262.1650	5
11	976.3602	488.6838	959.3337	480.1705	958.3497	479.6785	S	**484.3117**	242.6595	467.2852	234.1462	466.3012	233.6542	4
12	1089.4443	545.2258	1072.4178	536.7125	1071.4337	536.2205	I	**397.2797**	199.1435	380.2532	190.6302			3
13	1188.5127	594.7600	1171.4862	586.2467	1170.5022	585.7547	V	**284.1956**	142.6015	267.1691	134.0882			2
14							R	185.1272	93.0672	168.1007	84.5540			1

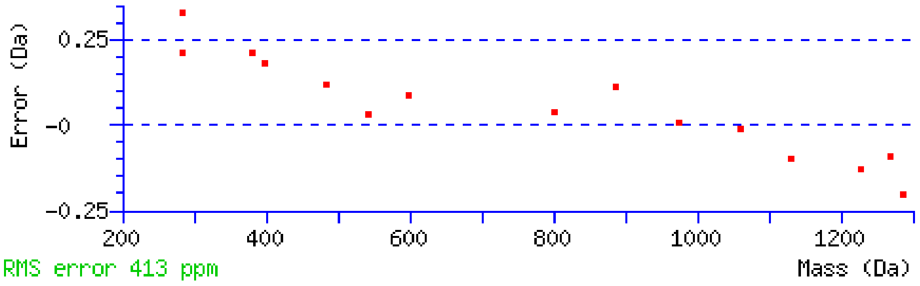

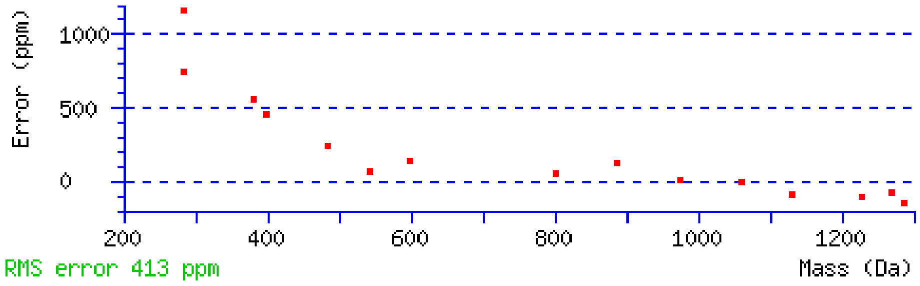

NCBI **BLAST** search of QNSSSSDSGGSIVR
(Parameters: blastp, nr protein database, expect=20000, no filter, PAM30)
Other BLAST web gateways

All matches to this query

Score	Mr(calc):	Delta	Sequence

50.0	1469.6023	0.0045	QNSSSSDSGGSIVR
38.5	1469.6023	0.0045	QNSSSSDSGGSIVR
28.0	1469.6023	0.0045	QNSSSSDSGGSIVR
18.4	1469.6023	0.0045	QNSSSSDSGGSIVR
14.0	1469.5989	0.0079	GGSKYLIYRR
13.8	1469.6040	0.0027	TRGGSTTANNRR
13.8	1469.6040	0.0027	TRGGSTTANNRR
12.2	1469.6023	0.0045	QNSSSSDSGGSIVR
11.8	1469.6053	0.0015	GSNQPGLRTASGR
8.5	1469.6080	-0.0013	MKDSPGSSFTSSK

Mascot: http://www.matrixscience.com/

Mascot Search Results

Peptide View

MS/MS Fragmentation of **SQMEEVQDELIHR**
Found in **IPI00290337**, Tax_Id=9606 Gene_Symbol=EPS8 Epidermal growth factor receptor kinase substrate 8

Match to Query 23458: 1708.719896 from(855.367224,2+)
Title: RawFile: CZ_140510_DC_EPS8_tio2_100517084708.raw FinneganScanNumber: 1651 _iso_
Data file C:\Users\cunningd\Documents\Mass Spec\Triple SILAC Experiments\070510 Eps8 FGF2 vs Eps8 FGF2 SU5402 vs Eps8 FGF2 Dasatinib + 170510 160610rpts enrich and FT\combined\allSpectra.CID.iso_0.msm

Click mouse within plot area to zoom in by factor of two about that point
Or, [Plot from] [200] to [1600] Da [Full range]

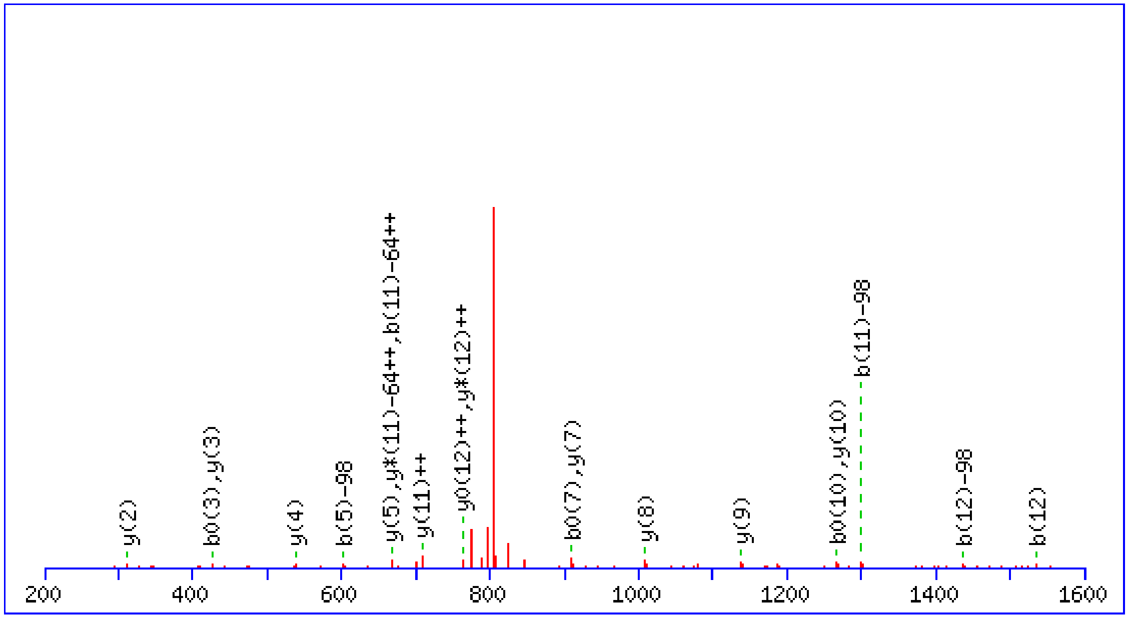

Monoisotopic mass of neutral peptide Mr(calc): 1708.7127
Fixed modifications: Carbamidomethyl (C)
Variable modifications:
S1 : Phospho (ST), with neutral losses 97.9769(shown in table), 0.0000
M3 : Oxidation (M), with neutral losses 0.0000(shown in table), 63.9983
Ions Score: 71 **Expect:** 5e-05
Matches (Bold Red): 20/336 fragment ions using 20 most intense peaks

#	b	b^{++}	b^{*}	b^{*++}	b^{0}	b^{0++}	Seq.	y	y^{++}	y^{*}	y^{*++}	y^{0}	y^{0++}	#
1	70.0287	35.5180			52.0182	26.5127	S							13
2	198.0873	99.5473	181.0608	91.0340	180.0767	90.5420	Q	1542.7217	771.8645	1525.6951	**763.3512**	1524.7111	**762.8592**	12
3	345.1227	173.0650	328.0962	164.5517	327.1121	164.0597	M	1414.6631	**707.8352**	1397.6366	699.3219	1396.6525	698.8299	11
4	474.1653	237.5863	457.1388	229.0730	456.1547	228.5810	E	**1267.6277**	634.3175	1250.6012	625.8042	1249.6171	625.3122	10
5	**603.2079**	302.1076	586.1814	293.5943	585.1973	293.1023	E	**1138.5851**	569.7962	1121.5586	561.2829	1120.5745	560.7909	9
6	702.2763	351.6418	685.2498	343.1285	684.2657	342.6365	V	**1009.5425**	505.2749	992.5160	496.7616	991.5320	496.2696	8
7	830.3349	415.6711	813.3083	407.1578	812.3243	406.6658	Q	**910.4741**	455.7407	893.4476	447.2274	892.4635	446.7354	7
8	945.3618	473.1846	928.3353	464.6713	927.3513	464.1793	D	782.4155	391.7114	765.3890	383.1981	764.4050	382.7061	6
9	1074.4044	537.7059	1057.3779	529.1926	1056.3939	528.7006	E	**667.3886**	334.1979	650.3620	325.6847	649.3780	325.1926	5
10	1187.4885	594.2479	1170.4619	585.7346	1169.4779	585.2426	L	**538.3460**	269.6766	521.3194	261.1634			4
11	**1300.5726**	650.7899	1283.5460	642.2766	1282.5620	641.7846	I	**425.2619**	213.1346	408.2354	204.6213			3
12	**1437.6315**	719.3194	1420.6049	710.8061	1419.6209	710.3141	H	**312.1779**	156.5926	295.1513	148.0793			2
13							R	175.1190	88.0631	158.0924	79.5498			1

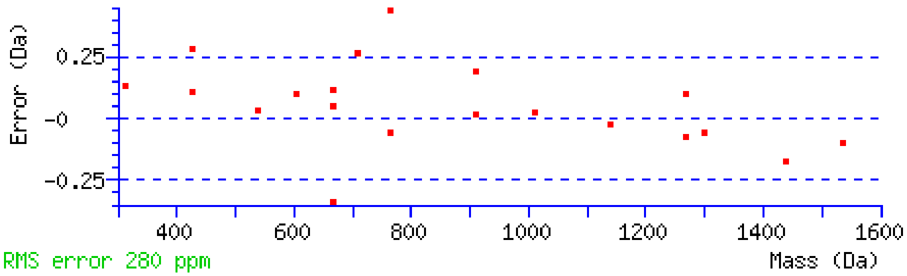

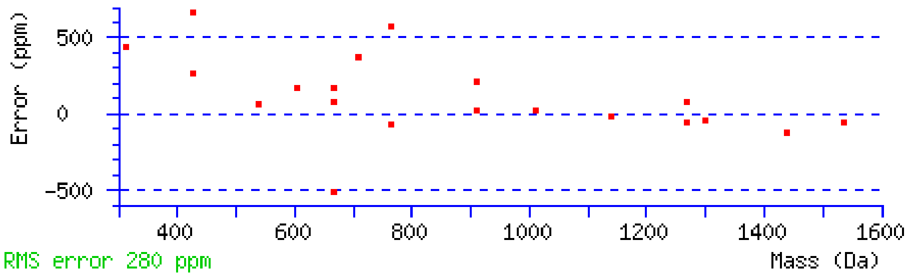

NCBI **BLAST** search of SQMEEVQDELIHR
(Parameters: blastp, nr protein database, expect=20000, no filter, PAM30)
Other BLAST web gateways

All matches to this query

Score	Mr(calc):	Delta	Sequence
71.3	1708.7127	0.0072	SQMEEVQDELIHR
13.7	1708.7158	0.0041	SIERMNXPFSGPGR
13.7	1708.7172	0.0027	EEESKERGEEPHR

12.8	1708.7268	-0.0069	KSNMKTMSAIYQK
10.7	1708.7189	0.0010	SSGAQTKYSNYFDR
10.1	1708.7155	0.0044	SFEAGDLSRSMTYR
9.6	1708.7081	0.0118	ESLDRDDEPGHPPR
9.6	1708.7155	0.0044	SFEAGDLSRSMTYR
9.1	1708.7306	-0.0107	GEGTRGTDGSGAGRWR
8.8	1708.7289	-0.0090	SGSIFSFMHRKTK

Mascot: http://www.matrixscience.com/

Mascot Search Results

Peptide View

MS/MS Fragmentation of **NASGDSGFVPNNILDIVRPPESGLGR**
Found in **IPI00290337**, Tax_Id=9606 Gene_Symbol=EPS8 Epidermal growth factor receptor kinase substrate 8

Match to Query 29755: 2840.299851 from(947.773893,3+)
Title: RawFile: CZ_070510_DC_DC5.raw FinneganScanNumber: 4400 _iso_
Data file C:\Users\cunningd\Documents\Mass Spec\Triple SILAC Experiments\070510 Eps8 FGF2 vs Eps8 FGF2 SU5402 vs Eps8 FGF2 Dasatinib + 170510 160610rpts enrich and FT\combined\allSpectra.CID.iso_0.msm

Click mouse within plot area to zoom in by factor of two about that point
Or, Plot from 300 to 1900 Da Full range

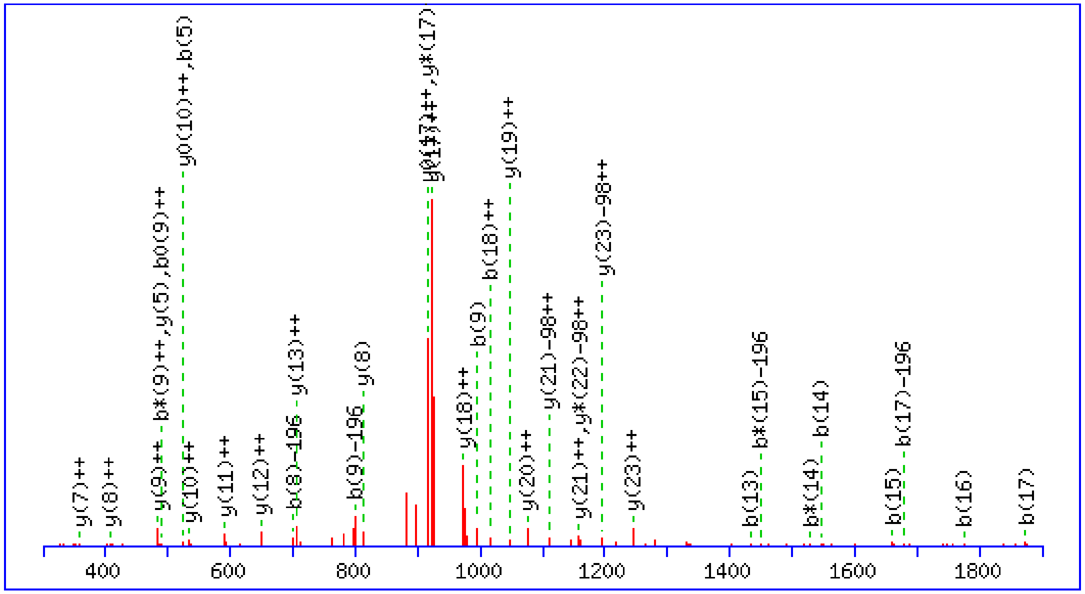

Monoisotopic mass of neutral peptide Mr(calc): 2840.2895
Fixed modifications: Carbamidomethyl (C)
Variable modifications:
S3 : Phospho (ST), with neutral losses 97.9769(shown in table), 0.0000
S6 : Phospho (ST), with neutral losses 97.9769(shown in table), 0.0000
Ions Score: 69 **Expect:** 0.00021
Matches (Bold Red): 36/456 fragment ions using 55 most intense peaks

#	b	b^{++}	b^*	b^{*++}	b^0	b^{0++}	Seq.	y	y^{++}	y^*	y^{*++}	y^0	y^{0++}	#
1	115.0502	58.0287	98.0237	49.5155			N							26
2	186.0873	93.5473	169.0608	85.0340			A	2531.3001	1266.1537	2514.2735	1257.6404	2513.2895	1257.1484	25
3	255.1088	128.0580	238.0822	119.5448	237.0982	119.0527	S	2460.2630	1230.6351	2443.2364	1222.1219	2442.2524	1221.6298	24
4	312.1302	156.5688	295.1037	148.0555	294.1197	147.5635	G	2391.2415	**1196.1244**	2374.2150	1187.6111	2373.2310	1187.1191	23
5	427.1572	214.0822	410.1306	205.5690	409.1466	205.0769	D	2334.2201	1167.6137	2317.1935	**1159.1004**	2316.2095	1158.6084	22
6	496.1786	248.5930	479.1521	240.0797	478.1681	239.5877	S	2219.1931	**1110.1002**	2202.1666	1101.5869	2201.1826	1101.0949	21
7	553.2001	277.1037	536.1736	268.5904	535.1895	268.0984	G	2150.1717	**1075.5895**	2133.1451	1067.0762	2132.1611	1066.5842	20
8	**700.2685**	350.6379	683.2420	342.1246	682.2579	341.6326	F	2093.1502	**1047.0787**	2076.1236	1038.5655	2075.1396	1038.0735	19
9	**799.3369**	400.1721	782.3104	391.6588	781.3264	391.1668	V	1946.0818	**973.5445**	1929.0552	965.0313	1928.0712	964.5392	18
10	896.3897	448.6985	879.3631	440.1852	878.3791	439.6932	P	1847.0134	**924.0103**	1829.9868	**915.4970**	1829.0028	**915.0050**	17
11	1010.4326	505.7199	993.4061	497.2067	992.4221	496.7147	N	1749.9606	875.4839	1732.9341	866.9707	1731.9500	866.4787	16
12	1124.4755	562.7414	1107.4490	554.2281	1106.4650	553.7361	N	1635.9177	818.4625	1618.8911	809.9492	1617.9071	809.4572	15
13	1237.5596	619.2834	1220.5331	610.7702	1219.5490	610.2782	I	1521.8748	761.4410	1504.8482	752.9277	1503.8642	752.4357	14
14	1350.6437	675.8255	1333.6171	667.3122	1332.6331	666.8202	L	1408.7907	**704.8990**	1391.7641	696.3857	1390.7801	695.8937	13
15	1465.6706	733.3389	**1448.6441**	724.8257	1447.6601	724.3337	D	1295.7066	**648.3570**	1278.6801	639.8437	1277.6961	639.3517	12
16	1578.7547	789.8810	1561.7281	781.3677	1560.7441	780.8757	I	1180.6797	**590.8435**	1163.6531	582.3302	1162.6691	581.8382	11
17	**1677.8231**	839.4152	1660.7965	830.9019	1659.8125	830.4099	V	1067.5956	**534.3014**	1050.5691	525.7882	1049.5851	**525.2962**	10
18	1833.9242	917.4657	1816.8977	908.9525	1815.9136	908.4605	R	968.5272	**484.7672**	951.5007	476.2540	950.5166	475.7620	9
19	1930.9770	965.9921	1913.9504	957.4788	1912.9664	956.9868	P	**812.4261**	**406.7167**	795.3995	398.2034	794.4155	397.7114	8
20	2028.0297	1014.5185	2011.0032	1006.0052	2010.0192	1005.5132	P	715.3733	**358.1903**	698.3468	349.6770	697.3628	349.1850	7
21	2157.0723	1079.0398	2140.0458	1070.5265	2139.0618	1070.0345	E	618.3206	309.6639	601.2940	301.1506	600.3100	300.6586	6
22	2244.1044	1122.5558	2227.0778	1114.0425	2226.0938	1113.5505	S	**489.2780**	245.1426	472.2514	236.6293	471.2674	236.1373	5
23	2301.1258	1151.0665	2284.0993	1142.5533	2283.1153	1142.0613	G	402.2459	201.6266	385.2194	193.1133			4
24	2414.2099	1207.6086	2397.1833	1199.0953	2396.1993	1198.6033	L	345.2245	173.1159	328.1979	164.6026			3
25	2471.2313	1236.1193	2454.2048	1227.6060	2453.2208	1227.1140	G	232.1404	116.5738	215.1139	108.0606			2
26							R	175.1190	88.0631	158.0924	79.5498			1

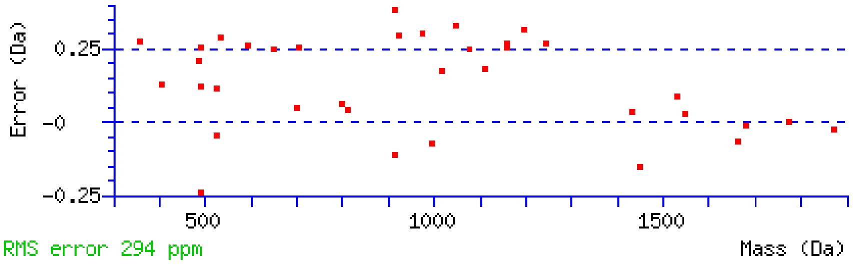

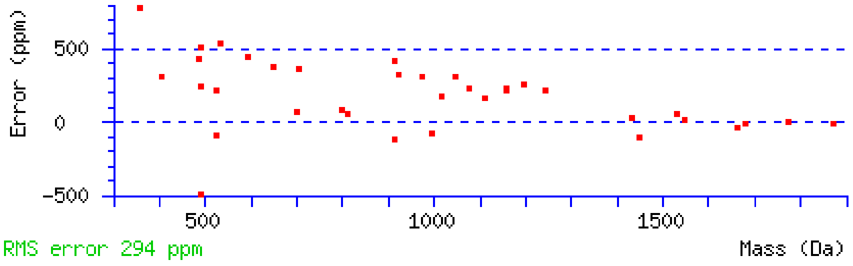

NCBI **BLAST** search of NASGDSGFVPNNILDIVRPPESGLGR
(Parameters: blastp, nr protein database, expect=20000, no filter, PAM30)
Other BLAST web gateways

All matches to this query

Score	Mr(calc):	Delta	Sequence
69.2	2840.2895	0.0103	NASGDSGFVPNNILDIVRPPESGLGR
12.0	2840.2957	0.0041	LRSYCHNMDDLLSLPEEAAPGKGR
8.9	2840.3059	-0.0061	FLQPDGAEHSNVHFRLDEHSLFR
7.2	2840.3051	-0.0053	MDNPIRTFQDLSKQVEMSYGTVR
6.6	2840.2908	0.0091	THSDLKVGEYSNLEVNASWTKER
6.6	2840.3077	-0.0078	LKVAPGPSSGSTPGQVPGSSALSSPRR
6.2	2840.3158	-0.0159	LMSINYLGSVYPSRAVITTMKER
6.2	2840.3158	-0.0159	LMSINYLGSVYPSRAVITTMKER
6.0	2840.3034	-0.0035	ETDFYLQSVERGQRFLAADGDPAR
6.0	2840.3034	-0.0035	ETDFYLQSVERGQRFLAADGDPAR

Mascot: http://www.matrixscience.com/

Mascot Search Results

Peptide View

MS/MS Fragmentation of **SKYDFVAR**
Found in **IPI00290337**, Tax_Id=9606 Gene_Symbol=EPS8 Epidermal growth factor receptor kinase substrate 8

Match to Query 13013: 1064.472100 from(533.243326,2+)
Title: RawFile: CZ_270510_DC_eps8B.raw FinneganScanNumber: 885 _iso_
Data file C:\Users\cunningd\Documents\Mass Spec\Triple SILAC Experiments\210510+270510 rpts Eps8 FGF2 vs Eps8 FGF2 SU5402 vs Eps8 FGF2 Dasatinib\B\combined\allSpectra.CID.iso_0.msm

Click mouse within plot area to zoom in by factor of two about that point
Or, Plot from 200 to 1000 Da Full range

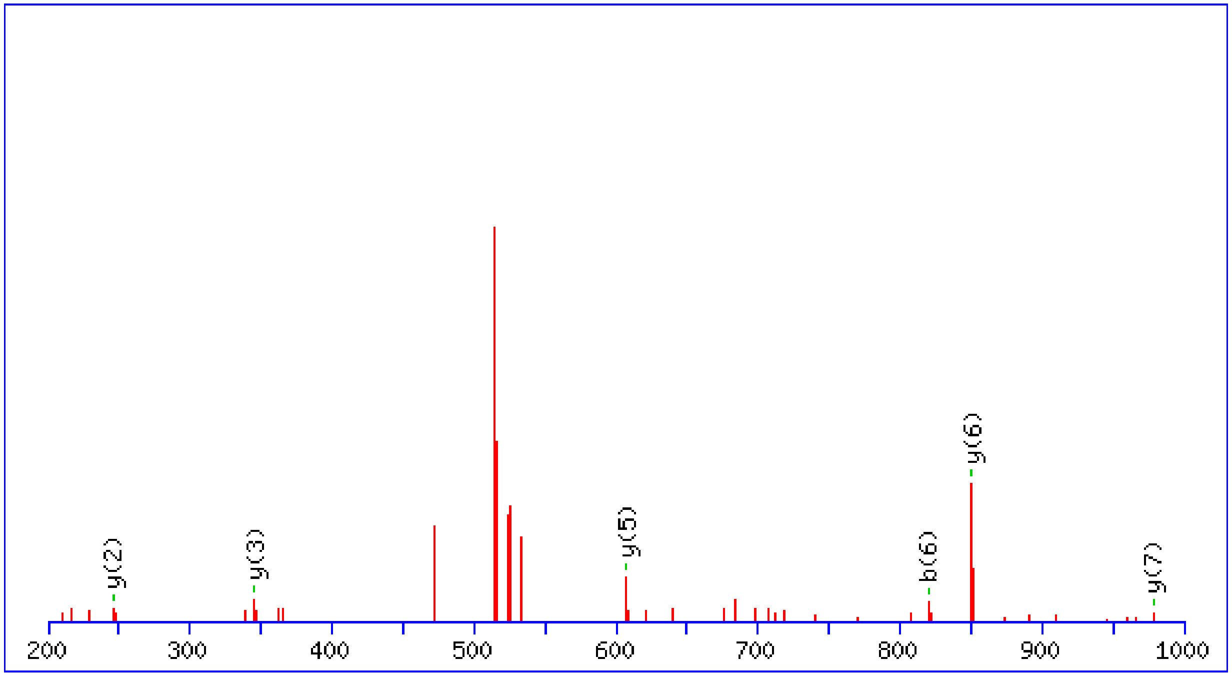

Monoisotopic mass of neutral peptide Mr(calc): 1064.4692
Fixed modifications: Carbamidomethyl (C)
Variable modifications:

Y3 : Phospho (Y)
Ions Score: 23 **Expect:** 1.8
Matches (Bold Red): 6/74 fragment ions using 18 most intense peaks

#	b	b^{++}	b^{*}	b^{*++}	b^{0}	b^{0++}	Seq.	y	y^{++}	y^{*}	y^{*++}	y^{0}	y^{0++}	#
1	88.0393	44.5233			70.0287	35.5180	S							8
2	216.1343	108.5708	199.1077	100.0575	198.1237	99.5655	K	**978.4445**	489.7259	961.4179	481.2126	960.4339	480.7206	7
3	459.1639	230.0856	442.1374	221.5723	441.1534	221.0803	Y	**850.3495**	425.6784	833.3229	417.1651	832.3389	416.6731	6
4	574.1909	287.5991	557.1643	279.0858	556.1803	278.5938	D	**607.3198**	304.1636	590.2933	295.6503	589.3093	295.1583	5
5	721.2593	361.1333	704.2327	352.6200	703.2487	352.1280	F	492.2929	246.6501	475.2663	238.1368			4
6	**820.3277**	410.6675	803.3011	402.1542	802.3171	401.6622	V	**345.2245**	173.1159	328.1979	164.6026			3
7	891.3648	446.1860	874.3383	437.6728	873.3542	437.1808	A	**246.1561**	123.5817	229.1295	115.0684			2
8							R	175.1190	88.0631	158.0924	79.5498			1

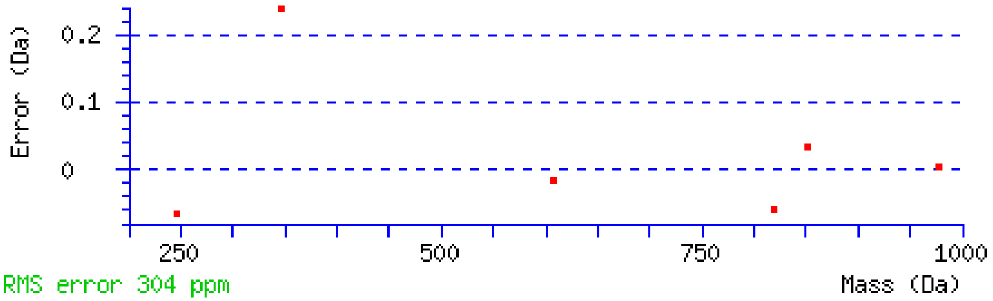

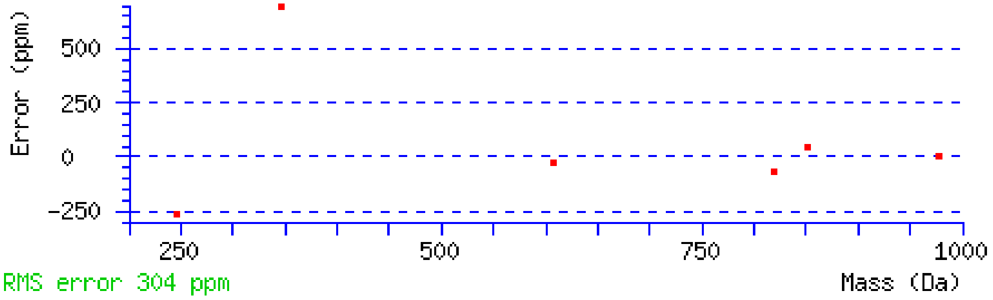

NCBI **BLAST** search of SKYDFVAR
(Parameters: blastp, nr protein database, expect=20000, no filter, PAM30)
Other BLAST web gateways

All matches to this query

Score	Mr(calc):	Delta	Sequence
22.6	1064.4692	0.0029	SKYDFVAR
12.9	1064.4764	-0.0043	NTGTTKHAR
11.9	1064.4743	-0.0022	XQMLPCSAR
11.8	1064.4743	-0.0022	SKSDGEAKR
11.8	1064.4781	-0.0060	SKSKEMNR
11.7	1064.4652	0.0069	SSGVQPSPAR
11.5	1064.4764	-0.0043	NTGTTKHAR
10.8	1064.4707	0.0014	SQRGSTSTR
10.2	1064.4692	0.0029	SKYDFVAR
9.0	1064.4713	0.0008	SKLPAGSCR

Mascot: http://www.matrixscience.com/

{MATRIX SCIENCE} Mascot Search Results

Peptide View

MS/MS Fragmentation of **LSTEHSSVSEYHPADGYAFSSNIYTR**
Found in **IPI00290337**, Tax_Id=9606 Gene_Symbol=EPS8 Epidermal growth factor receptor kinase substrate 8

Match to Query 43306: 2997.282267 from(1000.101365,3+)
Title: RawFile: CZ_270510_DC_B3.raw FinneganScanNumber: 2167 _iso_
Data file C:\Users\cunningd\Documents\Mass Spec\Triple SILAC Experiments\210510+270510 rpts Eps8 FGF2 vs Eps8 FGF2 SU5402 vs Eps8 FGF2 Dasatinib\B\combined\allSpectra.CID.iso_0.msm

Click mouse within plot area to zoom in by factor of two about that point
Or, Plot from 300 to 2000 Da Full range

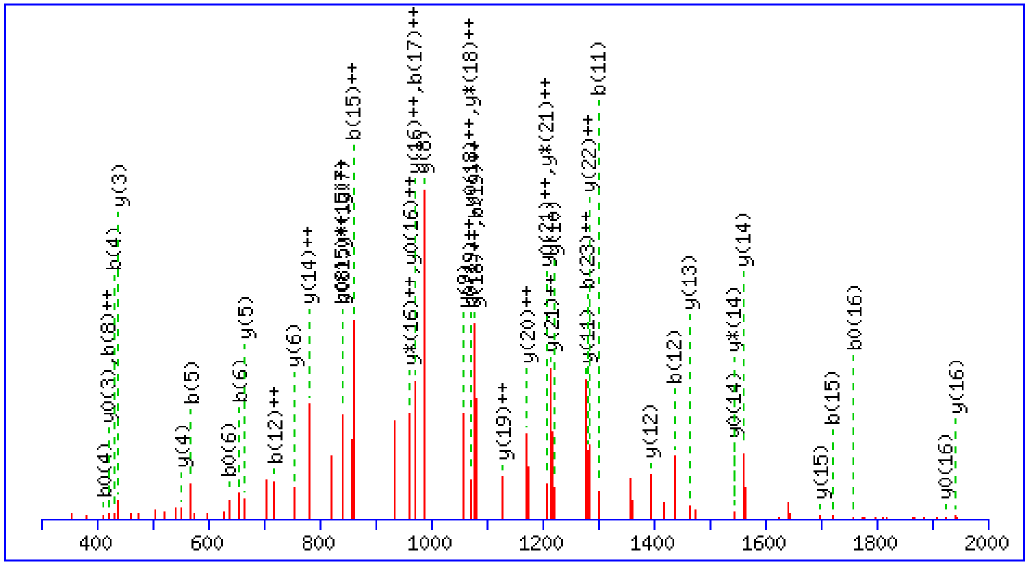

Monoisotopic mass of neutral peptide Mr(calc): 2997.2818
Fixed modifications: Carbamidomethyl (C)
Variable modifications:
Y11 : Phospho (Y)
Ions Score: 62 **Expect:** 0.001
Matches (Bold Red): 50/254 fragment ions using 92 most intense peaks

#	b	b++	b*	b*++	b0	b0++	Seq.	y	y++	y*	y*++	y0	y0++	#
1	114.0913	57.5493					L							26
2	201.1234	101.0653			183.1128	92.0600	S	2885.2050	1443.1061	2868.1785	1434.5929	2867.1945	1434.1009	25

3	302.1710	151.5892			284.1605	142.5839	T	2798.1730	1399.5901	2781.1464	1391.0769	2780.1624	1390.5848	24
4	431.2136	216.1105			413.2031	207.1052	E	2697.1253	1349.0663	2680.0988	1340.5530	2679.1147	1340.0610	23
5	568.2726	284.6399			550.2620	275.6346	H	2568.0827	1284.5450	2551.0562	1276.0317	2550.0722	1275.5397	22
6	655.3046	328.1559			637.2940	319.1506	S	2431.0238	1216.0155	2413.9973	1207.5023	2413.0132	1207.0103	21
7	742.3366	371.6719			724.3260	362.6667	S	2343.9918	1172.4995	2326.9652	1163.9863	2325.9812	1163.4942	20
8	841.4050	421.2061			823.3945	412.2009	V	2256.9597	1128.9835	2239.9332	1120.4702	2238.9492	1119.9782	19
9	928.4370	464.7222			910.4265	455.7169	S	2157.8913	1079.4493	2140.8648	1070.9360	2139.8808	1070.4440	18
10	1057.4796	529.2435			1039.4691	520.2382	E	2070.8593	1035.9333	2053.8328	1027.4200	2052.8487	1026.9280	17
11	1300.5093	650.7583			1282.4987	641.7530	Y	1941.8167	971.4120	1924.7902	962.8987	1923.8061	962.4067	16
12	1437.5682	719.2877			1419.5576	710.2825	H	1698.7871	849.8972	1681.7605	841.3839	1680.7765	840.8919	15
13	1534.6210	767.8141			1516.6104	758.8088	P	1561.7281	781.3677	1544.7016	772.8544	1543.7176	772.3624	14
14	1605.6581	803.3327			1587.6475	794.3274	A	1464.6754	732.8413	1447.6488	724.3281	1446.6648	723.8360	13
15	1720.6850	860.8462			1702.6745	851.8409	D	1393.6383	697.3228	1376.6117	688.8095	1375.6277	688.3175	12
16	1777.7065	889.3569			1759.6959	880.3516	G	1278.6113	639.8093	1261.5848	631.2960	1260.6008	630.8040	11
17	1940.7698	970.8885			1922.7593	961.8833	Y	1221.5899	611.2986	1204.5633	602.7853	1203.5793	602.2933	10
18	2011.8069	1006.4071			1993.7964	997.4018	A	1058.5265	529.7669	1041.5000	521.2536	1040.5160	520.7616	9
19	2158.8753	1079.9413			2140.8648	1070.9360	F	987.4894	494.2483	970.4629	485.7351	969.4789	485.2431	8
20	2245.9074	1123.4573			2227.8968	1114.4520	S	840.4210	420.7141	823.3945	412.2009	822.4104	411.7089	7
21	2332.9394	1166.9733			2314.9288	1157.9681	S	753.3890	377.1981	736.3624	368.6849	735.3784	368.1928	6
22	2446.9823	1223.9948	2429.9558	1215.4815	2428.9718	1214.9895	N	666.3570	333.6821	649.3304	325.1688	648.3464	324.6768	5
23	2560.0664	1280.5368	2543.0398	1272.0236	2542.0558	1271.5316	I	552.3140	276.6607	535.2875	268.1474	534.3035	267.6554	4
24	2723.1297	1362.0685	2706.1032	1353.5552	2705.1192	1353.0632	Y	439.2300	220.1186	422.2034	211.6053	421.2194	211.1133	3
25	2824.1774	1412.5923	2807.1509	1404.0791	2806.1668	1403.5871	T	276.1666	138.5870	259.1401	130.0737	258.1561	129.5817	2
26							R	175.1190	88.0631	158.0924	79.5498			1

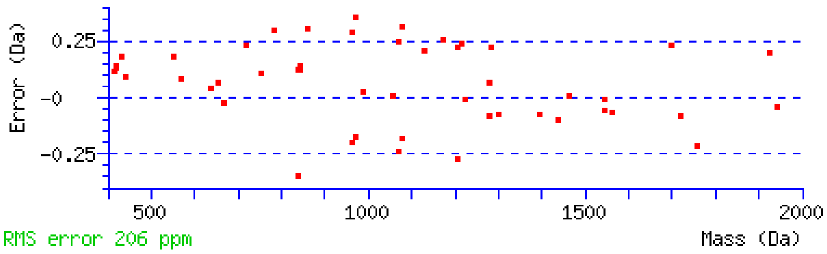

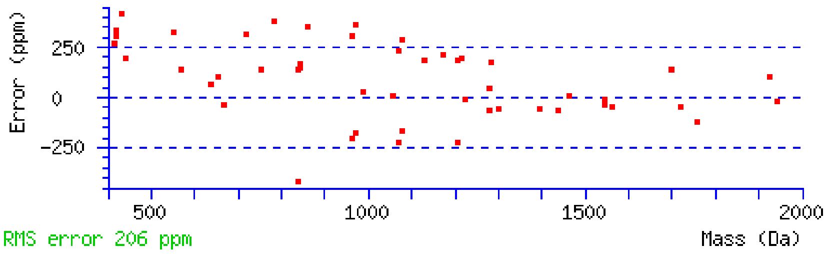

NCBI **BLAST** search of LSTEHSSVSEYHPADGYAFSSNIYTR
(Parameters: blastp, nr protein database, expect=20000, no filter, PAM30)
Other BLAST web gateways

All matches to this query

Score	Mr(calc):	Delta	Sequence
62.1	2997.2818	0.0005	LSTEHSSVSEYHPADGYAFSSNIYTR
53.5	2997.2818	0.0005	LSTEHSSVSEYHPADGYAFSSNIYTR
50.1	2997.2818	0.0005	LSTEHSSVSEYHPADGYAFSSNIYTR
46.8	2997.2818	0.0005	LSTEHSSVSEYHPADGYAFSSNIYTR
39.3	2997.2818	0.0005	LSTEHSSVSEYHPADGYAFSSNIYTR
39.0	2997.2818	0.0005	LSTEHSSVSEYHPADGYAFSSNIYTR
38.9	2997.2818	0.0005	LSTEHSSVSEYHPADGYAFSSNIYTR
32.1	2997.2818	0.0005	LSTEHSSVSEYHPADGYAFSSNIYTR
27.3	2997.2818	0.0005	LSTEHSSVSEYHPADGYAFSSNIYTR
13.7	2997.2818	0.0005	LSTEHSSVSEYHPADGYAFSSNIYTR

Mascot: http://www.matrixscience.com/

Mascot Search Results

Peptide View

MS/MS Fragmentation of **NNSELSVLK**
Found in **IPI00290337**, Tax_Id=9606 Gene_Symbol=EPS8 Epidermal growth factor receptor kinase substrate 8

Match to Query 14090: 1082.503130 from(542.258841,2+)
Title: RawFile: CZ_270810_eps81b.raw FinneganScanNumber: 1828 _iso_
Data file C:\Users\cunningd\Documents\Mass Spec\Triple SILAC Experiments\270810 NO NaP Eps8 FGF2 vs Eps8 FGF2SU5402 vs Eps8 FGF2 Dasatinib\Expt 1 and expt 2\combined\allSpectra.CID.iso_0.msm

Click mouse within plot area to zoom in by factor of two about that point
Or, Plot from 200 to 1050 Da Full range

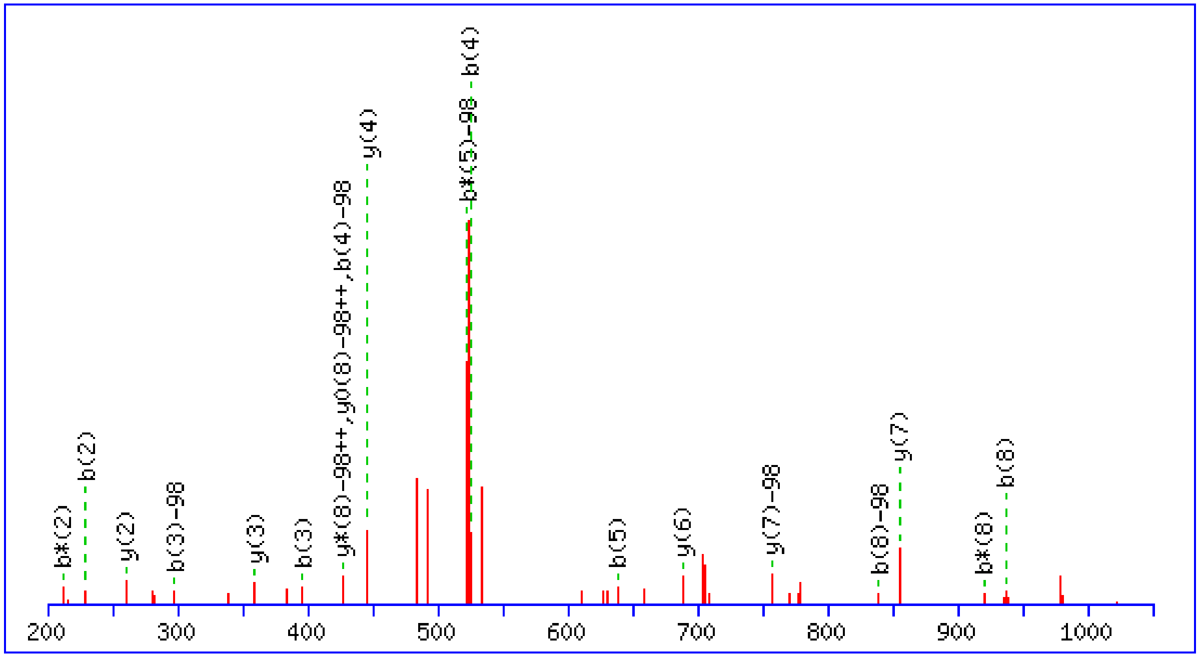

Monoisotopic mass of neutral peptide Mr(calc): 1082.5009
Fixed modifications: Carbamidomethyl (C)
Variable modifications:
S3 : Phospho (ST), with neutral losses 97.9769(shown in table), 0.0000

Ions Score: 31 **Expect:** 0.3
Matches (Bold Red): 19/134 fragment ions using 32 most intense peaks

#	b	b++	b*	b*++	b0	b0++	Seq.	y	y++	y*	y*++	y0	y0++	#
1	115.0502	58.0287	98.0237	49.5155			N							9
2	**229.0931**	115.0502	**212.0666**	106.5369			N	871.4883	436.2478	854.4618	**427.7345**	853.4778	**427.2425**	8
3	**298.1146**	149.5609	281.0880	141.0477	280.1040	140.5556	S	**757.4454**	379.2263	740.4189	370.7131	739.4349	370.2211	7
4	**427.1572**	214.0822	410.1306	205.5690	409.1466	205.0769	E	**688.4240**	344.7156	671.3974	336.2023	670.4134	335.7103	6
5	540.2412	270.6243	**523.2147**	262.1110	522.2307	261.6190	L	559.3814	280.1943	542.3548	271.6811	541.3708	271.1890	5
6	627.2733	314.1403	610.2467	305.6270	609.2627	305.1350	S	**446.2973**	223.6523	429.2708	215.1390	428.2867	214.6470	4
7	726.3417	363.6745	709.3151	355.1612	708.3311	354.6692	V	**359.2653**	180.1363	342.2387	171.6230			3
8	**839.4257**	420.2165	822.3992	411.7032	821.4152	411.2112	L	**260.1969**	130.6021	243.1703	122.0888			2
9							K	147.1128	74.0600	130.0863	65.5468			1

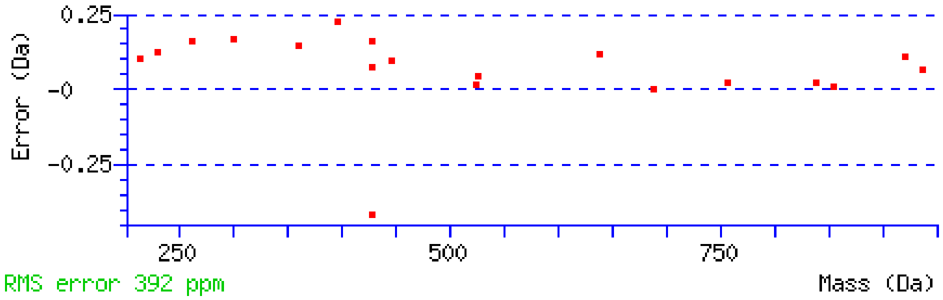

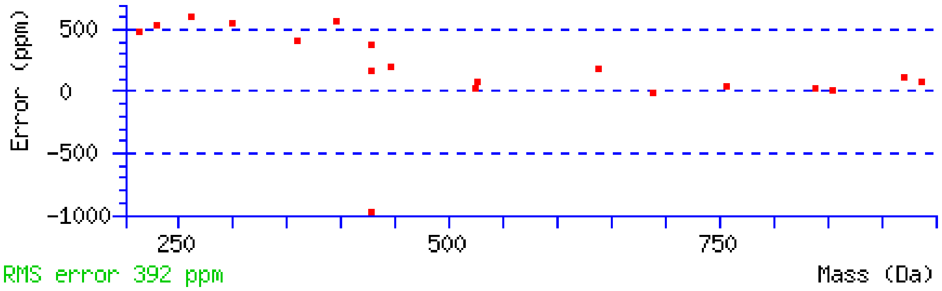

NCBI **BLAST** search of NNSELSVLK
(Parameters: blastp, nr protein database, expect=20000, no filter, PAM30)
Other BLAST web gateways

All matches to this query

Score	Mr(calc):	Delta	Sequence
31.4	1082.5009	0.0023	NNSELSVLK
17.9	1082.5038	-0.0007	EVTKDQSGK
16.5	1082.5009	0.0023	NNSELSVLK
13.0	1082.5009	0.0023	TKPSKEEGK

10.1	1082.5038	-0.0007	EVTKDQSGK
8.7	1082.5009	0.0022	LSLEDGGKGK
8.4	1082.5037	-0.0005	DLREVFSK
8.2	1082.5037	-0.0005	XSSFDLRK
7.6	1082.5009	0.0022	YLRTARK
7.4	1082.5009	0.0023	TKPSKEEGK

Mascot: http://www.matrixscience.com/

Mascot Search Results

Peptide View

MS/MS Fragmentation of **AALEDSSGSSELQEIMR**
Found in **IPI00290337**, Tax_Id=9606 Gene_Symbol=EPS8 Epidermal growth factor receptor kinase substrate 8

Match to Query 35526: 1911.819776 from(956.917164,2+)
Title: RawFile: CZ_270810_FT2.raw FinneganScanNumber: 2559 _iso_
Data file C:\Users\cunningd\Documents\Mass Spec\Triple SILAC Experiments\270810 NO NaP Eps8 FGF2 vs Eps8 FGF2SU5402 vs Eps8 FGF2 Dasatinib\Expt 1 and expt 2\combined\allSpectra.CID.iso_0.msm

Click mouse within plot area to zoom in by factor of two about that point
Or, Plot from 300 to 1900 Da Full range

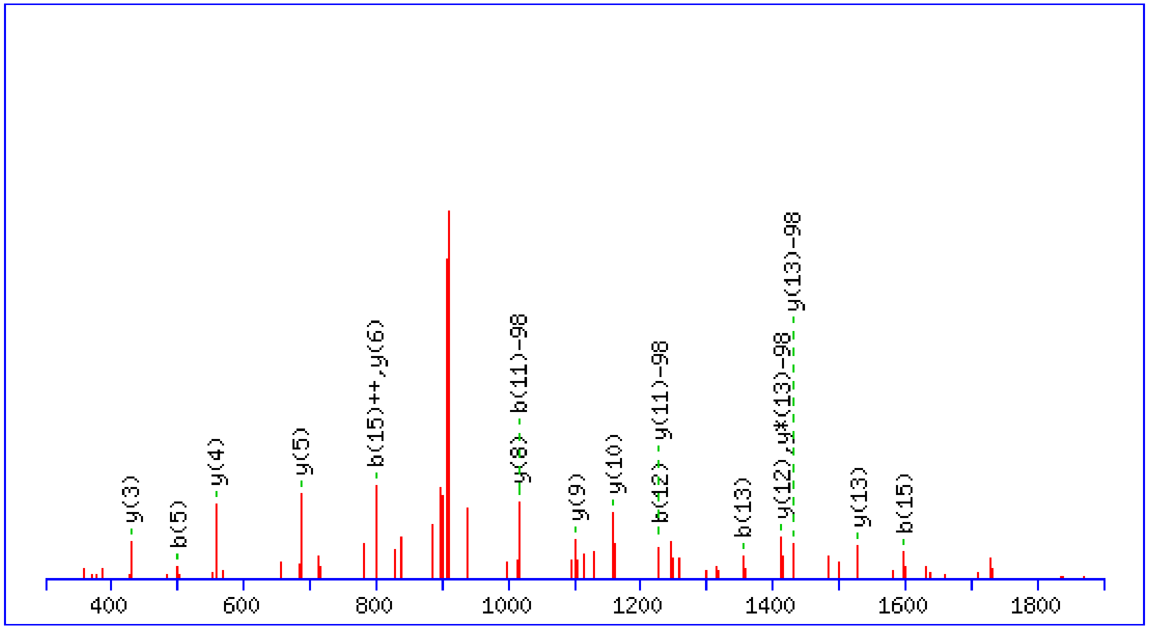

Monoisotopic mass of neutral peptide Mr(calc): 1911.8160
Fixed modifications: Carbamidomethyl (C)
Variable modifications:
S7 : Phospho (ST), with neutral losses 0.0000(shown in table), 97.9769
R17 : Label:13C(6)15N(4) (R)
Ions Score: 68 **Expect:** 0.00014

Matches (Bold Red): 18/240 fragment ions using 26 most intense peaks

#	b	b^{++}	b^{*}	b^{***}	b^{0}	b^{0++}	Seq.	y	y^{++}	y^{*}	y^{***}	y^{0}	y^{0++}	#
1	72.0444	36.5258					A							17
2	143.0815	72.0444					A	1841.7862	921.3967	1824.7597	912.8835	1823.7756	912.3915	16
3	256.1656	128.5864					L	1770.7491	885.8782	1753.7225	877.3649	1752.7385	876.8729	15
4	385.2082	193.1077			367.1976	184.1024	E	1657.6650	829.3362	1640.6385	820.8229	1639.6545	820.3309	14
5	**500.2351**	250.6212			482.2245	241.6159	D	**1528.6224**	764.8149	1511.5959	756.3016	1510.6119	755.8096	13
6	587.2671	294.1372			569.2566	285.1319	S	**1413.5955**	707.3014	1396.5689	698.7881	1395.5849	698.2961	12
7	754.2655	377.6364			736.2549	368.6311	S	1326.5635	663.7854	1309.5369	655.2721	1308.5529	654.7801	11
8	811.2869	406.1471			793.2764	397.1418	G	**1159.5651**	580.2862	1142.5386	571.7729	1141.5546	571.2809	10
9	898.3190	449.6631			880.3084	440.6578	S	**1102.5437**	551.7755	1085.5171	543.2622	1084.5331	542.7702	9
10	985.3510	493.1791			967.3404	484.1739	S	**1015.5116**	508.2595	998.4851	499.7462	997.5011	499.2542	8
11	1114.3936	557.7004			1096.3830	548.6952	E	928.4796	464.7434	911.4530	456.2302	910.4690	455.7382	7
12	**1227.4777**	614.2425			1209.4671	605.2372	L	**799.4370**	400.2221	782.4105	391.7089	781.4264	391.2169	6
13	**1355.5362**	678.2718	1338.5097	669.7585	1337.5257	669.2665	Q	**686.3529**	343.6801	669.3264	335.1668	668.3424	334.6748	5
14	1484.5788	742.7931	1467.5523	734.2798	1466.5683	733.7878	E	**558.2944**	279.6508	541.2678	271.1375	540.2838	270.6455	4
15	**1597.6629**	**799.3351**	1580.6363	790.8218	1579.6523	790.3298	I	**429.2518**	215.1295	412.2252	206.6162			3
16	1728.7034	864.8553	1711.6768	856.3421	1710.6928	855.8500	M	316.1677	158.5875	299.1412	150.0742			2
17							R	185.1272	93.0672	168.1007	84.5540			1

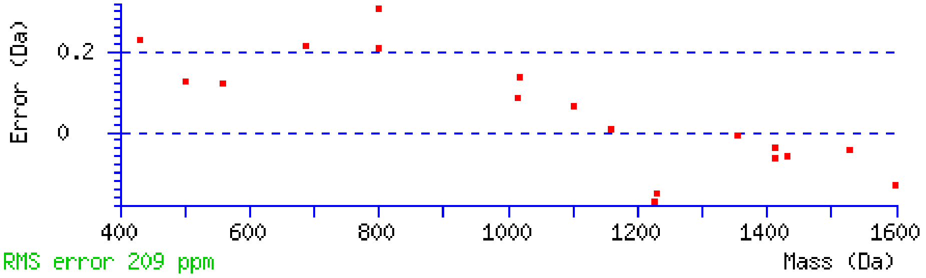

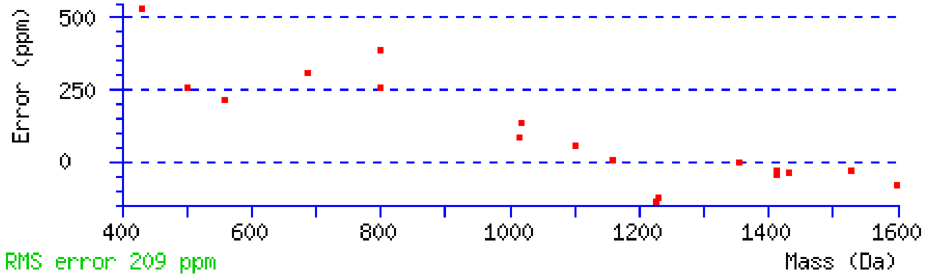

NCBI **BLAST** search of AALEDSSGSSELQEIMR
(Parameters: blastp, nr protein database, expect=20000, no filter, PAM30)
Other BLAST web gateways

All matches to this query

Score	Mr(calc):	Delta	Sequence
68.3	1911.8160	0.0038	AALEDSSGSSELQEIMR
68.3	1911.8160	0.0038	AALEDSSGSSELQEIMR
40.0	1911.8160	0.0038	AALEDSSGSSELQEIMR
27.7	1911.8160	0.0038	AALEDSSGSSELQEIMR
14.5	1911.8294	-0.0096	RASPENENESNQSGNNR
13.2	1911.8239	-0.0041	REVTSSPLYDEQGDAR
13.2	1911.8239	-0.0041	REVTSSPLYDEQGDAR
12.7	1911.8239	-0.0041	REVTSSPLYDEQGDAR
9.8	1911.8248	-0.0050	AGSQVMMQLSNEPFQR
9.3	1911.8303	-0.0105	PSMPAPGTRQENGMATR

Mascot: http://www.matrixscience.com/

Mascot Search Results

Peptide View

MS/MS Fragmentation of **VYSQITVQK**
Found in **IPI00794000**, Tax_Id=9606 Gene_Symbol=EPS8 cDNA, FLJ79138, highly similar to Epidermal growth factor receptor kinase substrate 8

Match to Query 16917: 1144.555848 from(573.285200,2+)
Title: RawFile: CZ_270810_eps82b.raw FinneganScanNumber: 1258 _nix_
Data file C:\Users\cunningd\Documents\Mass Spec\Triple SILAC Experiments\270810 NO NaP Eps8 FGF2 vs Eps8 FGF2SU5402 vs Eps8 FGF2 Dasatinib\Expt 1 and expt 2\combined\allSpectra.CID.iso_0.msm

Click mouse within plot area to zoom in by factor of two about that point
Or, Plot from 200 to 1050 Da Full range

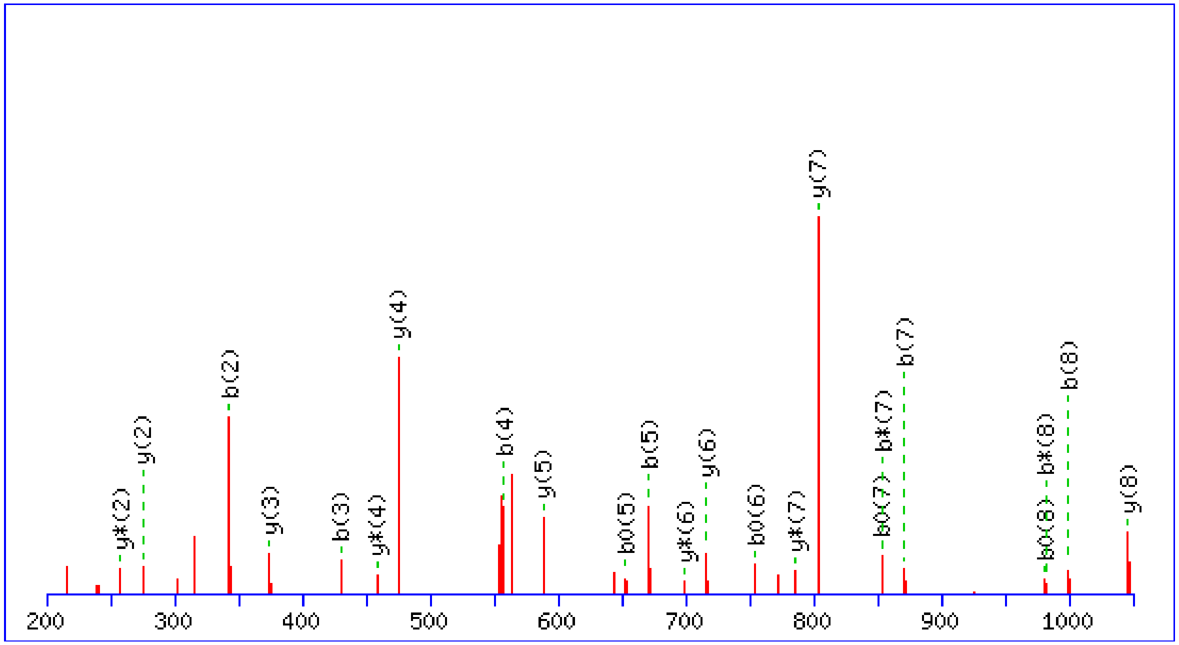

Monoisotopic mass of neutral peptide Mr(calc): 1144.5529
Fixed modifications: Carbamidomethyl (C)
Variable modifications:
Y2 : Phospho (Y)

Ions Score: 47 **Expect:** 0.03
Matches (Bold Red): 23/80 fragment ions using 41 most intense peaks

#	b	b++	b*	b*++	b^{0}	b^{0++}	Seq.	y	y++	y*	y*++	y^{0}	y^{0++}	#
1	100.0757	50.5415					V							9
2	**343.1053**	172.0563					Y	**1046.4918**	523.7495	1029.4652	515.2363	1028.4812	514.7443	8
3	**430.1374**	215.5723			412.1268	206.5670	S	**803.4621**	402.2347	**786.4356**	393.7214	785.4516	393.2294	7
4	**558.1959**	279.6016	541.1694	271.0883	540.1854	270.5963	Q	**716.4301**	358.7187	**699.4036**	350.2054	698.4196	349.7134	6
5	**671.2800**	336.1436	654.2535	327.6304	**653.2694**	327.1384	I	**588.3715**	294.6894	571.3450	286.1761	570.3610	285.6841	5
6	772.3277	386.6675	755.3011	378.1542	**754.3171**	377.6622	T	**475.2875**	238.1474	**458.2609**	229.6341	457.2769	229.1421	4
7	**871.3961**	436.2017	**854.3696**	427.6884	**853.3855**	427.1964	V	**374.2398**	187.6235	357.2132	179.1103			3
8	**999.4547**	500.2310	**982.4281**	491.7177	**981.4441**	491.2257	Q	**275.1714**	138.0893	**258.1448**	129.5761			2
9							K	147.1128	74.0600	130.0863	65.5468			1

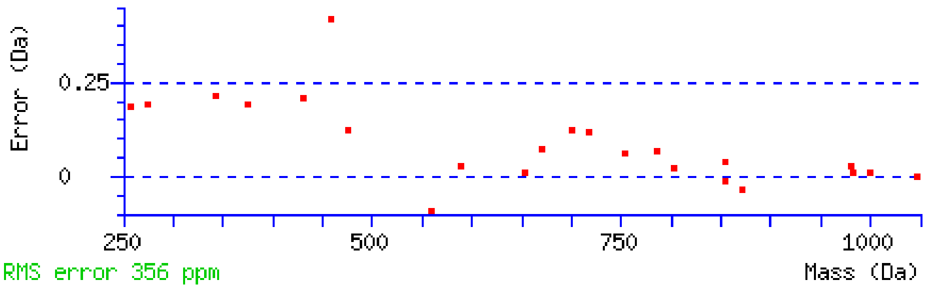

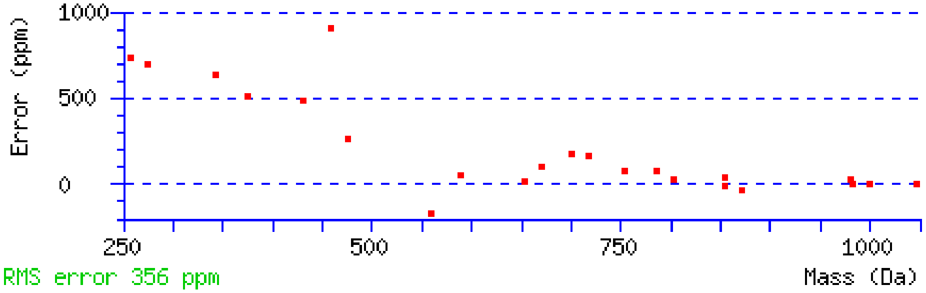

NCBI **BLAST** search of VYSQITVQK
(Parameters: blastp, nr protein database, expect=20000, no filter, PAM30)
Other BLAST web gateways

All matches to this query

Score	Mr(calc):	Delta	Sequence
47.3	1144.5529	0.0029	VYSQITVQK
32.5	1144.5529	0.0029	VYSQITVQK
16.8	1144.5558	0.0000	VYKSSPSTGK
16.6	1144.5584	-0.0025	RQTPLTKK

13.8	1144.5596	-0.0038	KPPTSPVKK
11.8	1144.5547	0.0012	VMIDGHDSKK
11.4	1144.5585	-0.0026	KEGEGGIITR
11.2	1144.5529	0.0029	VYSQITVQK
10.1	1144.5529	0.0029	PFSSGKTITK
9.7	1144.5613	-0.0055	KSGAMPKNTK

Mascot: http://www.matrixscience.com/

Mascot Search Results

Peptide View

MS/MS Fragmentation of **QYHEQEETPEMMAAR**
Found in **IPI00290337**, Tax_Id=9606 Gene_Symbol=EPS8 Epidermal growth factor receptor kinase substrate 8

Match to Query 5798: 1960.743486 from(654.588438,3+)
Title: RawFile: CZ_140510_DC_EPS8_tio2_100517084708.raw FinneganScanNumber: 720 _sil_
Data file C:\Users\cunningd\Documents\Mass Spec\Triple SILAC Experiments\070510 Eps8 FGF2 vs Eps8 FGF2 SU5402 vs Eps8 FGF2 Dasatinib + 170510 160610rpts enrich and FT\combined\allSpectra.CID.sil0_0.msm

Click mouse within plot area to zoom in by factor of two about that point
Or, Plot from 200 to 1600 Da Full range

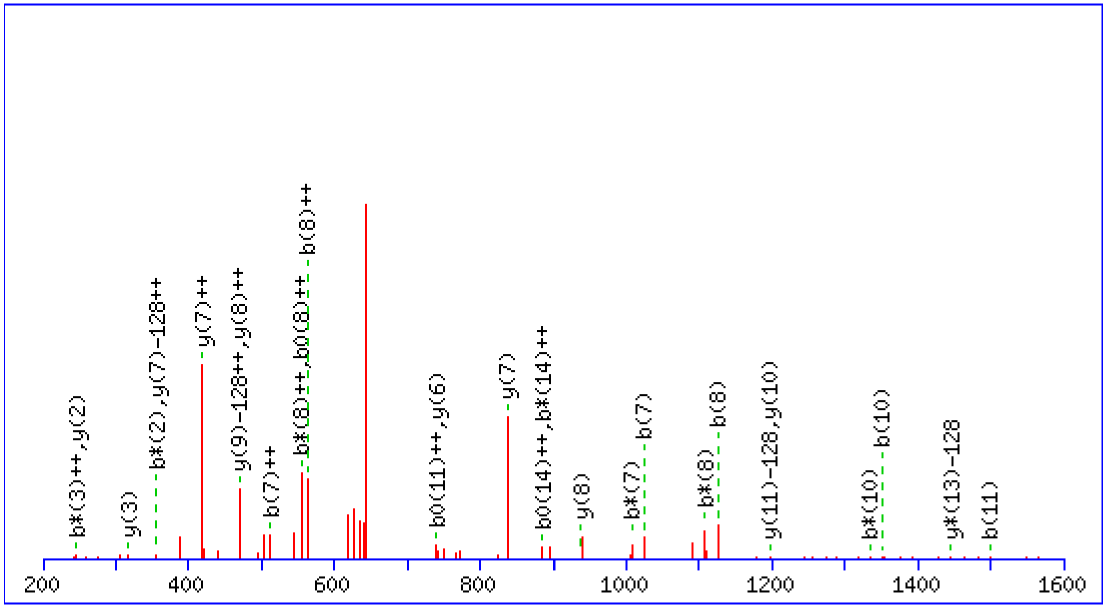

Monoisotopic mass of neutral peptide Mr(calc): 1960.7332
Fixed modifications: Carbamidomethyl (C)
Variable modifications:
Y2 : Phospho (Y)
M11 : Oxidation (M), with neutral losses 0.0000(shown in table), 63.9983
M12 : Oxidation (M), with neutral losses 0.0000(shown in table), 63.9983
Ions Score: 31 **Expect:** 0.054
Matches (Bold Red): 28/238 fragment ions using 33 most intense peaks

#	b	b++	b*	b*++	b0	b0++	Seq.	y	y++	y*	y*++	y0	y0++	#
1	129.0659	65.0366	112.0393	56.5233			Q							15
2	372.0955	186.5514	**355.0690**	178.0381			Y	1833.6819	917.3446	1816.6554	908.8313	1815.6714	908.3393	14
3	509.1544	255.0808	492.1279	**246.5676**			H	1590.6523	795.8298	1573.6257	787.3165	1572.6417	786.8245	13
4	638.1970	319.6021	621.1705	311.0889	620.1864	310.5969	E	1453.5934	727.3003	1436.5668	718.7871	1435.5828	718.2950	12
5	766.2556	383.6314	749.2290	375.1182	748.2450	374.6262	Q	1324.5508	662.7790	1307.5242	654.2658	1306.5402	653.7737	11
6	895.2982	448.1527	878.2716	439.6395	877.2876	439.1474	E	**1196.4922**	598.7497	1179.4657	590.2365	1178.4816	589.7445	10
7	**1024.3408**	**512.6740**	**1007.3142**	504.1608	1006.3302	503.6687	E	1067.4496	534.2284	1050.4231	525.7152	1049.4391	525.2232	9
8	**1125.3885**	**563.1979**	**1108.3619**	**554.6846**	1107.3779	**554.1926**	T	**938.4070**	**469.7072**	921.3805	461.1939	920.3965	460.7019	8
9	1222.4412	611.7242	1205.4147	603.2110	1204.4307	602.7190	P	**837.3593**	**419.1833**	820.3328	410.6700	819.3488	410.1780	7
10	**1351.4838**	676.2455	**1334.4573**	667.7323	1333.4732	667.2403	E	**740.3066**	370.6569	723.2800	362.1437	722.2960	361.6516	6
11	**1498.5192**	749.7632	1481.4927	741.2500	1480.5087	**740.7580**	M	611.2640	306.1356	594.2374	297.6224			5
12	1645.5546	823.2809	1628.5281	814.7677	1627.5441	814.2757	M	464.2286	232.6179	447.2020	224.1047			4
13	1716.5917	858.7995	1699.5652	850.2862	1698.5812	849.7942	A	**317.1932**	159.1002	300.1666	150.5870			3
14	1787.6288	894.3181	1770.6023	**885.8048**	1769.6183	**885.3128**	A	**246.1561**	123.5817	229.1295	115.0684			2
15							R	175.1190	88.0631	158.0924	79.5498			1

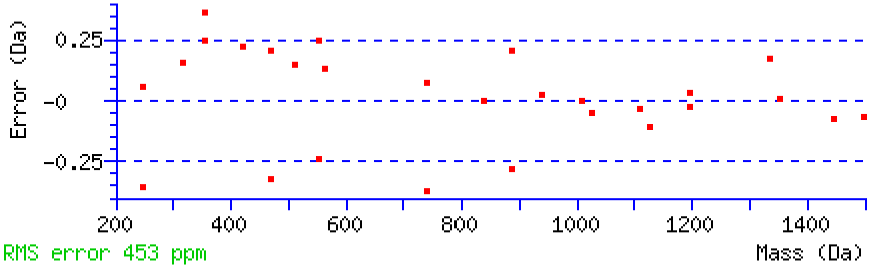

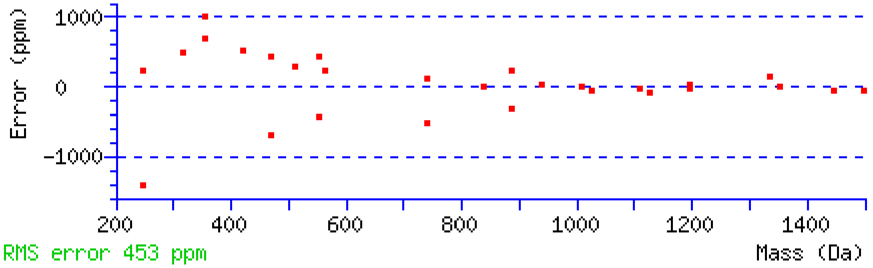

NCBI **BLAST** search of QYHEQEETPEMMAAR
(Parameters: blastp, nr protein database, expect=20000, no filter, PAM30)
Other BLAST web gateways

All matches to this query

Score	Mr(calc):	Delta	Sequence
30.9	1960.7332	0.0103	QYHEQEETPEMMAAR
9.8	1960.7332	0.0103	QYHEQEETPEMMAAR

6.0	1960.7423	0.0012	QPSMSETMPLYTLCK
4.4	1960.7569	-0.0134	KCLEMGMKMESVQSK
4.0	1960.7452	-0.0017	YSSTSSSASSTAAPASPSR
4.0	1960.7452	-0.0017	YSSTSSSASSTAAPASPSR
4.0	1960.7452	-0.0017	YSSTSSSASSTAAPASPSR
3.5	1960.7348	0.0087	ADAECYTAMKIAEANK
1.8	1960.7569	-0.0134	SQVSEMKMGMELCKK
1.5	1960.7478	-0.0043	IQSDSIIGTSLGMPMR

Mascot: http://www.matrixscience.com/

Mascot Search Results

Peptide View

MS/MS Fragmentation of **ADPPYTHTIQK**
Found in **IPI00290337**, Tax_Id=9606 Gene_Symbol=EPS8 Epidermal growth factor receptor kinase substrate 8

Match to Query 2797: 1349.605512 from(675.810032,2+)
Title: RawFile: AJC_DC_EPS8_TiO2_cap_150610.raw FinneganScanNumber: 441 _sil_
Data file C:\Users\cunningd\Documents\Mass Spec\Triple SILAC Experiments\070510 Eps8 FGF2 vs Eps8 FGF2 SU5402 vs Eps8 FGF2 Dasatinib + 170510 160610rpts enrich and FT\combined\allSpectra.CID.sil0_0.msm

Click mouse within plot area to zoom in by factor of two about that point
Or, [Plot from] 250 to 1250 Da [Full range]

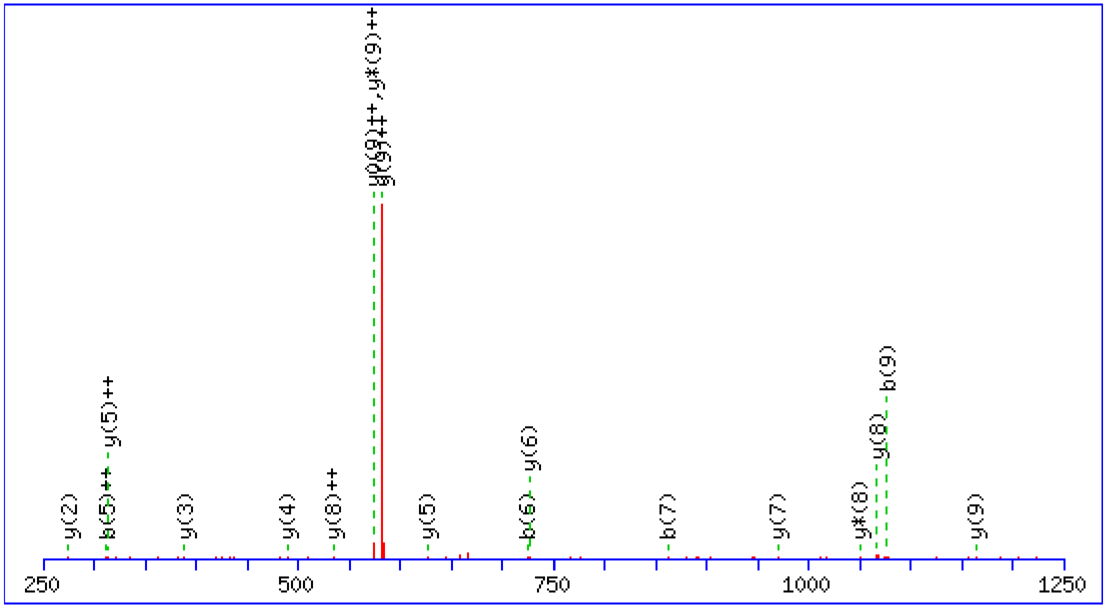

Monoisotopic mass of neutral peptide Mr(calc): 1349.6017
Fixed modifications: Carbamidomethyl (C)
Variable modifications:
Y5 : Phospho (Y)
Ions Score: 39 **Expect:** 0.01
Matches (Bold Red): 18/94 fragment ions using 46 most intense peaks

#	b	b^{++}	b^{*}	b^{*++}	b^{0}	b^{0++}	Seq.	y	y^{++}	y^{*}	y^{*++}	y^{0}	y^{0++}	#
1	72.0444	36.5258					A							11
2	187.0713	94.0393			169.0608	85.0340	D	1279.5718	640.2896	1262.5453	631.7763	1261.5613	631.2843	10
3	284.1241	142.5657			266.1135	133.5604	P	**1164.5449**	**582.7761**	1147.5183	**574.2628**	1146.5343	**573.7708**	9
4	381.1769	191.0921			363.1663	182.0868	P	**1067.4921**	**534.2497**	**1050.4656**	525.7364	1049.4816	525.2444	8
5	624.2065	**312.6069**			606.1959	303.6016	Y	**970.4394**	485.7233	953.4128	477.2100	952.4288	476.7180	7
6	**725.2542**	363.1307			707.2436	354.1255	T	**727.4097**	364.2085	710.3832	355.6952	709.3992	355.2032	6
7	**862.3131**	431.6602			844.3025	422.6549	H	**626.3620**	**313.6847**	609.3355	305.1714	608.3515	304.6794	5
8	963.3608	482.1840			945.3502	473.1787	T	**489.3031**	245.1552	472.2766	236.6419	471.2926	236.1499	4
9	**1076.4448**	538.7261			1058.4343	529.7208	I	**388.2554**	194.6314	371.2289	186.1181			3
10	1204.5034	602.7554	1187.4769	594.2421	1186.4929	593.7501	Q	**275.1714**	138.0893	258.1448	129.5761			2
11							K	147.1128	74.0600	130.0863	65.5468			1

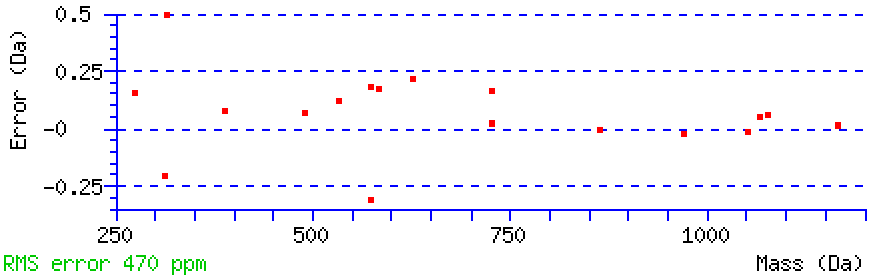

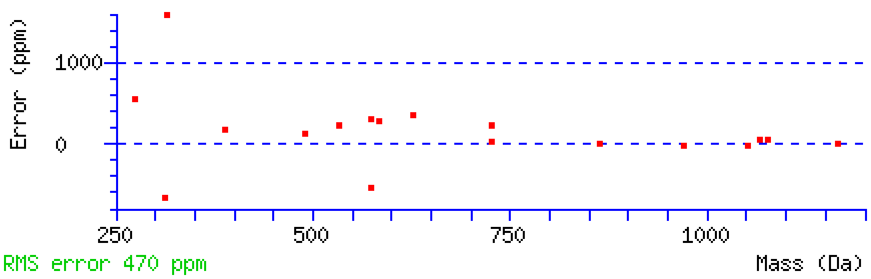

NCBI **BLAST** search of ADPPYTHTIQK
(Parameters: blastp, nr protein database, expect=20000, no filter, PAM30)
Other BLAST web gateways

All matches to this query

Score	Mr(calc):	Delta	Sequence
39.4	1349.6017	0.0038	ADPPYTHTIQK
30.2	1349.6017	0.0038	ADPPYTHTIQK
23.5	1349.6017	0.0038	ADPPYTHTIQK
12.6	1349.6129	-0.0074	SFAGNLNTYKR
6.8	1349.6034	0.0021	WPMDRTEGGSAK
5.1	1349.6145	-0.0090	WVKTNSSKLK

5.0	1349.6089	-0.0033	ADSEQPSKRPR
5.0	1349.6034	0.0021	WEIISCENSGR
4.9	1349.6034	0.0021	QGWEQTCNLSK
4.9	1349.6089	-0.0034	TRDLPGSQSPGR

Mascot: http://www.matrixscience.com/

Mascot Search Results

Peptide View

MS/MS Fragmentation of **AAEAFSELSKR**
Found in **IPI00290337**, Tax_Id=9606 Gene_Symbol=EPS8 Epidermal growth factor receptor kinase substrate 8

Match to Query 2468: 1287.592778 from(644.803665,2+)
Title: RawFile: CZ_140510_DC_EPS8_tio2_100517084708.raw FinneganScanNumber: 1437 _sil_
Data file C:\Users\cunningd\Documents\Mass Spec\Triple SILAC Experiments\070510 Eps8 FGF2 vs Eps8 FGF2 SU5402 vs Eps8 FGF2 Dasatinib + 170510 160610rpts enrich and FT\combined\allSpectra.CID.sil0_0.msm

Click mouse within plot area to zoom in by factor of two about that point
Or, Plot from 200 to 1250 Da Full range

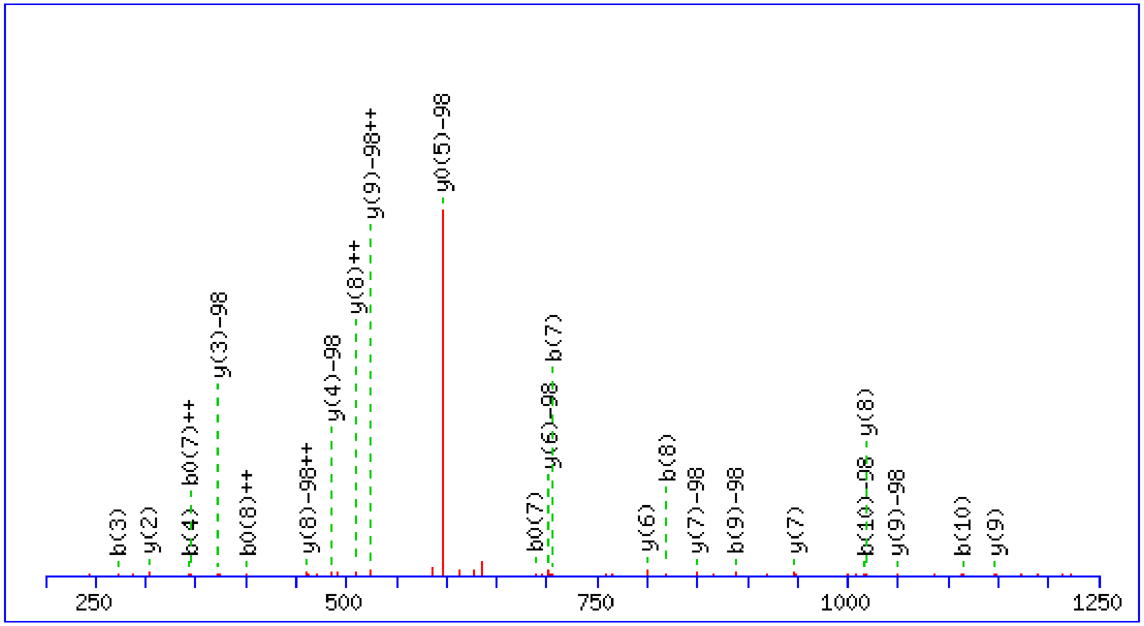

Monoisotopic mass of neutral peptide Mr(calc): 1287.5860
Fixed modifications: Carbamidomethyl (C)
Variable modifications:
S9 : Phospho (ST), with neutral losses 97.9769(shown in table), 0.0000
Ions Score: 46 **Expect:** 0.0023
Matches (Bold Red): 24/152 fragment ions using 40 most intense peaks

#	b	b++	b*	b*++	b0	b0++	Seq.	y	y++	y*	y*++	y0	y0++	#
1	72.0444	36.5258					A							11
2	143.0815	72.0444					A	1119.5793	560.2933	1102.5527	551.7800	1101.5687	551.2880	10
3	**272.1241**	136.5657			254.1135	127.5604	E	**1048.5422**	**524.7747**	1031.5156	516.2615	1030.5316	515.7694	9
4	**343.1612**	172.0842			325.1506	163.0790	A	919.4996	**460.2534**	902.4730	451.7402	901.4890	451.2481	8
5	490.2296	245.6185			472.2191	236.6132	F	**848.4625**	424.7349	831.4359	416.2216	830.4519	415.7296	7
6	577.2617	289.1345			559.2511	280.1292	S	**701.3941**	351.2007	684.3675	342.6874	683.3835	342.1954	6
7	**706.3042**	353.6558			**688.2937**	**344.6505**	E	614.3620	307.6847	597.3355	299.1714	**596.3515**	298.6794	5
8	**819.3883**	410.1978			801.3777	**401.1925**	L	**485.3194**	243.1634	468.2929	234.6501	467.3089	234.1581	4
9	**888.4098**	444.7085			870.3992	435.7032	S	**372.2354**	186.6213	355.2088	178.1080	354.2248	177.6160	3
10	**1016.5047**	508.7560	999.4782	500.2427	998.4942	499.7507	K	**303.2139**	152.1106	286.1874	143.5973			2
11							R	175.1190	88.0631	158.0924	79.5498			1

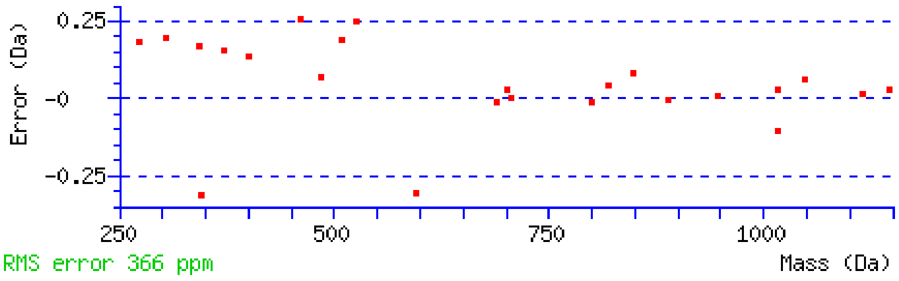

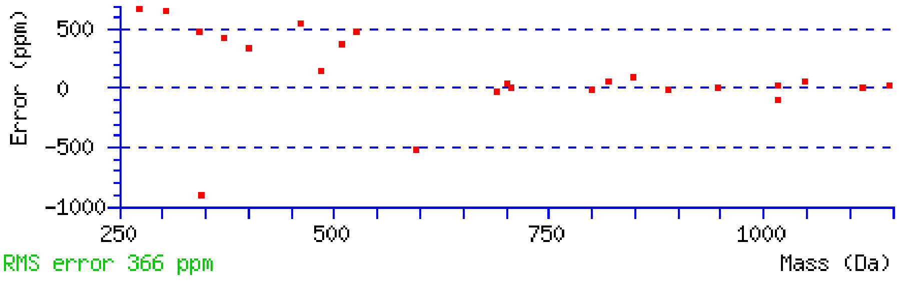

NCBI **BLAST** search of AAEAFSELSKR
(Parameters: blastp, nr protein database, expect=20000, no filter, PAM30)
Other BLAST web gateways

All matches to this query

Score	Mr(calc):	Delta	Sequence
46.5	1287.5860	0.0068	AAEAFSELSKR
32.0	1287.5860	0.0068	AAEAFSELSKR
18.4	1287.5860	0.0068	DQKLFVSESR
15.4	1287.5907	0.0020	RFAGAGCVSKR
15.1	1287.5860	0.0068	DQKLFVSESR

13.9	1287.5972	-0.0045	VNELEPHSRK
12.9	1287.5972	-0.0044	NSAAAKYLTNR
9.3	1287.5894	0.0034	LTGMLSESKSR
9.1	1287.5924	0.0003	MGGCRAGREPR
9.0	1287.5874	0.0054	PWGQSAGPRPR

Mascot: http://www.matrixscience.com/

Mascot Search Results

Peptide View

MS/MS Fragmentation of **MISNADPSIPPPPR**
Found in **IPI00290337**, Tax_Id=9606 Gene_Symbol=EPS8 Epidermal growth factor receptor kinase substrate 8

Match to Query 2678: 1592.743186 from(797.378869,2+)
Title: RawFile: CZ_140510_DC_EPS8_tio2_100517084708.raw FinneganScanNumber: 1671 _sil_
Data file C:\Users\cunningd\Documents\Mass Spec\Triple SILAC Experiments\070510 Eps8 FGF2 vs Eps8 FGF2 SU5402 vs Eps8 FGF2 Dasatinib + 170510 160610rpts enrich and FT\combined\allSpectra.CID.sil1_0.msm

Click mouse within plot area to zoom in by factor of two about that point
Or, Plot from 200 to 1600 Da Full range

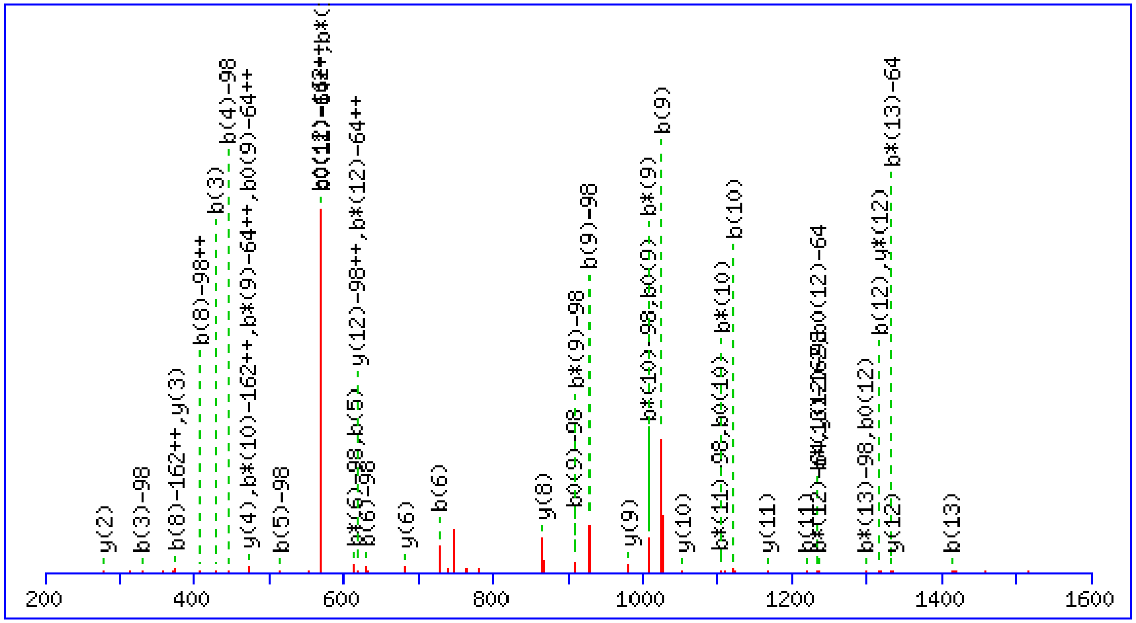

Monoisotopic mass of neutral peptide Mr(calc): 1592.7365
Fixed modifications: Carbamidomethyl (C),Label:13C(6) (R),Label:2H(4) (K)
Variable modifications:
M1 : Oxidation (M), with neutral losses 0.0000(shown in table), 63.9983
S3 : Phospho (ST), with neutral losses 97.9769(shown in table), 0.0000
Ions Score: 49 **Expect:** 0.0012

Matches (Bold Red): 51/342 fragment ions using 69 most intense peaks

#	b	b^{++}	b^{*}	b^{*++}	b^{0}	b^{0++}	Seq.	y	y^{++}	y^{*}	y^{*++}	y^{0}	y^{0++}	#
1	148.0427	74.5250					M							14
2	261.1267	131.0670					I	1348.7315	674.8694	1331.7049	666.3561	1330.7209	665.8641	13
3	**330.1482**	165.5777			312.1376	156.5725	S	**1235.6474**	**618.3274**	1218.6209	609.8141	1217.6369	609.3221	12
4	**444.1911**	222.5992	427.1646	214.0859	426.1806	213.5939	N	**1166.6260**	583.8166	1149.5994	575.3034	1148.6154	574.8113	11
5	**515.2282**	258.1178	498.2017	249.6045	497.2177	249.1125	A	**1052.5830**	526.7952	1035.5565	518.2819	1034.5725	517.7899	10
6	**630.2552**	315.6312	**613.2286**	307.1180	612.2446	306.6259	D	**981.5459**	491.2766	964.5194	482.7633	963.5354	482.2713	9
7	727.3079	364.1576	710.2814	355.6443	709.2974	355.1523	P	**866.5190**	433.7631	849.4924	425.2499	848.5084	424.7579	8
8	814.3400	**407.6736**	797.3134	399.1604	796.3294	398.6683	S	769.4662	385.2368	752.4397	376.7235	751.4557	376.2315	7
9	**927.4240**	464.2157	**910.3975**	455.7024	**909.4135**	455.2104	I	**682.4342**	341.7207	665.4077	333.2075			6
10	1024.4768	512.7420	**1007.4503**	504.2288	1006.4662	503.7368	P	**569.3501**	285.1787	552.3236	276.6654			5
11	1121.5296	561.2684	**1104.5030**	552.7551	1103.5190	552.2631	P	**472.2974**	236.6523	455.2708	228.1390			4
12	1218.5823	609.7948	1201.5558	601.2815	1200.5718	600.7895	P	**375.2446**	188.1259	358.2181	179.6127			3
13	1315.6351	658.3212	**1298.6085**	649.8079	1297.6245	649.3159	P	**278.1918**	139.5996	261.1653	131.0863			2
14							R	181.1391	91.0732	164.1125	82.5599			1

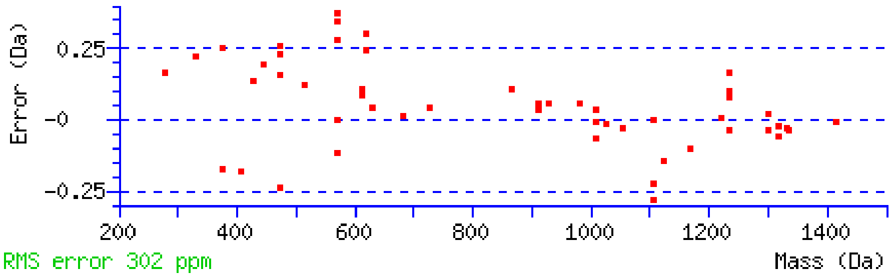

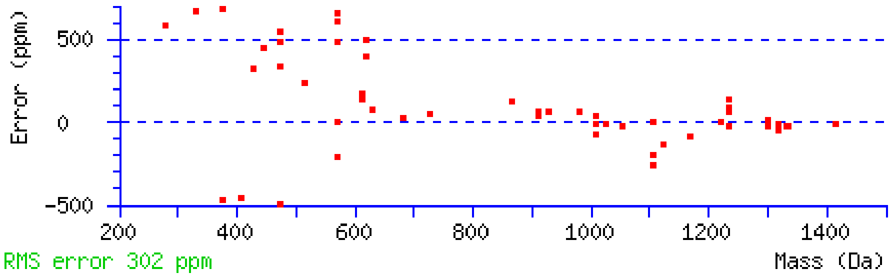

NCBI **BLAST** search of MISNADPSIPPPPR
(Parameters: blastp, nr protein database, expect=20000, no filter, PAM30)
Other BLAST web gateways

All matches to this query

Score	Mr(calc):	Delta	Sequence
49.1	1592.7365	0.0067	MISNADPSIPPPPR

25.4	1592.7365	0.0067	MISNADPSIPPPPR
3.7	1592.7499	-0.0067	QNGGLPRSEQPYR
2.7	1592.7330	0.0102	RPHQAQADDDESGR
2.4	1592.7403	0.0029	ELSLYDGSRLLR
2.2	1592.7374	0.0058	ELDQQGQQVEEER
1.2	1592.7365	0.0067	DLQDNFLTALAMR
1.0	1592.7510	-0.0078	VLWSTQKDCPGSK
0.7	1592.7496	-0.0064	RSTSTGNKESSSTR
0.7	1592.7496	-0.0064	RSTSTGNKESSSTR

Mascot: http://www.matrixscience.com/

Mascot Search Results

Peptide View

MS/MS Fragmentation of **AALEDSSGSSELQEIMRR**
Found in **IPI00290337**, Tax_Id=9606 Gene_Symbol=EPS8 Epidermal growth factor receptor kinase substrate 8

Match to Query 6362: 2153.878782 from(718.966870,3+)
Title: RawFile: CZ_070510_DC_EPS8_tio2.raw FinneganScanNumber: 1888 _sil_
Data file C:\Users\cunningd\Documents\Mass Spec\Triple SILAC Experiments\070510 Eps8 FGF2 vs Eps8 FGF2 SU5402 vs Eps8 FGF2 Dasatinib + 170510 160610rpts enrich and FT\combined\allSpectra.CID.sil0_0.msm

Click mouse within plot area to zoom in by factor of two about that point
Or, Plot from 200 to 1600 Da Full range

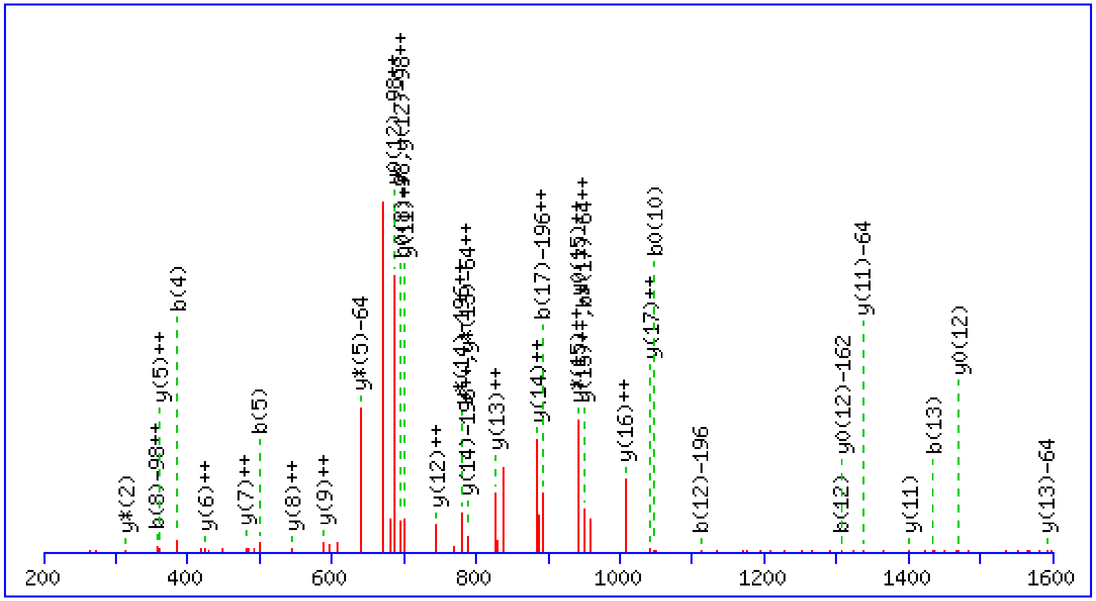

Monoisotopic mass of neutral peptide Mr(calc): 2153.8701
Fixed modifications: Carbamidomethyl (C)
Variable modifications:
S6 : Phospho (ST), with neutral losses 0.0000(shown in table), 97.9769
S9 : Phospho (ST), with neutral losses 0.0000(shown in table), 97.9769
M16 : Oxidation (M), with neutral losses 0.0000(shown in table), 63.9983
Ions Score: 30 **Expect:** 0.11
Matches (Bold Red): 36/430 fragment ions using 76 most intense peaks

#	b	b^{++}	b^{*}	b^{*++}	b^{0}	b^{0++}	Seq.	y	y^{++}	y^{*}	y^{*++}	y^{0}	y^{0++}	#
1	72.0444	36.5258					A							18
2	143.0815	72.0444					A	2083.8403	**1042.4238**	2066.8137	1033.9105	2065.8297	1033.4185	17
3	256.1656	128.5864					L	2012.8032	**1006.9052**	1995.7766	998.3920	1994.7926	997.8999	16
4	**385.2082**	193.1077			367.1976	184.1024	E	1899.7191	**950.3632**	1882.6926	**941.8499**	1881.7086	**941.3579**	15
5	**500.2351**	250.6212			482.2245	241.6159	D	1770.6765	**885.8419**	1753.6500	877.3286	1752.6660	876.8366	14
6	667.2335	334.1204			649.2229	325.1151	S	1655.6496	**828.3284**	1638.6230	819.8152	1637.6390	819.3231	13
7	754.2655	377.6364			736.2549	368.6311	S	1488.6512	**744.8293**	1471.6247	736.3160	**1470.6407**	735.8240	12
8	811.2869	406.1471			793.2764	397.1418	G	**1401.6192**	**701.3132**	1384.5927	692.8000	1383.6086	692.3080	11
9	978.2853	489.6463			960.2747	480.6410	S	1344.5977	672.8025	1327.5712	664.2892	1326.5872	663.7972	10
10	1065.3173	533.1623			**1047.3068**	524.1570	S	1177.5994	**589.3033**	1160.5728	580.7901	1159.5888	580.2980	9
11	1194.3599	597.6836			1176.3494	588.6783	E	1090.5674	**545.7873**	1073.5408	537.2740	1072.5568	536.7820	8
12	**1307.4440**	654.2256			1289.4334	645.2203	L	961.5248	**481.2660**	944.4982	472.7527	943.5142	472.2607	7
13	**1435.5026**	718.2549	1418.4760	709.7416	1417.4920	709.2496	Q	848.4407	**424.7240**	831.4142	416.2107	830.4301	415.7187	6
14	1564.5452	782.7762	1547.5186	774.2629	1546.5346	773.7709	E	720.3821	**360.6947**	703.3556	352.1814	702.3716	351.6894	5
15	1677.6292	839.3182	1660.6027	830.8050	1659.6187	830.3130	I	591.3395	296.1734	574.3130	287.6601			4
16	1824.6646	912.8359	1807.6381	904.3227	1806.6541	903.8307	M	478.2555	239.6314	461.2289	231.1181			3
17	1980.7657	990.8865	1963.7392	982.3732	1962.7552	981.8812	R	331.2201	166.1137	**314.1935**	157.6004			2
18							R	175.1190	88.0631	158.0924	79.5498			1

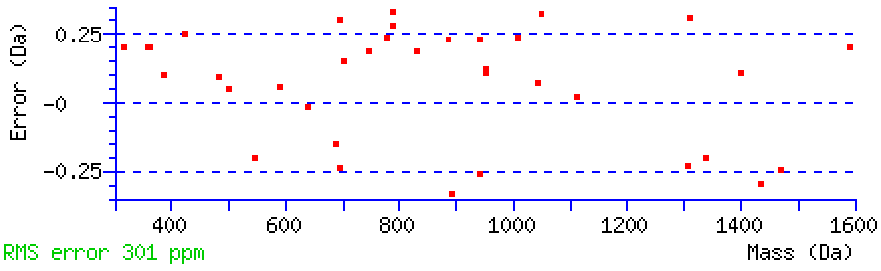

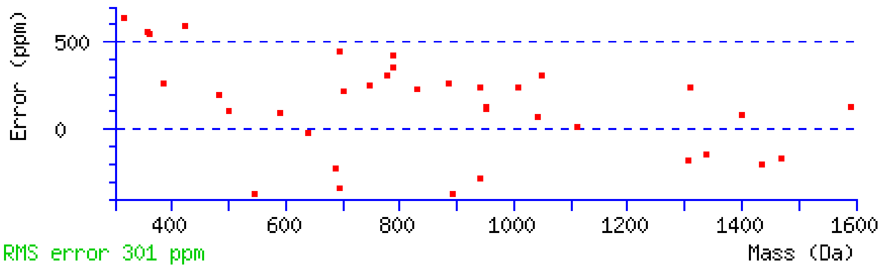

NCBI **BLAST** search of AALEDSSGSSELQEIMRR
(Parameters: blastp, nr protein database, expect=20000, no filter, PAM30)
Other BLAST web gateways

All matches to this query

Score	Mr(calc):	Delta	Sequence

29.7	2153.8701	0.0087	AALEDSSGSSELQEIMRR
27.0	2153.8701	0.0087	AALEDSSGSSELQEIMRR
23.5	2153.8701	0.0087	AALEDSSGSSELQEIMRR
22.4	2153.8701	0.0087	AALEDSSGSSELQEIMRR
19.8	2153.8701	0.0087	AALEDSSGSSELQEIMRR
14.5	2153.8701	0.0087	AALEDSSGSSELQEIMRR
2.7	2153.8911	-0.0123	AAMMEPTEEQEHYQRPK
2.2	2153.8699	0.0089	KLHFYLLAMELYSDR
2.2	2153.8780	0.0008	GQSDSGISRQDLDSFNIR
2.2	2153.8780	0.0008	GQSDSGISRQDLDSFNIR

Mascot: http://www.matrixscience.com/

Mascot Search Results

Peptide View

MS/MS Fragmentation of **QNSSSSDSGGSIVR**
Found in **IPI00290337**, Tax_Id=9606 Gene_Symbol=EPS8 Epidermal growth factor receptor kinase substrate 8

Match to Query 2516: 1545.588172 from(773.801362,2+)
Title: RawFile: CZ_140510_DC_EPS8_tio2_100517084708.raw FinneganScanNumber: 1403 _sil_
Data file C:\Users\cunningd\Documents\Mass Spec\Triple SILAC Experiments\070510 Eps8 FGF2 vs Eps8 FGF2 SU5402 vs Eps8 FGF2 Dasatinib + 170510 160610rpts enrich and FT\combined\allSpectra.CID.sil1_0.msm

Click mouse within plot area to zoom in by factor of two about that point
Or, Plot from 200 to 1500 Da Full range

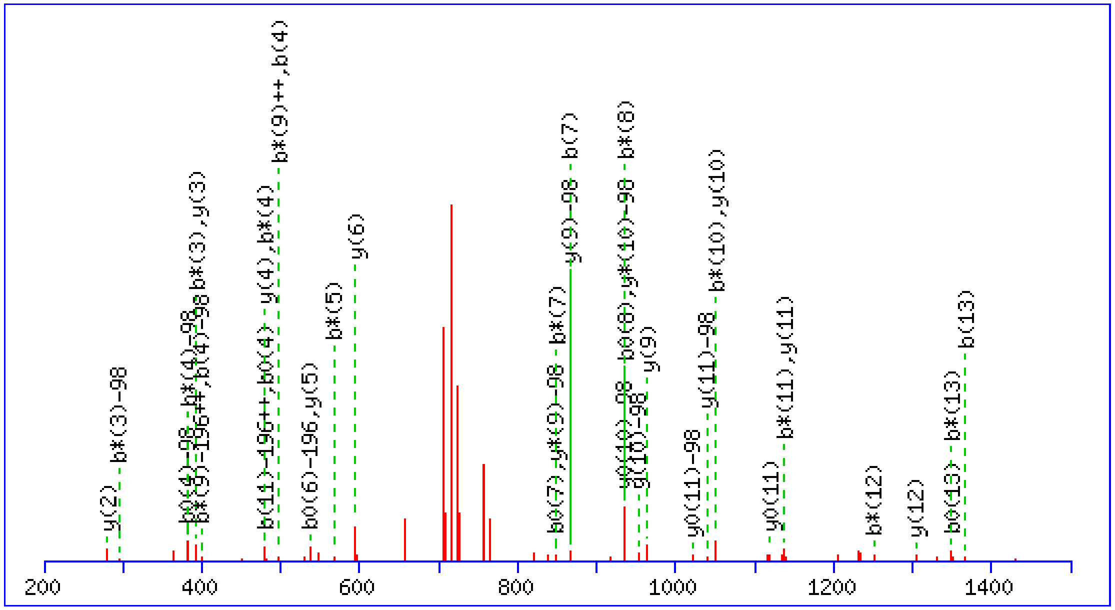

Monoisotopic mass of neutral peptide Mr(calc): 1545.5805
Fixed modifications: Carbamidomethyl (C),Label:13C(6) (R),Label:2H(4) (K)
Variable modifications:
S3 : Phospho (ST), with neutral losses 0.0000(shown in table), 97.9769
S6 : Phospho (ST), with neutral losses 0.0000(shown in table), 97.9769
Ions Score: 34 **Expect:** 0.013
Matches (Bold Red): 41/242 fragment ions using 64 most intense peaks

#	b	b^{++}	b^{*}	b^{*++}	b^{0}	b^{0++}	Seq.	y	y^{++}	y^{*}	y^{*++}	y^{0}	y^{0++}	#
1	129.0659	65.0366	112.0393	56.5233			Q							14
2	243.1088	122.0580	226.0822	113.5448			N	1418.5292	709.7682	1401.5026	701.2550	1400.5186	700.7629	13
3	410.1071	205.5572	**393.0806**	197.0439	392.0966	196.5519	S	**1304.4862**	652.7468	1287.4597	644.2335	1286.4757	643.7415	12
4	**497.1392**	249.0732	**480.1126**	240.5599	**479.1286**	240.0679	S	**1137.4879**	569.2476	1120.4613	560.7343	**1119.4773**	560.2423	11
5	584.1712	292.5892	**567.1446**	284.0760	566.1606	283.5840	S	**1050.4559**	525.7316	1033.4293	517.2183	1032.4453	516.7263	10
6	751.1695	376.0884	734.1430	367.5751	733.1590	367.0831	S	**963.4238**	482.2156	946.3973	473.7023	945.4133	473.2103	9
7	**866.1965**	433.6019	**849.1699**	425.0886	**848.1859**	424.5966	D	796.4255	398.7164	779.3989	390.2031	778.4149	389.7111	8
8	953.2285	477.1179	**936.2020**	468.6046	**935.2179**	468.1126	S	681.3985	341.2029	664.3720	332.6896	663.3880	332.1976	7
9	1010.2500	505.6286	993.2234	**497.1154**	992.2394	496.6233	G	**594.3665**	297.6869	577.3400	289.1736	576.3560	288.6816	6
10	1067.2714	534.1394	**1050.2449**	525.6261	1049.2609	525.1341	G	**537.3451**	269.1762	520.3185	260.6629	519.3345	260.1709	5
11	1154.3035	577.6554	**1137.2769**	569.1421	1136.2929	568.6501	S	**480.3236**	240.6654	463.2970	232.1522	462.3130	231.6601	4
12	1267.3875	634.1974	**1250.3610**	625.6841	1249.3770	625.1921	I	**393.2916**	197.1494	376.2650	188.6361			3
13	**1366.4559**	683.7316	**1349.4294**	675.2183	**1348.4454**	674.7263	V	**280.2075**	140.6074	263.1809	132.0941			2
14							R	181.1391	91.0732	164.1125	82.5599			1

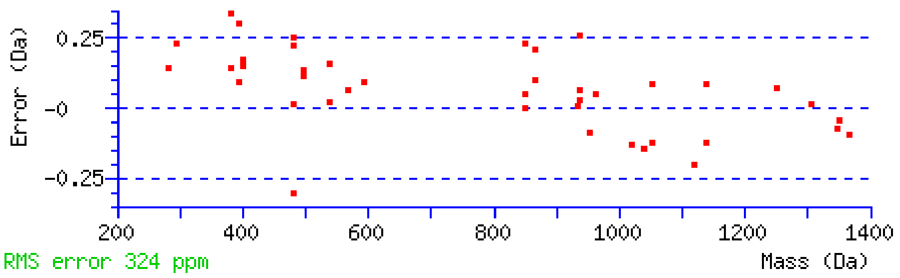

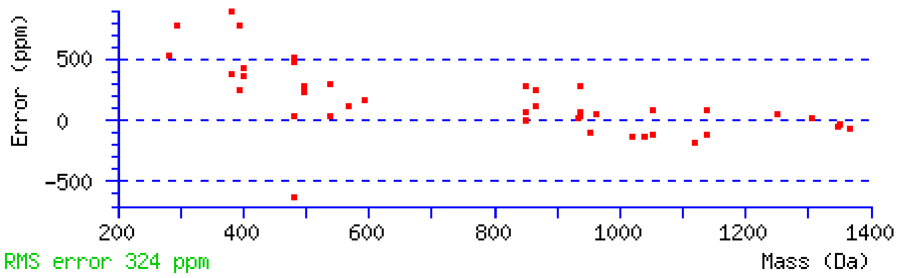

NCBI **BLAST** search of QNSSSSDSGGSIVR
(Parameters: blastp, nr protein database, expect=20000, no filter, PAM30)
Other BLAST web gateways

All matches to this query

Score	Mr(calc):	Delta	Sequence
34.1	1545.5805	0.0077	QNSSSSDSGGSIVR
31.9	1545.5805	0.0077	QNSSSSDSGGSIVR
31.9	1545.5805	0.0077	QNSSSSDSGGSIVR

31.7	1545.5805	0.0077	QNSSSSDSGGSIVR
28.9	1545.5805	0.0077	QNSSSSDSGGSIVR
28.9	1545.5805	0.0077	QNSSSSDSGGSIVR
26.6	1545.5805	0.0077	QNSSSSDSGGSIVR
21.2	1545.5805	0.0077	QNSSSSDSGGSIVR
20.9	1545.5805	0.0077	QNSSSSDSGGSIVR
16.2	1545.5805	0.0077	QNSSSSDSGGSIVR

Mascot: http://www.matrixscience.com/

Mascot Search Results

Peptide View

MS/MS Fragmentation of **DSVSSVSDISQYR**
Found in **IPI00290337**, Tax_Id=9606 Gene_Symbol=EPS8 Epidermal growth factor receptor kinase substrate 8

Match to Query 4139: 1601.607772 from(801.811162,2+)
Title: RawFile: CZ_070510_DC_EPS8_tio2.raw FinneganScanNumber: 1944 _sil_
Data file C:\Users\cunningd\Documents\Mass Spec\Triple SILAC Experiments\070510 Eps8 FGF2 vs Eps8 FGF2 SU5402 vs Eps8 FGF2 Dasatinib + 170510 160610rpts enrich and FT\combined\allSpectra.CID.sil0_0.msm

Click mouse within plot area to zoom in by factor of two about that point
Or, Plot from 200 to 1400 Da Full range

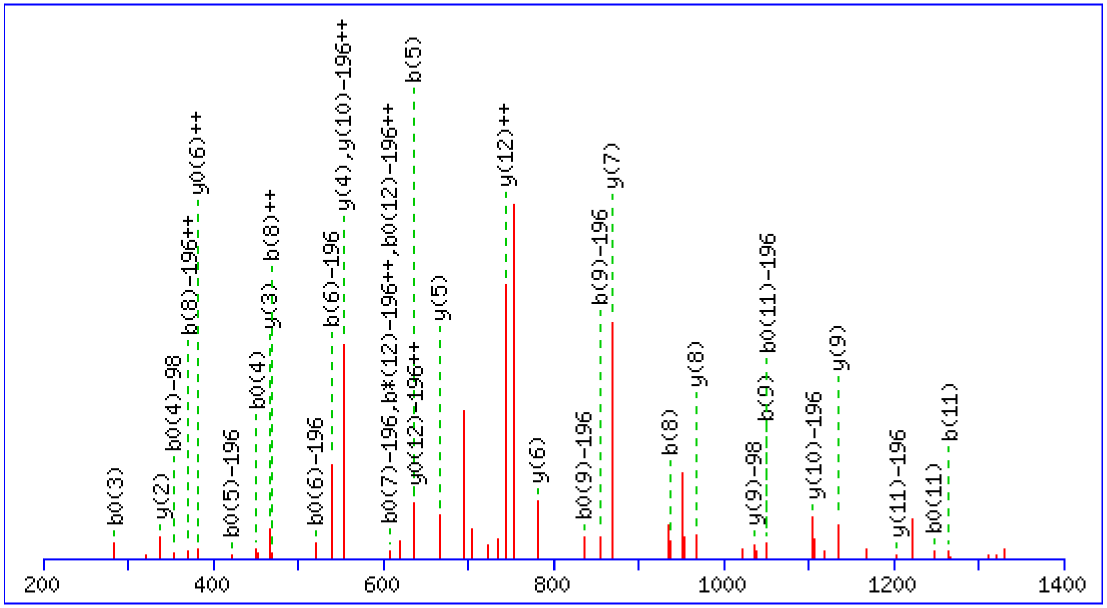

Monoisotopic mass of neutral peptide Mr(calc): 1601.6012
Fixed modifications: Carbamidomethyl (C)
Variable modifications:
S4 : Phospho (ST), with neutral losses 97.9769(shown in table), 0.0000
S5 : Phospho (ST), with neutral losses 97.9769(shown in table), 0.0000
Ions Score: 60 **Expect:** 5.8e-05
Matches (Bold Red): 36/182 fragment ions using 55 most intense peaks

#	b	b^{++}	b^{*}	b^{*++}	b^{0}	b^{0++}	Seq.	y	y^{++}	y^{*}	y^{*++}	y^{0}	y^{0++}	#
1	116.0342	58.5207			98.0237	49.5155	D							13
2	203.0662	102.0368			185.0557	93.0315	S	1291.6277	646.3175	1274.6011	637.8042	1273.6171	**637.3122**	12
3	302.1347	151.5710			**284.1241**	142.5657	V	**1204.5957**	602.8015	1187.5691	594.2882	1186.5851	593.7962	11
4	**371.1561**	186.0817			**353.1456**	177.0764	S	**1105.5272**	**553.2673**	1088.5007	544.7540	1087.5167	544.2620	10
5	440.1776	220.5924			**422.1670**	211.5871	S	**1036.5058**	518.7565	1019.4792	510.2433	1018.4952	509.7513	9
6	**539.2460**	270.1266			**521.2354**	261.1213	V	**967.4843**	484.2458	950.4578	475.7325	949.4738	475.2405	8
7	626.2780	313.6426			**608.2674**	304.6374	S	**868.4159**	434.7116	851.3894	426.1983	850.4054	425.7063	7
8	741.3050	**371.1561**			723.2944	362.1508	D	**781.3839**	391.1956	764.3573	382.6823	763.3733	**382.1903**	6
9	**854.3890**	427.6981			**836.3785**	418.6929	I	**666.3570**	333.6821	649.3304	325.1688	648.3464	324.6768	5
10	941.4210	471.2142			923.4105	462.2089	S	**553.2729**	277.1401	536.2463	268.6268	535.2623	268.1348	4
11	1069.4796	535.2435	1052.4531	526.7302	**1051.4691**	526.2382	Q	**466.2409**	233.6241	449.2143	225.1108			3
12	1232.5430	616.7751	1215.5164	**608.2618**	1214.5324	**607.7698**	Y	**338.1823**	169.5948	321.1557	161.0815			2
13							R	175.1190	88.0631	158.0924	79.5498			1

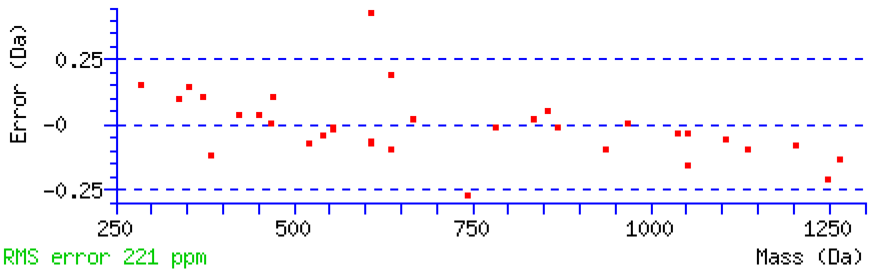

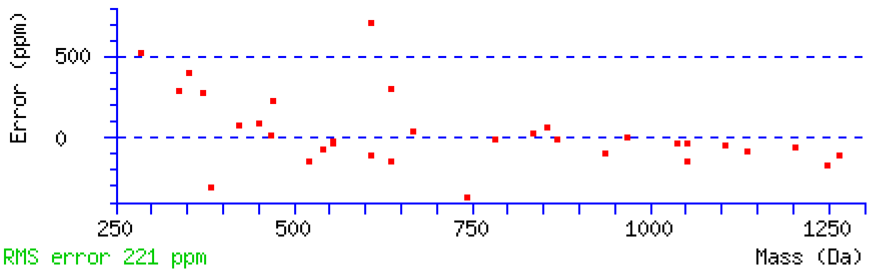

NCBI **BLAST** search of DSVSSVSDISQYR
(Parameters: blastp, nr protein database, expect=20000, no filter, PAM30)
Other BLAST web gateways

All matches to this query

Score	Mr(calc):	Delta	Sequence
60.5	1601.6012	0.0066	DSVSSVSDISQYR
60.1	1601.6012	0.0066	DSVSSVSDISQYR
47.1	1601.6012	0.0066	DSVSSVSDISQYR
36.0	1601.6012	0.0066	DSVSSVSDISQYR

24.3	1601.6012	0.0066	DSVSSVSDISQYR
14.4	1601.6012	0.0066	DSVSSVSDISQYR
12.2	1601.6012	0.0066	DSVSSVSDISQYR
8.7	1601.6012	0.0066	DSVSSVSDISQYR
7.3	1601.6150	-0.0072	KDAMTGAHSASMCR
6.5	1601.6012	0.0066	DSVSSVSDISQYR

Mascot: http://www.matrixscience.com/

Mascot Search Results

Peptide View

MS/MS Fragmentation of **QNSSSSDSGGSIVR**
Found in **IPI00290337**, Tax_Id=9606 Gene_Symbol=EPS8 Epidermal growth factor receptor kinase substrate 8

Match to Query 4225: 1459.598316 from(730.806434,2+)
Title: RawFile: CZ_270510_DC_eps8A.raw FinneganScanNumber: 682 _sil_
Data file C:\Users\cunningd\Documents\Mass Spec\Triple SILAC Experiments\210510+270510 rpts Eps8 FGF2 vs Eps8 FGF2 SU5402 vs Eps8 FGF2 Dasatinib\A\combined\allSpectra.CID.sil0_0.msm

Click mouse within plot area to zoom in by factor of two about that point
Or, Plot from 200 to 1300 Da Full range

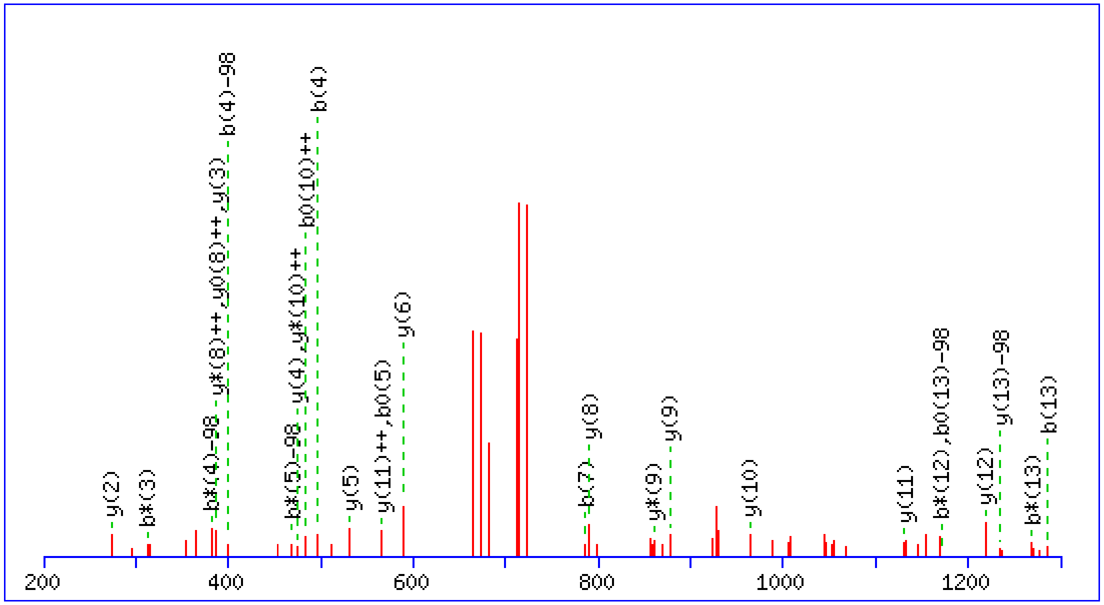

Monoisotopic mass of neutral peptide Mr(calc): 1459.5940
Fixed modifications: Carbamidomethyl (C)
Variable modifications:
S4 : Phospho (ST), with neutral losses 0.0000(shown in table), 97.9769
Ions Score: 50 **Expect:** 0.00075
Matches (Bold Red): 28/224 fragment ions using 56 most intense peaks

#	b	b^{++}	b^{*}	b^{*++}	b^{0}	b^{0++}	Seq.	y	y^{++}	y^{*}	y^{*++}	y^{0}	y^{0++}	#
1	129.0659	65.0366	112.0393	56.5233			Q							14
2	243.1088	122.0580	226.0822	113.5448			N	1332.5427	666.7750	1315.5162	658.2617	1314.5322	657.7697	13
3	330.1408	165.5740	**313.1143**	157.0608	312.1302	156.5688	S	**1218.4998**	609.7535	1201.4732	601.2403	1200.4892	600.7483	12
4	**497.1392**	249.0732	480.1126	240.5599	479.1286	240.0679	S	**1131.4678**	**566.2375**	1114.4412	557.7242	1113.4572	557.2322	11
5	584.1712	292.5892	567.1446	284.0760	**566.1606**	283.5840	S	**964.4694**	482.7383	947.4429	**474.2251**	946.4588	473.7331	10
6	671.2032	336.1052	654.1767	327.5920	653.1927	327.1000	S	**877.4374**	439.2223	**860.4108**	430.7091	859.4268	430.2170	9
7	**786.2302**	393.6187	769.2036	385.1054	768.2196	384.6134	D	**790.4054**	395.7063	773.3788	**387.1930**	772.3948	**386.7010**	8
8	873.2622	437.1347	856.2356	428.6215	855.2516	428.1294	S	675.3784	338.1928	658.3519	329.6796	657.3678	329.1876	7
9	930.2837	465.6455	913.2571	457.1322	912.2731	456.6402	G	**588.3464**	294.6768	571.3198	286.1636	570.3358	285.6715	6
10	987.3051	494.1562	970.2786	485.6429	969.2946	**485.1509**	G	**531.3249**	266.1661	514.2984	257.6528	513.3144	257.1608	5
11	1074.3371	537.6722	1057.3106	529.1589	1056.3266	528.6669	S	**474.3035**	237.6554	457.2769	229.1421	456.2929	228.6501	4
12	1187.4212	594.2142	**1170.3947**	585.7010	1169.4106	585.2090	I	**387.2714**	194.1394	370.2449	185.6261			3
13	**1286.4896**	643.7484	**1269.4631**	635.2352	1268.4791	634.7432	V	**274.1874**	137.5973	257.1608	129.0840			2
14							R	175.1190	88.0631	158.0924	79.5498			1

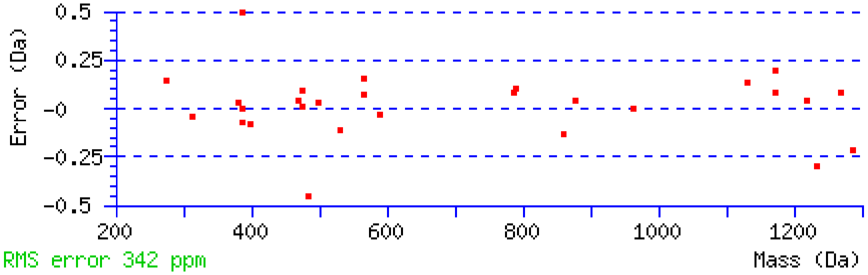

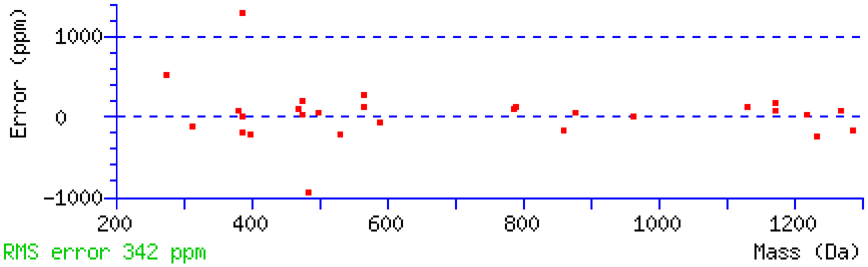

NCBI **BLAST** search of QNSSSSDSGGSIVR
(Parameters: blastp, nr protein database, expect=20000, no filter, PAM30)
Other BLAST web gateways

All matches to this query

Score	Mr(calc):	Delta	Sequence
49.6	1459.5940	0.0043	QNSSSSDSGGSIVR
38.2	1459.5940	0.0043	QNSSSSDSGGSIVR
33.6	1459.5940	0.0043	QNSSSSDSGGSIVR
22.6	1459.5940	0.0043	QNSSSSDSGGSIVR

22.4	1459.5940	0.0043	QNSSSSDSGGSIVR
8.3	1459.5940	0.0043	QNSSSSDSGGSIVR
6.0	1459.5989	-0.0006	CMTHFIVASTR
5.8	1459.5940	0.0043	VISGGSDSSSSNQR
5.8	1459.5940	0.0043	VISGGSDSSSSNQR
5.2	1459.5949	0.0034	KCEVLCSQSNR

Mascot: http://www.matrixscience.com/

Mascot Search Results

Peptide View

MS/MS Fragmentation of **DSVSSVSDISQYR**
Found in **IPI00290337**, Tax_Id=9606 Gene_Symbol=EPS8 Epidermal growth factor receptor kinase substrate 8

Match to Query 4761: 1521.637524 from(508.219784,3+)
Title: RawFile: CZ_270810_eps81a.raw FinneganScanNumber: 1867 _sil_
Data file C:\Users\cunningd\Documents\Mass Spec\Triple SILAC Experiments\270810 NO NaP Eps8 FGF2 vs Eps8 FGF2SU5402 vs Eps8 FGF2 Dasatinib\Expt 1 and expt 2\combined\allSpectra.CID.sil0_0.msm

Click mouse within plot area to zoom in by factor of two about that point
Or, Plot from 200 to 1300 Da Full range

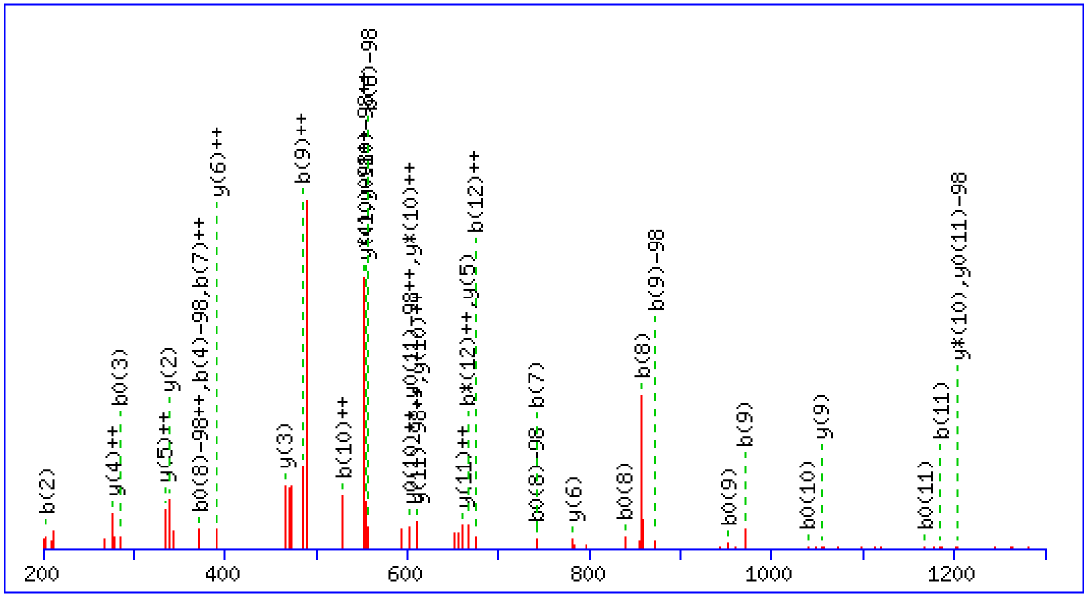

Monoisotopic mass of neutral peptide Mr(calc): 1521.6348
Fixed modifications: Carbamidomethyl (C)
Variable modifications:
S4 : Phospho (ST), with neutral losses 0.0000(shown in table), 97.9769
Ions Score: 33 **Expect:** 0.051
Matches (Bold Red): 39/176 fragment ions using 48 most intense peaks

#	b	b++	b*	b*++	b0	b0++	Seq.	y	y++	y*	y*++	y0	y0++	#

**1**	116.0342	58.5207			98.0237	49.5155	**D**							**13**
**2**	**203.0662**	102.0368			185.0557	93.0315	**S**	1407.6152	704.3112	1390.5886	695.7979	1389.6046	695.3059	**12**
**3**	302.1347	151.5710			**284.1241**	142.5657	**V**	1320.5831	**660.7952**	1303.5566	652.2819	1302.5726	651.7899	**11**
**4**	469.1330	235.0701			451.1224	226.0649	**S**	1221.5147	**611.2610**	**1204.4882**	**602.7477**	1203.5042	**602.2557**	**10**
**5**	556.1650	278.5862			538.1545	269.5809	**S**	**1054.5164**	527.7618	1037.4898	519.2485	1036.5058	518.7565	**9**
**6**	655.2335	328.1204			637.2229	319.1151	**V**	967.4843	484.2458	950.4578	475.7325	949.4738	475.2405	**8**
**7**	**742.2655**	**371.6364**			724.2549	362.6311	**S**	868.4159	434.7116	851.3894	426.1983	850.4054	425.7063	**7**
**8**	**857.2924**	429.1499			**839.2819**	420.1446	**D**	**781.3839**	**391.1956**	764.3573	382.6823	763.3733	382.1903	**6**
**9**	**970.3765**	**485.6919**			**952.3659**	476.6866	**I**	**666.3570**	**333.6821**	649.3304	325.1688	648.3464	324.6768	**5**
**10**	1057.4085	**529.2079**			**1039.3980**	520.2026	**S**	**553.2729**	**277.1401**	536.2463	268.6268	535.2623	268.1348	**4**
**11**	**1185.4671**	593.2372	1168.4405	584.7239	**1167.4565**	584.2319	**Q**	**466.2409**	233.6241	449.2143	225.1108			**3**
**12**	1348.5304	**674.7689**	1331.5039	**666.2556**	1330.5199	665.7636	**Y**	**338.1823**	169.5948	321.1557	161.0815			**2**
**13**							**R**	175.1190	88.0631	158.0924	79.5498			**1**

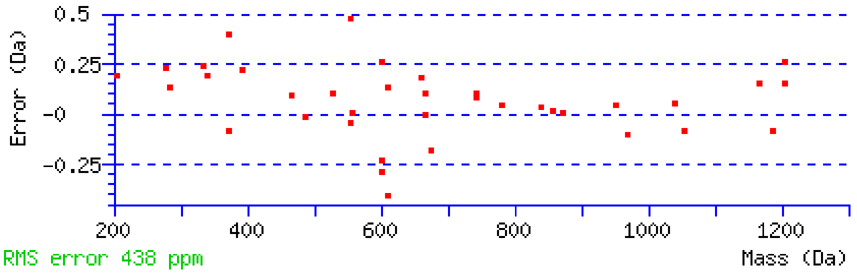

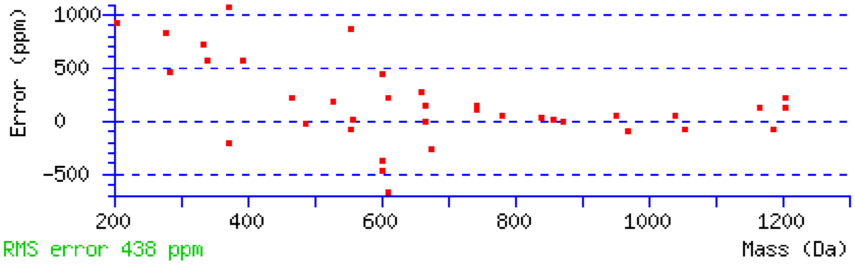

NCBI **BLAST** search of DSVSSVSDISQYR
(Parameters: blastp, nr protein database, expect=20000, no filter, PAM30)
Other BLAST web gateways

All matches to this query

Score	Mr(calc):	Delta	Sequence
32.7	1521.6348	0.0027	DSVSSVSDISQYR
28.4	1521.6348	0.0027	DSVSSVSDISQYR
26.8	1521.6348	0.0027	DSVSSVSDISQYR
25.8	1521.6348	0.0027	DSVSSVSDISQYR
10.5	1521.6296	0.0079	AWAHSCTGSPRGR
0.9	1521.6357	0.0019	AMLSSTAMYSAPGR

0.8	1521.6283	0.0092	KMPGESHEESGVR
0.6	1521.6306	0.0069	YFIIESHPTQK

Mascot: http://www.matrixscience.com/

Mascot Search Results

Peptide View

MS/MS Fragmentation of **AALEDSSGSSELQEIMR**
Found in **IPI00290337**, Tax_Id=9606 Gene_Symbol=EPS8 Epidermal growth factor receptor kinase substrate 8

Match to Query 3677: 1907.828916 from(954.921734,2+)
Title: RawFile: CZ_270810_eps82b.raw FinneganScanNumber: 1968 _sil_
Data file C:\Users\cunningd\Documents\Mass Spec\Triple SILAC Experiments\270810 NO NaP Eps8 FGF2 vs Eps8 FGF2SU5402 vs Eps8 FGF2 Dasatinib\Expt 1 and expt 2\combined\allSpectra.CID.sil1_0.msm

Click mouse within plot area to zoom in by factor of two about that point
Or, Plot from 300 to 1800 Da Full range

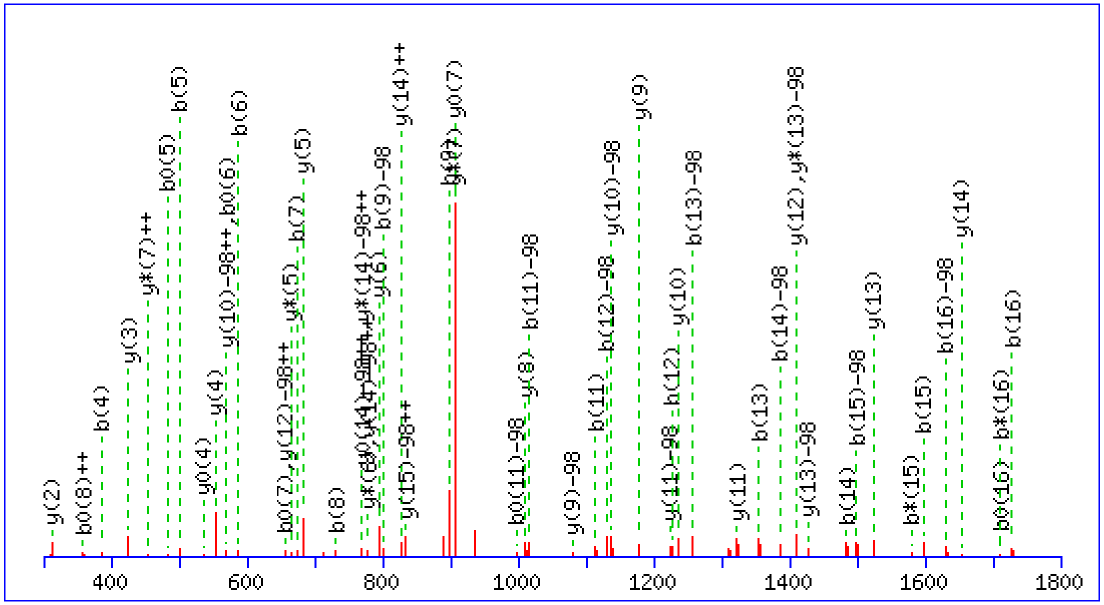

Monoisotopic mass of neutral peptide Mr(calc): 1907.8279
Fixed modifications: Carbamidomethyl (C),Label:13C(6) (R),Label:2H(4) (K)
Variable modifications:
S9 : Phospho (ST), with neutral losses 97.9769(shown in table), 0.0000
Ions Score: 72 **Expect:** 6.1e-06
Matches (Bold Red): 57/244 fragment ions using 84 most intense peaks

#	b	b^{++}	b^{*}	b^{*++}	b^{0}	b^{0++}	Seq.	y	y^{++}	y^{*}	y^{*++}	y^{0}	y^{0++}	#
1	72.0444	36.5258					A							17
2	143.0815	72.0444					A	1739.8212	870.4142	1722.7946	861.9010	1721.8106	861.4089	16
3	256.1656	128.5864					L	1668.7841	**834.8957**	1651.7575	826.3824	1650.7735	825.8904	15
4	**385.2082**	193.1077			367.1976	184.1024	E	1555.7000	**778.3536**	1538.6734	**769.8404**	1537.6894	**769.3484**	14
5	**500.2351**	250.6212			**482.2245**	241.6159	D	**1426.6574**	713.8323	**1409.6309**	705.3191	1408.6468	704.8271	13
6	**587.2671**	294.1372			**569.2566**	285.1319	S	1311.6305	**656.3189**	1294.6039	647.8056	1293.6199	647.3136	12
7	**674.2992**	337.6532			**656.2886**	328.6479	S	**1224.5984**	612.8029	1207.5719	604.2896	1206.5879	603.7976	11
8	**731.3206**	366.1640			713.3101	**357.1587**	G	**1137.5664**	**569.2868**	1120.5399	560.7736	1119.5558	560.2816	10
9	**800.3421**	400.6747			782.3315	391.6694	S	**1080.5449**	540.7761	1063.5184	532.2628	1062.5344	531.7708	9
10	887.3741	444.1907			869.3635	435.1854	S	**1011.5235**	506.2654	994.4969	497.7521	993.5129	497.2601	8
11	**1016.4167**	508.7120			**998.4061**	499.7067	E	924.4915	462.7494	**907.4649**	**454.2361**	**906.4809**	453.7441	7
12	**1129.5008**	565.2540			1111.4902	556.2487	L	**795.4489**	398.2281	**778.4223**	389.7148	777.4383	389.2228	6
13	**1257.5593**	629.2833	1240.5328	620.7700	1239.5488	620.2780	Q	**682.3648**	341.6860	**665.3383**	333.1728	664.3542	332.6808	5
14	**1386.6019**	693.8046	1369.5754	685.2913	1368.5914	684.7993	E	**554.3062**	277.6567	537.2797	269.1435	**536.2957**	268.6515	4
15	**1499.6860**	750.3466	1482.6595	741.8334	1481.6754	741.3414	I	**425.2636**	213.1355	408.2371	204.6222			3
16	**1630.7265**	815.8669	1613.6999	807.3536	1612.7159	806.8616	M	**312.1796**	156.5934	295.1530	148.0801			2
17							R	181.1391	91.0732	164.1125	82.5599			1

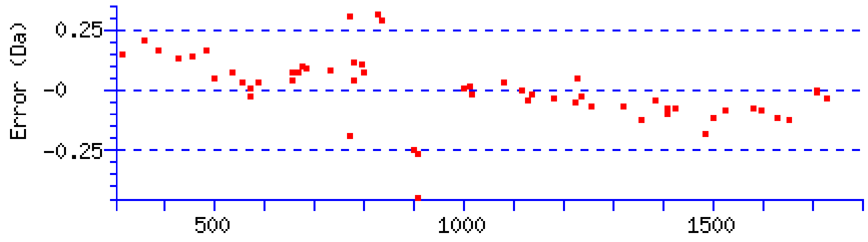

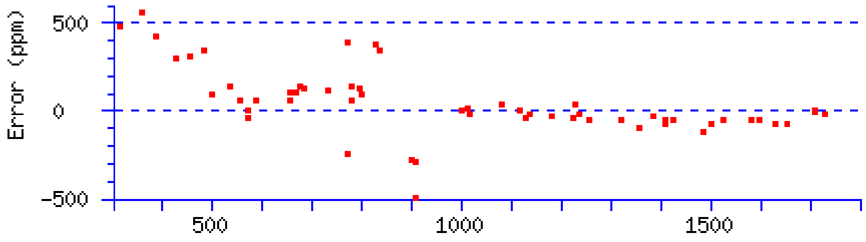

NCBI **BLAST** search of AALEDSSGSSELQEIMR
(Parameters: blastp, nr protein database, expect=20000, no filter, PAM30)
Other BLAST web gateways

All matches to this query

Score	Mr(calc):	Delta	Sequence
72.3	1907.8279	0.0010	AALEDSSGSSELQEIMR

65.9	1907.8279	0.0010	AALEDSSGSSELQEIMR
63.9	1907.8279	0.0010	AALEDSSGSSELQEIMR
60.4	1907.8279	0.0010	AALEDSSGSSELQEIMR
9.2	1907.8366	-0.0077	AGSQVMMQLSNEPFQR
7.5	1907.8163	0.0126	TGYTVTGSGTTALPGGFR
7.0	1907.8163	0.0126	TGYTVTGSGTTALPGGFR
5.0	1907.8163	0.0126	TGYTVTGSGTTALPGGFR
1.8	1907.8366	-0.0077	AGSQVMMQLSNEPFQR
1.3	1907.8274	0.0015	GTLAASAEPYDGAAMAGGSK

Mascot: http://www.matrixscience.com/

Mascot Search Results

Peptide View

MS/MS Fragmentation of **GSHLDQGEAAVAFK**
Found in **IPI00290337**, Tax_Id=9606 Gene_Symbol=EPS8 Epidermal growth factor receptor kinase substrate 8

Match to Query 5234: 1516.682883 from(506.568237,3+)
Title: RawFile: CZ_270810_eps82b.raw FinneganScanNumber: 1582 _sil_
Data file C:\Users\cunningd\Documents\Mass Spec\Triple SILAC Experiments\270810 NO NaP Eps8 FGF2 vs Eps8 FGF2SU5402 vs Eps8 FGF2 Dasatinib\Expt 1 and expt 2\combined\allSpectra.CID.sil2_0.msm

Click mouse within plot area to zoom in by factor of two about that point
Or, [Plot from] [200] to [1400] Da [Full range]

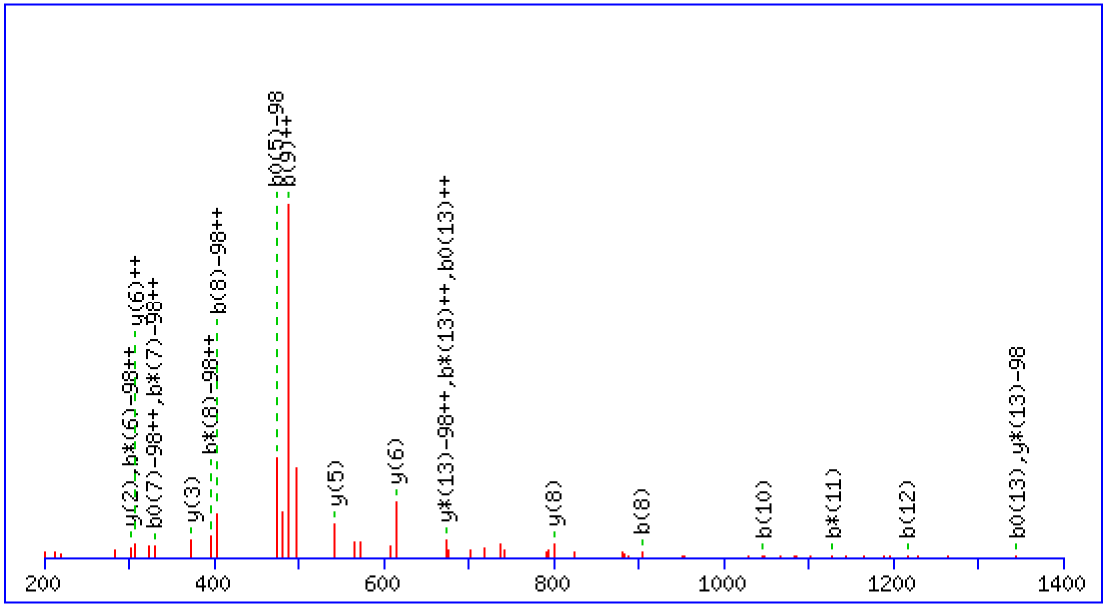

Monoisotopic mass of neutral peptide Mr(calc): 1516.6803
Fixed modifications: Carbamidomethyl (C),Label:13C(6)15N(4) (R),Label:13C(6)15N (2) (K)
Variable modifications:
S2 : Phospho (ST), with neutral losses 0.0000(shown in table), 97.9769
Ions Score: 18 **Expect:** 1.5
Matches (Bold Red): 22/202 fragment ions using 33 most intense peaks

#	b	b++	b*	b***	b0	b0++	Seq.	y	y++	y*	y***	y0	y0++	#
1	58.0287	29.5180					G							14
2	225.0271	113.0172			207.0165	104.0119	S	1460.6661	730.8367	1443.6395	722.3234	1442.6555	721.8314	13
3	362.0860	181.5466			344.0754	172.5414	H	1293.6677	647.3375	1276.6412	638.8242	1275.6572	638.3322	12
4	475.1701	238.0887			457.1595	229.0834	L	1156.6088	578.8080	1139.5823	570.2948	1138.5983	569.8028	11
5	590.1970	295.6021			572.1864	286.5969	D	1043.5248	522.2660	1026.4982	513.7527	1025.5142	513.2607	10
6	718.2556	359.6314	701.2290	351.1182	700.2450	350.6261	Q	928.4978	464.7525	911.4713	456.2393	910.4872	455.7473	9
7	775.2771	388.1422	758.2505	379.6289	757.2665	379.1369	G	**800.4392**	400.7233	783.4127	392.2100	782.4287	391.7180	8
8	**904.3196**	452.6635	887.2931	444.1502	886.3091	443.6582	E	743.4178	372.2125	726.3912	363.6992	725.4072	363.2072	7
9	975.3568	**488.1820**	958.3302	479.6687	957.3462	479.1767	A	**614.3752**	**307.6912**	597.3486	299.1780			6
10	**1046.3939**	523.7006	1029.3673	515.1873	1028.3833	514.6953	A	**543.3381**	272.1727	526.3115	263.6594			5
11	1145.4623	573.2348	**1128.4357**	564.7215	1127.4517	564.2295	V	472.3009	236.6541	455.2744	228.1408			4
12	**1216.4994**	608.7533	1199.4729	600.2401	1198.4888	599.7481	A	**373.2325**	187.1199	356.2060	178.6066			3
13	1363.5678	682.2875	1346.5413	**673.7743**	**1345.5573**	**673.2823**	F	**302.1954**	151.6013	285.1689	143.0881			2
14							K	155.1270	78.0671	138.1005	69.5539			1

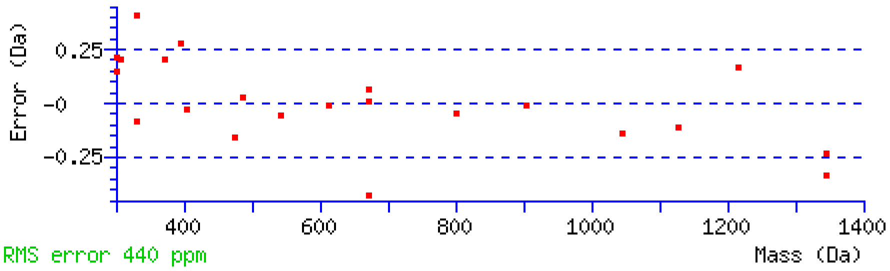

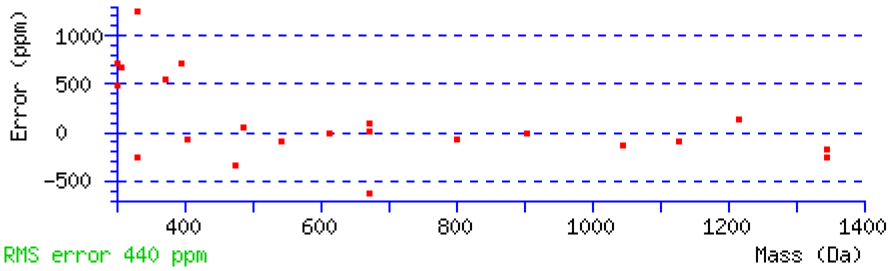

NCBI **BLAST** search of GSHLDQGEAAVAFK
(Parameters: blastp, nr protein database, expect=20000, no filter, PAM30)
Other BLAST web gateways

All matches to this query

Score	Mr(calc):	Delta	Sequence
17.7	1516.6803	0.0026	GSHLDQGEAAVAFK
1.8	1516.6807	0.0022	KPLVPSNQDSVR
0.7	1516.6741	0.0088	ACQSGETQLMEQK

0.3	1516.6748	0.0081	FEEECVVHDAFK
0.2	1516.6815	0.0014	EAQKISDDLMQK
0.2	1516.6756	0.0073	LQPGGLRYLPYX

Mascot: http://www.matrixscience.com/
