# Supplementary material for: Novel Binding Partners and Differentially Regulated Phosphorylation Sites Clarify Eps8 as a Multi-Functional Adaptor
Source: PLoS One. 2013 Apr 23;8(4):e61513. doi: 10.1371/journal.pone.0061513 (PMC3634024; doi:10.1371/journal.pone.0061513)
Supplement: Method S1 — Supplementary details regarding Peptide and Protein Identification and Quantification. (DOC) [file pone.0061513.s006.doc]

**Method S1 – Supplementary Details Regarding Peptide and Protein Identification and Quantitation**

*SILAC triple labelled anti-Eps8 IP to compare FGF2 stimulation in the presence or absence of either SU5402 or dasatinib*

Three biological replicate experiments were carried out. For experiment one, each non-enriched sample was analysed once via mass spectrometry, and the phosphopeptide enriched Eps8 sample and flow through was analysed three times. This resulted in 18 raw files. For experiments two and three, each sample was analysed twice via mass spectrometry. This resulted in 20 and 22 raw files, respectively. In addition, control samples for each experiment comprising of equal amounts of WCL were combined and run on an SDS gel. Following Coomassie staining, two gel slices were cut out, trypsin digested, and analysed via mass spectrometry. All ratios obtained from MaxQuant were adjusted using a correction factor to allow for mixing error. This correction factor was calculated for each experiment by combining the control samples and normalising peptide ratios so that the median of their logarithms is zero. In order to calculate the probability cut-offs (p = 0.05) for determining the cut-off for statistical significance of differential phosphorylation on Eps8, the value for 2 x the standard deviation of the control ratio logarithms was calculated. Ratios were deemed significantly changed if they were < 0.57 or > 1.75. The correction factor was then applied to all experimental samples, and the medians of the corrected ratio logarithms for each peptide were calculated.

Table S1 was created from the evidence.txt output.

*Eps8 SILAC peptide pull downs*

For each peptide pair one experiment was carried out and each sample was analysed twice via mass spectrometry. This resulted in 20 raw files per peptide pair. Table S2 was created from the proteingroups.txt output.
